# Efficacy, Immunogenicity, and Safety of COVID-19 Vaccines in Patients with Autoimmune Diseases: A Systematic Review and Meta-Analysis

**DOI:** 10.3390/vaccines11091456

**Published:** 2023-09-04

**Authors:** Alvina Widhani, Anshari Saifuddin Hasibuan, Retia Rismawati, Suzy Maria, Sukamto Koesnoe, Muhammad Ikrar Hermanadi, Youdiil Ophinni, Chika Yamada, Kuntjoro Harimurti, Aldean Nadhyia Laela Sari, Evy Yunihastuti, Samsuridjal Djauzi

**Affiliations:** 1 Allergy and Clinical Immunology Division, Department of Internal Medicine, Faculty of Medicine, Universitas Indonesia, Dr. Cipto Mangunkusumo Hospital, Jakarta 10430, Indonesia; sorihsb@gmail.com (A.S.H.); retiarismawati@gmail.com (R.R.); suzyduri@gmail.com (S.M.); sukamtokoesnoe@gmail.com (S.K.); ikrar.hermanadi@gmail.com (M.I.H.); aldeannadhyia@gmail.com (A.N.L.S.); evy.yunihastuti@gmail.com (E.Y.); samsuridjal@yahoo.com (S.D.); 2 Department of Internal Medicine, Universitas Indonesia Hospital, Depok 16424, Indonesia; 3 Division of Clinical Virology, Center for Infectious Diseases, Graduate School of Medicine, Kobe University, Kobe 650-0017, Japan; yophinni@panda.kobe-u.ac.jp; 4 Department of Host Defense, Immunology Frontier Research Center, Osaka University, Osaka 565-0871, Japan; 5 Center for Southeast Asian Studies, Kyoto University, Kyoto 606-8304, Japan; chika128@cseas.kyoto-u.ac.jp; 6 Geriatric Division, Department of Internal Medicine, Faculty of Medicine, Universitas Indonesia, Dr. Cipto Mangunkusumo Hospital, Jakarta 10430, Indonesia; kuntjoro.harimurti@gmail.com

**Keywords:** autoimmune, efficacy, immunogenicity, safety, vaccine, COVID-19

## Abstract

Patients with autoimmune diseases are among the susceptible groups to COVID-19 infection because of the complexity of their conditions and the side effects of the immunosuppressive drugs used to treat them. They might show impaired immunogenicity to COVID-19 vaccines and have a higher risk of developing COVID-19. Using a systematic review and meta-analysis, this research sought to summarize the evidence on COVID-19 vaccine efficacy, immunogenicity, and safety in patients with autoimmune diseases following predefined eligibility criteria. Research articles were obtained from an initial search up to 26 September 2022 from PubMed, Embase, EBSCOhost, ProQuest, MedRxiv, bioRxiv, SSRN, EuroPMC, and the Cochrane Center of Randomized Controlled Trials (CCRCT). Of 76 eligible studies obtained, 29, 54, and 38 studies were included in systematic reviews of efficacy, immunogenicity, and safety, respectively, and 6, 18, and 4 studies were included in meta-analyses for efficacy, immunogenicity, and safety, respectively. From the meta-analyses, patients with autoimmune diseases showed more frequent breakthrough COVID-19 infections and lower total antibody (TAb) titers, IgG seroconversion, and neutralizing antibodies after inactivated COVID-19 vaccination compared with healthy controls. They also had more local and systemic adverse events after the first dose of inactivated vaccination compared with healthy controls. After COVID-19 mRNA vaccination, patients with autoimmune diseases had lower TAb titers and IgG seroconversion compared with healthy controls.

## 1. Introduction

As of 26 December 2022, there were more than 651 million cases of COVID and more than 6 million deaths reported worldwide [1]. It is important to understand that certain groups in the population are higher-risk groups who are more susceptible to severe COVID-19 infection. These groups consist of people who have comorbidities, such as cancer, chronic kidney disease, underlying lung disorders, diabetes, dementia, cardiac issues, HIV, other immunocompromised conditions, neurological diseases, and pregnancy [2].

One of these susceptible groups is people with autoimmune diseases because of the complexity of these conditions and the mechanisms underlying the therapeutic effects of the drugs used to treat them. Medications play a pivotal role in significantly improving the disease course and outcomes of autoimmune patients. However, the primary disadvantage of these medications is the immunosuppressive effect they have, which can enhance the risk of infections. Therefore, there is an emerging demand to prioritize COVID-19 vaccination for people with autoimmune conditions, as this prevents severe disease outcomes [3,4].

Vaccination is an effort to suppress the case numbers and severity of COVID-19 infections [5,6]. It has been established that vaccines can induce humoral and/or cellular immune responses to build protection against various infectious diseases, which is an ability also known as immunogenicity [5,7]. Not only does COVID-19 vaccination protect healthy individuals from getting infected, it also prevents those who are infected from getting severely ill, or even dying, from COVID-19 [5,6,7]. As of 26 December 2022, 13 billion doses of COVID 19 vaccine had been administered worldwide [1].

An additional cause of concern is that patients with systemic autoimmune diseases might show impaired immunogenicity to COVID-19 vaccines. These patients can have a higher risk of developing COVID-19 [8]. Besides the issue of decreased vaccine efficacy due to the use of immunosuppressive drugs, the safety of the COVID-19 vaccine is also a concern among these patients [9,10]. Certain vaccine antigens and their adjuvants, such as aluminum salts (alum), have been claimed to induce autoimmunity in numerous studies. Adjuvants are usually needed in inactivated and recombinant protein vaccines to boost the immunogenicity induced by the antigen [4]. Patients with autoimmune diseases are more susceptible to vaccination-induced autoimmune/autoinflammatory syndrome induced by adjuvants (ASIA) [11]. SARS-CoV-2 amino acid sequences cross-react with human cell sequences [12]. The antibody to the S1 spike protein of SARS-CoV-2 has a high affinity for transglutaminase 3 protein, transglutaminase 2 protein, anti-extractable nuclear antigen, nuclear antigen, and myelin basic protein [13]. Despite the evidence, this claim should be interpreted cautiously, as the temporal relationship between the vaccine and autoimmune events is still unclear [4]. There is also evidence that non-live vaccines, including those for influenza and pneumococcal virus, do not cause exacerbation of previously diagnosed autoimmune conditions [3,6].

In the third-phase clinical trial of ChAdOx1 nCoV-19 (AstraZeneca), a simian adenovirus-vectored vaccine, there was one case of transverse myelitis reported 14 days after vaccination [14]. A cohort study from the health registry in Denmark and Norway showed an increase in venous thromboembolism cases, including cerebral venous thrombosis, 28 days after ChAdOx1 nCoV-19, and a slight increase in thrombocytopenia and bleeding cases [15]. Another study reported 39 patients with thrombocytopenia and thrombosis 5–24 days after vaccination with ChAdOx1 nCoV-19. These patients were diagnosed with vaccine-induced thrombotic thrombocytopenia (VITT) or thrombosis with thrombocytopenia syndrome (TTS), which were suspected to be caused by platelets activating antibodies to platelet factor 4 [16,17,18]. Brill et al. reported autoimmune hepatitis 6 days after administration of the Pfizer–BioNTech COVID-19 vaccine in a 35-year-old woman. This case report could not conclude whether this was a causal relationship or only coincidence [19]. There were also reports of thrombocytopenia post the mRNA vaccine, which were diagnosed as secondary immune thrombocytopenia (ITP), but again, it could not be determined whether this was a coincidence or vaccine-induced ITP [20].

Despite COVID-19 vaccination being recommended, the efficacy, immunogenicity, and safety of COVID-19 vaccination in people with autoimmune diseases have not been discussed much. In addition, patients with autoimmune conditions and/or people taking immunosuppressants were excluded from clinical trials of approved COVID-19 vaccines [4,21]. Therefore, this systematic review aims to summarize the evidence on COVID-19 vaccine efficacy, immunogenicity, and safety in autoimmune patients.

## 2. Materials and Methods

The protocol for this study has been registered in PROSPERO with the registration number CRD42022337621. This study was conducted in accordance with the Preferred Reporting Items of the Systematic Review and Meta-Analysis (PRISMA) checklist [22].

### 2.1. Eligibility Criteria

The specific inclusion criteria for the systematic review and meta-analysis were as follows: (1) all randomized controlled trials (RCTs), non-randomized studies of interventions, cohort studies, case–control studies, and cross-sectional studies; (2) studies with autoimmune patients as the population (with the autoimmune condition existing prior to the intervention); (3) COVID-19 vaccination as the intervention; (4) efficacy, immunogenicity or safety as outcomes; and (5) publication in English. The exclusion criteria were as follows: (1) full text or data that cannot be accessed even though the corresponding author has been contacted.

### 2.2. Information Sources and Search Strategy

We included all articles on patients with autoimmune diseases published in English from 2020 to 2022. Electronic databases were searched using PubMed, Embase, EBSCOhost, ProQuest, MedRxiv, bioRxiv, SSRN, EuroPMC, and the Cochrane Center of Randomized Controlled Trials (CCRCT) from 6–26 September 2022 for studies evaluating the response to SARS-CoV-2 vaccines using a combination of keywords and medical subject headings. The keywords utilized were “autoimmune”; “vaccine” or “immunization” or “vaccination”; “COVID-19”; “efficacy”; “immunogenicity”; and “safety” or “adverse event” or “adverse effect”, along with their synonyms and related terms incorporated by the appropriate Boolean operators. The detailed search strategy for articles is available in the Supplementary Materials (Table S1).

### 2.3. Data Extraction

Records were checked for duplicates using Zotero 6.0.19. Two independent researchers screened the literature search and assessed each study for inclusion by reading titles, abstracts, and full texts. Different opinions during data extraction were resolved by discussion and the inclusion of a study was decided by the two researchers. Relevant data were obtained from each eligible study by using an extraction sheet, which was prepared and approved by all the reviewers by reaching a consensus after screening for the eligible studies. Relevant data that were collected included study characteristics (authors, year, country, research setting, study design, study duration, sample size); participant characteristics (autoimmune diagnosis, age, sex, comorbidities); intervention (COVID-19 vaccine platform) and comparison; and outcomes (efficacy, immunogenicity, safety). Two independent researchers collected the data from each research article. The corresponding authors were contacted to obtain any information that was not explicitly available.

### 2.4. Outcome Measures

All studies describing the efficacy, immunogenicity, or safety of the COVID-19 vaccine in autoimmune patients were evaluated. The main outcomes were (1) breakthrough COVID-19 events, severity of infection, hospitalization, and mortality as markers of efficacy; (2) neutralizing antibodies, antibody titers, and seroconversion as markers of immunogenicity; and (3) flares or autoimmune relapses, local reactions, systemic reactions, and other adverse events as markers of safety.

The pooled efficacy, immunogenicity, and safety data after primary or booster doses of COVID-19 vaccine were evaluated. Efficacy was measured by the number of COVID-19 breakthrough infections, severity of COVID-19 infections, and hospitalizations and mortality related to COVID-19 infection. A COVID-19 breakthrough infection was defined as an infection after receiving the vaccination. Severity was defined by one of three levels of COVID-19 infection after vaccination: mild, moderate, or severe. Hospitalization was defined as the number of people who were taken to hospital as a result of COVID-19 infection. Mortality was defined as the number of people who died as a result of COVID-19 infection. Immunogenicity was defined as the ability of COVID-19 vaccines to stimulate an immune response, which was measured by the proportions of subjects with seroconversion (based on total IgG, as measured by ELISA) and with neutralizing antibodies (based on a plaque reduction neutralization test (PRNT) or surrogate virus neutralization test (sVNT), total IgG antibody titers (following WHO guidelines on translating results from different ELISA manufacturers into standardized binding antibody units (BAU)/mL) [23], and neutralizing activity (based on PRNT or sVNT, calculated as (1-OD value of sample/OD value of control) × 100%). Antibody titers were log-transformed prior to standardized mean difference (SMD) calculation. Where applicable, PRNT_50_ titer was correlated with sVNT inhibition capacity [24], mean and standard deviation (SD) were estimated from median and interquartile range (IQR) [25], SDs were estimated from 95% confidence interval, and means and SDs were aggregated from multiple subgroups. Safety was measured by the number of autoimmune relapses, local symptoms (pain, erythema, bruising, etc.), systemic symptoms (fever, joint pain, flu like symptoms, fatigue, headache, muscle pain), and other adverse events occurring after receipt of a dose of COVID-19 vaccine.

### 2.5. Risk of Bias Evaluation

Risk-of-bias and quality-of-study evaluations were carried out by two independent researchers. The Risk of Bias (RoB) and Risk of Bias in Nonrandomized Studies of Interventions (ROBINS-I) tools were used for randomized controlled trials (RCT) and non-randomized studies of interventions, respectively [26]. Cross-sectional and case-series studies were assessed using the Newcastle–Ottawa Quality Assessment Scales and The National Institutes of Health (NIH) quality assessment tool, respectively [27,28]. The certainty of evidence for the primary outcomes was evaluated using the Grades of Recommendation, Assessment, Development, and Evaluation (GRADE) system in eight domains: risk of bias, inconsistency, indirectness, imprecision, publication bias, large effects, plausible confounding, and dose–response gradient [26].

### 2.6. Data Synthesis

All outcomes were analyzed using Microsoft Excel and RevMan version 5.4 issued by Cochrane. Outcomes were reported as risk ratios for categorical data and standardized mean differences for numerical data, each with a confidence interval. Risk ratio was used to compare the risks of outcomes measured among patients with autoimmune diseases to healthy controls, while standardized mean difference was used to assess and pool continuous data, which was measured in a variety of ways. For analyzing continuous data conversion, guidelines from the Cochrane book were applied [28]. Heterogeneity was assessed using Higgins I^2^ and considered significant at I^2^ > 60% [28]. For significantly heterogeneous data, subgroup analysis was performed. Fixed-effects models were used for data with no substantial heterogeneity or which was considered homogeneous, whereas random effect models were used when there was significant heterogeneity. Data was displayed as a forest plot for meta-analysis. 

## 3. Results

**Figure 1:**
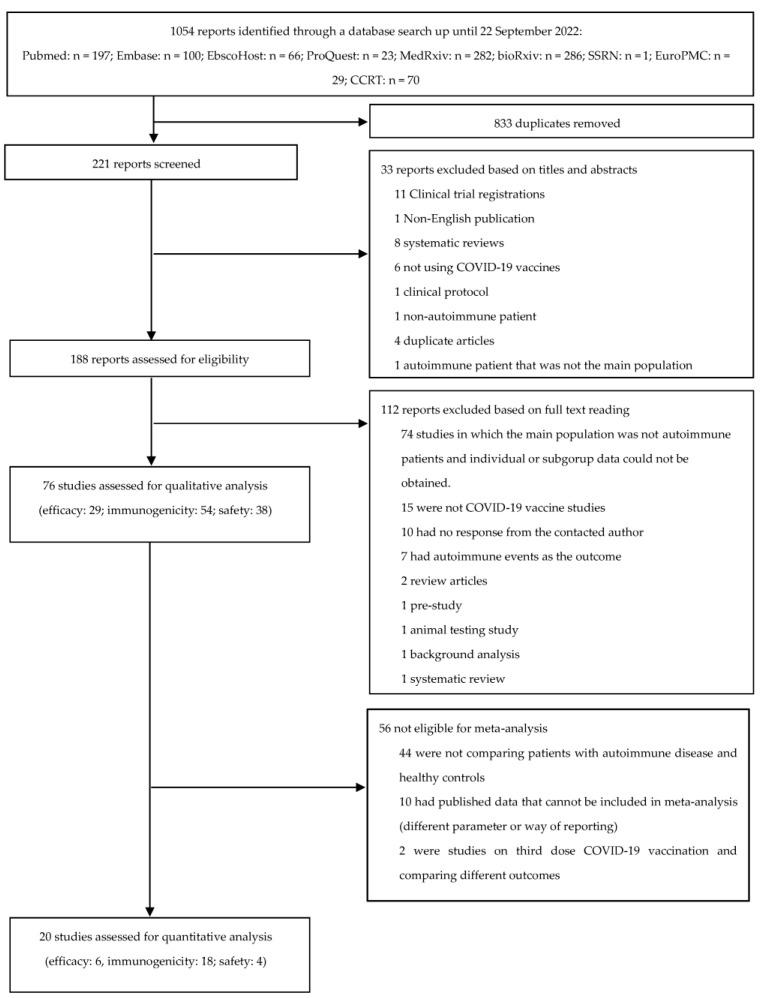
PRISMA flow chart.

Our search retrieved 1054 records, of which 833 were duplicates and were excluded. The titles and abstracts of the remaining 221 published articles were screened, and 188 were assessed for eligibility via full-text evaluation. One hundred and twelve records did not meet the inclusion criteria after this full-text review, and were excluded. As a result, 76 full-text articles were selected for systematic review. Subsequently, 20 full-text articles were selected for meta-analysis, with 6, 18, and 4 articles included for efficacy, immunogenicity, and safety, respectively. The study flow chart is presented in Figure 1.

### 3.1. Study Characteristics

Seventy-six studies were included in the qualitative analysis (Table S2). Ten studies were conducted in Israel [29,30,31,32,33,34,35,36,37,38], one study in Denmark [39], eight studies in Italy [8,40,41,42,43,44,45,46], three studies in the USA [47,48,49], nine studies in Germany [50,51,52,53,54,55,56,57,58], one study in New Zealand [59], three studies in Austria [60,61,62], four studies in Spain [63,64,65,66], one study in Japan [67], one study in France [68], one study in Romania [69], one study in Peru [70], one study in Canada [71], six studies in Brazil [72,73,74,75,76,77], two studies in China [78,79], three studies in Thailand [80,81,82], one study in Chile [83], five studies in India [84,85,86,87,88], one study in Greece [89], one study in Turkey [90], four studies in the UK [91,92,93,94], one study in Korea [95], one study in Taiwan [96], two studies in Netherlands [97,98], one study in Switzerland [85], one study in Hungary [99], and one study in the USA and UK [100]. The types of investigated studies encompassed single-blinded [73,96], observer-blinded randomized [31], and non-randomized [8,29,30,32,33,34,35,36,37,38,39,40,41,42,43,44,45,46,47,48,49,50,51,52,53,54,55,56,57,58,59,60,61,62,63,64,65,66,67,68,69,70,71,72,74,75,76,77,78,79,80,81,82,83,84,85,86,87,88,89,90,91,92,93,94,95,97,98,99,100,101,102,103] studies. A total of 160,447 participants were involved. All studies concerned adult participants (the majority of participants were >18 years of age), and only one study also involved pediatric participants [91]. Sixty-six studies included participants who had only had a primary dose vaccine [8,29,30,35,36,37,38,39,41,43,44,45,46,47,48,49,50,51,52,53,54,55,56,57,58,59,60,61,62,63,64,65,66,67,68,69,70,71,72,73,74,75,76,77,78,79,80,82,83,84,85,86,87,88,89,90,91,92,93,97,98,99,100,101,102,103], whereas in ten studies participants had had a booster dose vaccine [31,32,33,34,40,42,81,94,95,96].

The studies in our qualitative analysis were divided into six categories based on the type of vaccine: studies on mRNA vaccines including Pfizer/BioNTech (BNT162b2) and Moderna (mRNA-1273); studies on inactivated virus vaccines including CoronaVac, Covaxin (BBV152), and Sinopharm (BBIBP-CorV); studies on adenovirus vector vaccines including Vaxzevria (ChAdOx1), Janssen (Ad26.COV2.S), Sputnik V (Gam-COVID-Vac), and AstraZeneca (AZD1222); studies on mRNA vaccines and adenovirus vector vaccines; studies on inactivated virus and adenovirus vector vaccines; and studies on mRNA vaccines, inactivated virus vaccines, and adenovirus vector vaccines.

In terms of autoimmune diagnosis, studies included adult-onset Still’s disease [57,61,89,95], antiphospholipid syndrome [33,47,69,72,74,75,88,89,94,96,102], autoimmune encephalitis [40,52,60], autoimmune hepatitis [44,56,69,90,97,101], autoimmune thyroid [69,96], IgG-4-related diseases [47,69,92,94], interstitial lung disease and systemic autoimmune disease/immune pulmonary disease [49,69], inflammatory bowel disease [47,50,62,69,71,91,97], inflammatory myopathies/systemic autoimmune myopathy [33,35,36,61,63,69,72,74,75,76,77,101], immune-mediated thrombocytopenic purpura/immune-mediated thrombotic thrombocytopenic purpura (ITP/iTTP) [32,42,96], juvenile idiopathic arthritis [57,83,88,89,94,98], mixed/undifferentiated connective tissue disease/connective tissue disease [44,47,50,55,57,61,69,86,87,88,89,94,98,103], multiple sclerosis [29,30,31,34,47,50,52,53,54,60,64,67,69,97,98], myasthenia gravis syndrome [52,60,69,97], neuromyelitis optica spectrum disorder [47,52,60,66,97], primary biliary cholangitis [56,69,97,101], psoriasis [50,69,71,80,97], psoriatic arthritis [33,35,50,57,69,71,72,74,75,83,88,94,98,99], rheumatoid arthritis (RA) [33,35,36,39,43,44,47,50,57,58,63,65,72,73,74,75,78,82,83,84,86,87,88,89,93,94,95,96,97,98,99,101,103], systemic lupus erythematosus (SLE) [33,35,36,39,44,45,47,50,57,61,65,66,68,69,70,72,74,75,77,78,81,82,84,86,87,88,89,92,93,94,95,96,97,98,99,101,102,103], sarcoidosis [50,61,88,94,98], spondiloarthritis/spondyloarthropathy [33,35,44,47,49,50,57,69,71,72,74,75,84,86,87,88,89,94,95,96,97,98,99,101,103], sclerosing cholangitis [56,97], Sjogren syndrome/sicca syndrome [33,47,61,65,69,71,74,75,78,88,89,94,96,98,99], systemic sclerosis [33,44,61,63,65,69,72,74,75,86,87,88,89,94,98,99,101,103], and vasculitides/vasculitis [33,35,36,44,47,50,55,57,58,61,66,69,72,74,75,86,87,88,89,92,93,94,95,97,98,99,103].

Autoimmune medications given to the patients included alemtuzumab [29,34,48,66], abatacept [8,33,35,39,47,57,58,63,72,73,74,75,94,98], anti-CD20/-B cell depleting therapy [8,29,32,33,34,35,37,39,40,41,43,45,47,48,49,52,53,54,55,57,60,62,63,64,65,66,72,74,75,87,89,92,94,96,97,98,103], antimalarials including hydroxychloroquine (HCQ) and chloroquine [8,37,39,41,44,45,47,50,55,61,63,65,68,69,70,72,75,77,81,82,83,84,87,88,89,90,93,94,95,96,98,99,103], apremilast [94,103], azathioprine [8,33,39,40,41,44,45,47,50,52,56,57,58,60,63,65,68,69,70,71,72,74,75,76,80,81,82,84,85,87,88,89,90,92,94,95,98,99,101,103], belimumab [8,39,41,44,45,47,50,57,61,65,68,72,74,75,77,89,92,94,95,99], calcineurin inhibitor [33,41,77,95], caplacizumab [42], certolizumab [50,83,101], cladibrine [29,30,34,48,52,66], colchicine [33,89,94,103], corticosteroids [32,33,35,36,37,39,40,41,42,44,45,47,49,50,52,55,56,57,58,60,62,63,64,68,69,70,72,73,75,76,77,80,81,82,83,84,86,87,88,89,90,91,92,93,94,96,98,99,100,101,103], cyclophosphamide (CYP) [45,62,63,64,72,74,75,76,77,87,89,92,95], cyclosporine (CYC) [45,72,74,75,76,80,81,82,83,85,88,89,94,98], denosumab [94], DMF [29,34,48,52,53,66], eculizumab [94], everolimus [85], fampridine [98], fingolimod [29,30,31,34,47,52,53,64,98], glatiramer acetate (GA) [29,34,48,52,53,64,98], ibrutinib [47,100], iguratimod [88], IL-1 inhibitor [89,94], IL-6 inhibitor [8,33,35,37,39,40,44,45,47,50,57,60,61,63,65,72,74,75,83,85,89,94,95,99,101], IL-17 inhibitor [33,35,44,51,57,71,72,74,75,80,83,89,94,98,99,101], IL-12/23 inhibitor [33,47,71,89,99], IL-23 inhibitor [47,71,99], β interferons [29,34,48,52,54,66,98], intravenous immunoglobulin (IVIG) [29,32,34,36,52,71,100], Janus kinase (JAK) inhibitor [8,33,35,39,57,58,62,88,89,94,95,99,101], leflunomide [8,36,39,45,47,50,58,63,69,70,72,73,74,75,76,77,82,83,84,87,88,89,93,94,95,98,99,103], lenalidomide [87,99], mepolizumab [99], methotrexate [8,33,35,36,39,41,43,44,47,57,58,61,63,65,68,69,70,71,72,73,74,75,76,77,80,82,83,84,87,88,89,90,92,93,94,95,96,97,98,99,101,103], mycophenolate mofetil [8,33,35,36,37,39,40,41,43,44,45,47,49,56,57,60,61,63,65,68,69,70,72,74,75,76,77,80,81,82,83,84,85,87,88,89,90,92,94,95,97,100,101,103], natalizumab [29,34,48,52,53,66,98], nintedanib [49], ocrelizumab (OCR) [29,30,34,48,52,60], ofatumumab [48], olumiant [61], omalizumab [80], pembrolizumab [99], plasmapheresis (PLEX) [42,52,64], sphingosine-1-phosphate receptor modulators (S1PRM) [48,66,97], salazopyrin [39], sulfasalazine [8,44,47,51,58,63,69,72,75,83,84,87,93,94,95,96,97,98,98,103], tacrolimus [45,61,72,74,75,81,82,83,85,87,92,94,103], teriflunomide [29,34,48,52,53,66], thalidomide [89,94], tumor necrosis factor alpha inhibitor (TNFi) [8,33,35,39,44,45,47,50,51,57,58,62,63,65,71,72,74,83,87,89,91,94,95,97,98,99,101,103], tofacitinib [37,47,72,74,75,87,103], upadacitinib [37,47], ustekinumab [50,72,74,75,98], and vedolizumab [47,50,91,92].

### 3.2. Quality of Assessment

Graphical representation of the studies’ quality is illustrated in the Supplementary Materials (Figure S1A–D). Risks of bias in the three RCTs were low; twenty-one non-randomized studies were low-risk, thirty-seven non-randomized studies were moderate-risk, and seven non-randomized studies had serious risk; four case-series studies were defined as good; and four cross-sectional studies were considered fair.

### 3.3. Qualitative Analysis

#### 3.3.1. Efficacy

In the mRNA vaccine studies group, efficacy after primary vaccination was reported as breakthrough COVID-19 infections [29,35,41,49,63,101], hospitalizations [49,63], and deaths [29,35,49,63]. Efficacy after booster vaccination was also reported as breakthrough COVID-19 infections [33,34] and hospitalizations and deaths [33]. In the inactivated virus vaccine studies, six studies reported breakthrough COVID-19 infections after primary vaccination as outcomes [72,75,76,77,80,83], two studies reported hospitalizations [76,77], and only one study reported death [77]. One study on adenovirus vector vaccines reported breakthrough infections [87]. In the mRNA vaccine and adenovirus vector vaccine studies, efficacy after primary vaccination was reported as breakthrough COVID-19 infections [45,69,97,98], hospitalizations [95,97], and deaths [100]. Efficacy after booster vaccination was reported as breakthrough COVID-19 infections, hospitalizations, deaths [89], and hospitalizations or deaths due to breakthrough infections [90]. In the inactivated virus vaccine and adenovirus vaccine studies, one study reported breakthrough COVID-19 infections after primary vaccination [103]. In the mRNA vaccine, inactivated virus vaccine, and adenovirus vector vaccine studies, efficacy was reported as breakthrough COVID-19 infections and hospitalizations after primary vaccination [88,94]. Breakthrough COVID-19 infections and deaths after booster vaccination were reported in only one study [94].

mRNA vaccination, either primary or booster, has been found to have a protective effect on breakthrough infections where the risk of getting infections after vaccination is lower compared with the unvaccinated group [33]. According to Bieber et al., patients with autoimmune rheumatic disease who received a third booster of mRNA vaccination had lower SARS-CoV-2 infection rates [33]. However, Kim et al. observed both patients and healthy controls to have SARS-CoV-2 omicron breakthrough infections after a third dose of vaccination [95]. Mena-Vázquez et al. also reported that patients who were not infected with SARS-CoV-2 received vaccinations more frequently. Moreover, COVID-19-infected patients took rituximab and glucocorticoids more frequently [63]. 

Symptomatic breakthrough COVID-19 infections among patients and in a healthy control group were reported in two studies after the participants had had a primary inactivated COVID-19 vaccination [76,77], although only one patient required hospitalization and no patients died [77]. Non-severe infections were reported after a mean period of fourteen weeks from full vaccination, where half of the infected participants were patients with negative total anti-SARS-CoV-2 IgG antibodies and neutralizing antibodies [83].

Studies in which autoimmune patients received an mRNA or adenovirus vector vaccine reported a higher hospitalization rate in the unvaccinated group compared with the vaccinated group, as well as a higher rate of severe COVID-19 cases, which appeared less frequently in third-dose-vaccinated patients than in second-dose-vaccinated patients and an unvaccinated group [89]. Breakthrough infections were also more frequent in patients on strongly impairing immunosuppressants, including anti-CD20 combination therapy, sphingosine 1-phosphate modulators, and mycophenolate mofetil therapy, as opposed to patients on other immunosuppressants [97]. 

According to the results from a study on inactivated and adenovirus vaccines, the strongest predictor of breakthrough infections is the absence of an antibody response. Vaccine platform and mycophenolate mofetil were found to be the other breakthrough infection predictors [103]. Patients with autoimmune disease receiving Covaxin showed higher rates of breakthrough infection than those receiving the AstraZeneca vaccine [103]. Another result from a study on adenovirus vector vaccines reported that there was no significant difference in the frequency of breakthrough infections between patients who received a second dose of vaccine after 4–6 weeks versus 10–14 weeks [87]. Furthermore, results from a study reporting on autoimmune patients given mRNA, inactivated virus, or adenovirus vector vaccines showed no breakthrough infections in patients vaccinated with mRNA. Meanwhile, inactivated-virus-vaccinated patients had a higher percentage of breakthrough infections after full vaccination than adenovirus-vector-vaccinated patients, although the difference was not significant [88].

#### 3.3.2. Immunogenicity

There were 54 studies reporting immunogenicity: 27 studies on mRNA vaccines (13 studies on Pfizer/BioNTech [30,31,34,36,38,39,43,46,54,59,61,67,68], 1 study on Moderna [101], and 13 studies on Pfizer/BioNTech or Moderna [8,35,41,44,47,50,51,52,53,60,62,65,71]); 9 studies on inactivated virus vaccines using CoronaVac [72,73,74,75,76,77,79,80,83]; 2 studies on adenovirus vector vaccines [84,87]; 12 studies on mRNA and adenovirus vector vaccines (5 studies on Pfizer/BioNTech, Moderna, Vaxzevria, or Janssen [55,57,66,95,98], 2 studies on Pfizer/BioNTech, Moderna, or Vaxzevria [56,58], 3 studies on Pfizer/BioNTech or Vaxzevria [91,92,93], 1 study on Moderna or Vaxzevria [96], and 1 study on Pfizer/BioNTech (BNT162b2), Moderna (mRNA-1273), or Janssen (Ad26.COV2.S)) [85]; 1 study on inactivated virus vaccines and adenovirus vector vaccines using Covaxin or AstraZeneca [103]; 3 studies on mRNA vaccines, inactivated vaccines, and adenovirus vector vaccines using Pfizer, Coronavac, or Vaxzevria [81], Pfizer, Coronavac, Sinopharm or Vaxzevria [82], and Pfizer, Moderna, Sinopharm, Sputnik, and AstraZeneca [99]. Immunogenicity was determined by measuring antibody titers [8,30,31,34,35,36,38,39,41,43,46,47,50,51,52,53,54,56,57,58,59,60,61,62,65,66,67,68,75,76,80,81,82,83,84,85,87,92,93,96,98,99,101,103], seroconversion [8,30,31,35,36,39,41,43,44,46,47,52,53,54,55,56,57,60,61,65,66,71,72,73,74,75,76,77,80,82,84,92,95,97,98,99,101], neutralization antibodies [38,41,47,50,51,53,54,58,62,68,72,73,74,75,76,77,80,81,83,84,95,99,101,103], T-cell response [41,43,46,53,54,55,56,57,60,62,65,66,68,71,81,82,83,91,95,99,101], lymphocyte count [31,93], IgA titer [38,50,58,85,93], IgG avidity [51,54], B-cell counts [43,49,53,56,57,58,93], T-cell counts [55,58,62,93], and IgM titer [93].

Patients with autoimmune diseases who received CoronaVac had neutralizing antibodies and neutralizing activity lower than in the control group [72,74,75] as well as lower seroconversion [74,75]. Factors associated with poor immunogenicity were older age, obesity, and use of prednisone, biologics, and immunosuppressants [74,75]. Another study on patients given CoronaVac also found that mycophenolate and prednisone were related to reduced seroconversion, whereas hydroxychloroquine caused seroconversion to rise [77]. In another study on Pfizer, CoronaVac, Sinopharm, and Vaxzevria vaccination, anti-RBD titers were lower in the inactivated vaccine group, followed by Vaxzevria, then Vaxzevria or Pfizer [82]. The inactivated vaccine was also associated with the lowest humoral response, whereas the adenovirus-vectored/mRNA vaccine was associated with the highest humoral response [82].

Patients with multiple sclerosis who received the Pfizer vaccine while being treated with anti-CD20 therapy [54], fingolimod continuation [31], and other immunosuppressants [34] had lower IgG titers compared with untreated patients or patients who discontinued the therapy. In comparison with healthy controls, patients with autoimmune neurological disorder who had received the Pfizer or Moderna vaccines had decreased seroconversion rates [34] and anti-S1 IgG [53,60] and anti-S(RBD) specific IgG levels [52]. In comparison with healthy controls or patients not receiving immunotherapy, patients receiving anti-CD20 [52,53,60], fingolimod [52,53], azathioprine [52], and steroid therapy [52] exhibited lower levels of anti-S1 IgG and anti-S(RBD) specific IgG. Lower seroconversion rates were observed in multiple sclerosis patients receiving anti-CD20 or sphingosine 1-phosphate receptor modulators who were given the Pfizer, Moderna, or AstraZeneca vaccines compared with other disease-modifying therapies or untreated patients [66]. 

Additional research on the Pfizer or Vaxzevria vaccine indicated that seroconversion and anti-S IgG levels after the second dose were significantly lower in patients with autoimmune disease than in the control group and that this was associated with B-cell depletion at the time of vaccination [92]. Rituximab was significantly associated with no antibody vaccine response after adjusting for diagnosis and hydroxychloroquine, according to research in patients with SLE and RA who received the Pfizer vaccine [39]. A study of patients with SLE who had been given the Pfizer vaccination found that mycophenolate and methotrexate treatment were associated with a drastically diminished BNT162b2 antibody response [68]. Another study on the Moderna and Vaxzevria vaccines showed that individuals given hydroxychloroquine, low-dose steroid, methotrexate, and/or sulfasalazine therapy had significantly lower anti-SARS-CoV-2 spike IgG titers than those who were not on these therapies [96]. 

Studies of patients with autoimmune or autoinflammatory diseases, including RA, SLE, Sjogren syndrome, Behcet’s disease, polymyalgia rheumatica, connective tissue disease, vasculitis, adult-onset Still’s disease, and sarcoidosis who received the Pfizer vaccination showed lower seroconversion [46,61], anti S1/S2 IgG [36,38,43,61], neutralization [38,43], total IgA [38,43], and anti-RBD IgG [61] than the control group. The lowest antibody titers were detected in patients with antineutrophil cytoplasmic-antibody-associated vasculitis (AAV) and idiopathic inflammatory myopathy/myositis (IIM), while the highest titers were detected in SLE and RA patients [37]. Another study showed that antibody titers were also reduced with two or more immunosuppressants in combination therapy [61]. Studies in patients with systemic autoimmune disease, RA, SLE, inflammatory bowel disease, Sjogren syndrome, autoimmune hepatitis, psoriatic arthritis, IIM, sarcoidosis, and vasculitis who received the Pfizer and Moderna vaccines found that their anti-S IgG titers were lower than those of the control group, and these differences were particularly significant [8,35,50,51] in those who were receiving B-cell-depleting therapies, prednisone, JAK inhibitors, antimetabolites [47], TNFi [51], mycophenolate, and calcineurin inhibitors [44]. Moreover, compared with the control group, anti-RBD titers were lower in patients [41]. The differences remained significant in individuals receiving treatment with rituximab and belimumab [41]. According to another study, Ab levels and neutralization efficacy against variants of concern in anti-TNF-treated patients were substantially lower than in healthy controls, and by three months following the second dose of the vaccination they were undetectable against Omicron [71]. 

Seroconversion was considerably higher among Pfizer vaccine recipients when doses were given less than a month apart compared with AstraZeneca recipients, and tendencies towards higher antibody levels in vaccine responders were seen when either vaccine was given using short-interval dosing [93]. A study by Mehta et al. on the AstraZeneca vaccine showed that diabetes mellitus and vaccine interval were significantly associated with anti-RBD antibody titer [87]. A delayed (10–14 weeks) second dose of AstraZeneca vaccine was associated with a higher antibody titer [87]. A study by Ahmed et al. on AstraZeneca and Covaxin revealed that Covaxin and methotrexate treatment were associated with lower antibody titers [103]. Another study that focused on Vaxzevria vaccination in patients with autoimmune inflammatory rheumatic diseases revealed that single-dose-vaccinated patients who had had prior COVID-19 infections showed significantly higher seroconversion and neutralization activity than those who had received a double-dose vaccine [84].

In a study that focused on CoronaVac vaccination, neutralizing antibodies in RA patients on methotrexate therapy were lower than in the control group [73,83], as was the seroconversion rate [73]. Prednisone and mycophenolate usage were both highly linked to a negative NAb [83].

A study on mRNA and inactivated virus vaccines reported that IFN-γ and anti-RBD Abs levels have a slight but significant positive correlation [43]. Another study on mRNA, inactivated virus, and adenovirus vector vaccines reported that neutralizing anti-RBD-specific antibodies and the percentage of positive anti-RBD antibody responses were higher in participants vaccinated with mRNA vaccine compared with inactivated virus and adenovirus vaccines [99]. Additionally, patients who received the adenovirus vector or mRNA vaccines had a higher proportion of TNF-a-producing CD4+ T-cells upon SARS-CoV-2 antigen exposure compared with those who received the inactivated virus vaccine [99].

A third booster dose of mRNA or adenovirus vector vaccine after a primary inactivated vaccine produced a significant humoral and cellular immune response in SLE patients with inactive disease maintaining immunosuppressive treatment [81]. However, another study found that, after booster vaccination, neutralization responses against the Omicron variant were significantly lower in patients than in the healthy control group [92]. Certain medications, such as TNFi, aCD20-BCD- and fingolimod, antimetabolites, and calcineurin inhibitors were able to impair humoral and cellular responses, especially in autoimmune patients [51,53,81]. For instance, Achiron et al. found that a fingolimod continuation group had lower IgG titers than a fingolimod discontinuation group even at 3 months after the third vaccine dose [31]. In addition, anti-BA.2 neutralizing antibodies were not detectable in TNFi-treated patients [51]. Meyer et al. found that patients taking fingolimod failed to develop either humoral or CD4^+^ T cellular immune responses [53]. In contrast, Meyer et al. also reported that untreated patients showed an increase in anti-S1 IgG, neutralizing capacity, RBD- and S2-specific B cells, and spike-specific T cells after their first booster [53]. Lastly, however, a booster dose, particularly from an mRNA or viral vector vaccine, enhanced strong cellular immune responses, though responses were weaker in patients taking antimetabolites or calcineurin inhibitors [81].

#### 3.3.3. Safety

Following primary vaccination in mRNA vaccine studies, autoimmune relapse was reported as a safety outcome in 12 studies [29,40,60,63,68,70,80,81,82,86,88,99]; local symptoms in 11 studies [8,29,35,36,37,40,50,52,60,67,68]; systemic symptoms in 13 studies [8,34,35,36,37,40,50,52,60,63,67,68,101]; and other symptoms in 12 studies [29,35,36,40,50,52,60,64,88,100,101,102]. Following booster vaccination in mRNA vaccine studies, autoimmune relapse was reported as a safety outcome in three studies [32,34,42] and local and systemic symptoms were also reported in one study [34]. Among the inactivated vaccine studies, following primary vaccination, autoimmune relapse was reported as a safety outcome in two studies [79,80] and local symptoms and systemic symptoms in six studies [72,73,75,76,77,78].

Among the mRNA vaccine and adenovirus vector vaccine studies, following primary vaccination, two studies reported autoimmune relapse after vaccination [20,69]. Local symptoms were reported as a safety outcome in three studies [45,69,72]. Systemic symptoms after primary mRNA and adenovirus vector vaccinations were reported in three studies [45,48,102]. Other symptoms were described in one study [82]. Meanwhile, among the inactivated virus vaccine and adenovirus vector vaccine studies, only one study reported autoimmune relapses and local and systemic symptoms as safety outcomes [86]. Among the mRNA vaccine, inactivated virus vaccine, and adenovirus vector vaccine studies, following primary vaccination, autoimmune relapse was reported as a safety outcome in two studies [88,94]; local symptoms in three studies [88,94]; systemic symptoms in four studies [82,88,94,99]; and other adverse events in two studies [88,94].

Patients who had been vaccinated with an mRNA vaccine reported no difference in relapse incidence before and after vaccination [8,52,68]. De Santis et al. and Ferri et al. also reported that, in the majority of cases, vaccine-related adverse effects were mild, and incidence rates were comparable in autoimmune patients and healthy controls with no differences based on current medications [8,101]. Mild cases, such as headache, occurred more frequently in SLE and cryoglobulinemic vasculitis patients, while pain at the injection site did in systemic vasculitis patients [8]. Moyon et al. found no related serious adverse events caused by vaccination [68]. Most of the relapse cases had significantly higher disease activity scores when compared with patients without post-vaccination relapses [40]. Additionally, De Santis et al. did not find any differences between patients with and without serum responses or in the prevalence of vaccine-related side effects [101]. In terms of booster vaccinations, a study reported more than 10% ITP exacerbations among ITP patients after booster vaccinations [32].

Patients who had been vaccinated with an inactivated virus vaccine were reported to have no moderate or severe adverse events [76,77]. Medeiros-Ribeiro et al. reported that overall reactions, such as arthralgia, back pain, malaise, nausea, and sweating, were more frequently and significantly found to occur in patients with autoimmune rheumatic disease than in a control group [75]. In patients with RA, myalgia and vertigo were significantly more frequent in those patients who were stopping methotrexate therapy at the time of receiving their second vaccination [73]. Headaches had a higher prevalence in patients with systemic autoimmune myopathies compared with healthy controls after a first dose of inactivated vaccine [76]. Autoimmune flare was also detected more frequently in a methotrexate-stopping RA patient group in comparison with a methotrexate-maintaining group at day 69 after vaccine administration [73].

Studies on mRNA and adenovirus vector vaccines reported that there was no difference in self-reported side effects between patients with neuroinflammatory diseases and a control group, whether after first vaccine dose or second vaccine dose, even after adjusting for age, BMI, and comorbidities [48]. Epstein et al. also reported that younger age was associated with an increased rate of reported side effects, whereas patients on high-efficacy therapy were associated with a lower risk of reported side effects [48]. The high-efficacy therapies referred to were therapies using ocrelizumab, rituximab, ofatumumab, alemtuzumab, cladribine, fingolimod, ozanimod, siponimod, and natalizumab [48]. Headaches were more common in patients with neuroinflammatory disease after mRNA vaccination than adenovirus vector vaccination, although no significant differences were observed [48]. Additionally, patients on high-efficacy therapy had a significantly lower rate of reported side effects compared with patients not on medication at the time of vaccination [48]. In terms of flare, there were no differences observed regarding age, comorbidities, number of autoimmune diseases associated, and years from disease diagnosis to the year prior to vaccination [69]. There were no significant differences in flare-up development among Cominarty, Vaxzevria, and Spikevax [69]. 

Additionally, studies on mRNA, inactivated virus, and adenovirus vector vaccines reported significantly more injection site pain in patients receiving AstraZeneca or Pfizer vaccination than in those who received inactivated vaccination, followed by fatigue and fever [82]. Another study on a third booster dose with an mRNA or viral vector vaccine following inactivated virus vaccination in SLE patients revealed more reactogenicity after the booster dose than the initial CoronaVac vaccination, but this was mild and no SLE flare was reported [81].

### 3.4. Meta-Analysis

For meta-analysis, we included 20 studies that compared the efficacy, immunogenicity, and safety of COVID-19 vaccines between patients with autoimmune diseases and healthy controls. There were six studies for efficacy [72,75,76,77,97,98], 18 studies for immunogenicity [8,35,36,43,50,52,54,61,62,65,72,74,75,76,77,80,83,101], and four studies for safety that could be included [72,75,76,77]. These studies were on inactivated vaccine, mRNA vaccine, and mRNA/adenovirus vector vaccine. All studies were non-randomized studies and on primary doses (two doses) of COVID-19 vaccine. Meta-analysis could not be done from the RCTs because there were only three RCTs [31,73,96] in our systematic review and only one RCT comparing the efficacy, immunogenicity or safety of the COVID-19 vaccine (primary dose) among patients with autoimmune disease (multiple sclerosis) and healthy controls [31]. 

#### 3.4.1. Efficacy

Six studies were included to evaluate the efficacy of COVID-19 vaccines in patients with autoimmune diseases. Four studies used the inactivated virus vaccine [72,75,76,77], whereas the other two studies used mRNA and adenovirus vector vaccines [97,98] Breakthrough COVID-19 infections were used to assess vaccine efficacy. 

**Figure 2:**
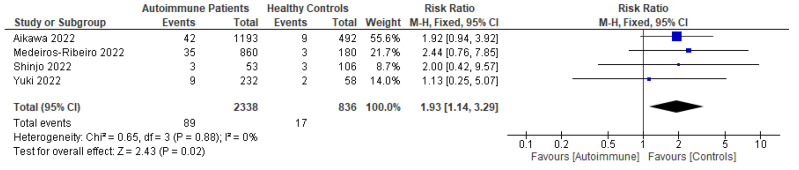
Breakthrough COVID-19 infections after receiving primary doses (two doses) of COVID-19 inactivated vaccine. Blue squares represent effect sizes for a single study, and black rhombus represent pooled results for all studies [72,75,76,77].

Based on Figure 2, the overall effect on breakthrough COVID-19 infection after receipt of a COVID-19 inactivated virus vaccine was in favor of the healthy controls. The combined risk ratio was 1.93 (95% CI: 1.14–3.29, I^2^ = 0%), and the difference was statistically significant (*p* = 0.02). According to the GRADE system, the certainty of the evidence on breakthrough COVID-19 infections after inactivated vaccination was moderate (Supplementary Materials, Table S3). Four studies included in this meta-analysis involved patients with various autoimmune diseases: SLE, systemic autoimmune myopathies, and other autoimmune diseases. Patients involved in these four studies received various immunosuppressive treatments: steroids, methotrexate, hydroxychloroquine, mycophenolate mofetil, azathioprine, biologic agents, and others. 

**Figure 3:**
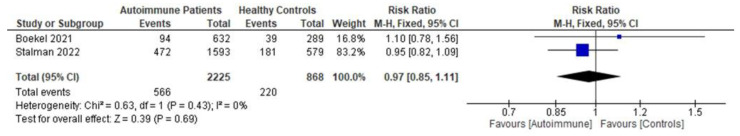
Breakthrough COVID-19 infections from studies using mRNA and adenovirus viral vector COVID 19 vaccines. Blue squares represent effect sizes for a single study, and black rhombus represent pooled results for all studies [97,98].

We also analyzed the combined risk ratio for breakthrough infections after mRNA or adenovirus vector vaccination, but no statistically significant difference was observed (RR = 0.97; 95% CI: 0.85–1.11; I^2^ = 0%) (Figure 3). According to the GRADE system, the certainty of the evidence on breakthrough COVID-19 infections after mRNA or adenovirus vector vaccination was moderate (Supplementary Materials). Three studies included in this meta-analysis involved patients with various autoimmune diseases: SLE, rheumatoid arthritis, spondiloarthopathy, vasculitis, and others. Patients involved in these four studies received various immunosuppressive treatments: steroids, methotrexate, hydroxychloroquine, leflunomide, mycophenolate mofetil, azathioprine, biologic agents, and others. Subgroup analysis regarding autoimmune diagnosis and treatment could not be done because of limited studies or a lack of subgroup data.

#### 3.4.2. Immunogenicity

Eighteen studies were included in the meta-analysis to evaluate the immunogenicity of COVID-19 vaccines in patients with autoimmune disease compared with healthy controls [8,35,36,43,50,52,54,61,62,65,72,74,75,76,77,80,83,101]. Studies included in this meta-analysis involved patients with various autoimmune diseases: multiple sclerosis, systemic autoimmune diseases, and other autoimmune diseases. Patients involved in these studies received various immunosuppressive treatments: steroids, methotrexate, hydroxychloroquine, mycophenolate mofetil, azathioprine, biologic agents, and others.

Eleven studies were on mRNA vaccines [8,35,36,43,50,52,54,61,62,65,101] and seven studies [72,74,75,76,77,80,83] on inactivated vaccines. Seroconversion, proportion of neutralizing antibodies (NAb) positive, log total antibody (TAb) titer, and neutralizing activity were analyzed. 

**Figure 4:**
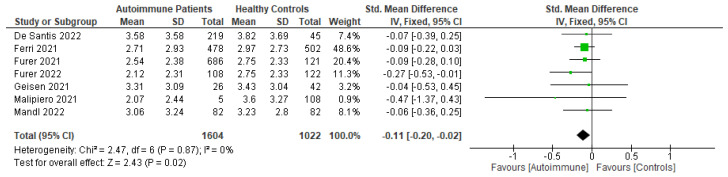
Log TAb titer after mRNA vaccination. Green squares represent effect sizes for a single study, and black rhombus represent pooled results for all studies [8,35,36,43,50,61,101].

As shown in Figure 4, seven studies reported TAb titers after mRNA vaccination. Patients with autoimmune disease showed significantly lower log TAb (log BAU/mL) titers than healthy controls. Heterogeneity was low (SMD = −0.11, 95% CI = −0.2–0.02, I^2^ = 0%). According to the GRADE system, the certainty of the evidence on TAb after mRNA vaccination was high (Supplementary Materials, Table S3).

**Figure 5:**
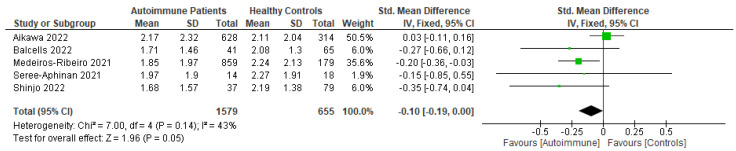
Log TAb titer after inactivated vaccination. Green squares represent effect sizes for a single study, and black rhombus represent pooled results for all studies [72,75,76,80,83].

As shown in Figure 5, five studies reported Tab titers after inactivated vaccination. Patients with autoimmune disease showed significantly lower log Tab (log BAU/mL) titers compared with healthy controls. Heterogeneity was considerably low (SMD = −0.10, 95% CI = −0.19–0.00, I^2^ = 43%). According to the GRADE system, the certainty of the evidence on TAb titer after inactivated vaccination was high (Supplementary Materials, Table S3).

**Figure 6:**
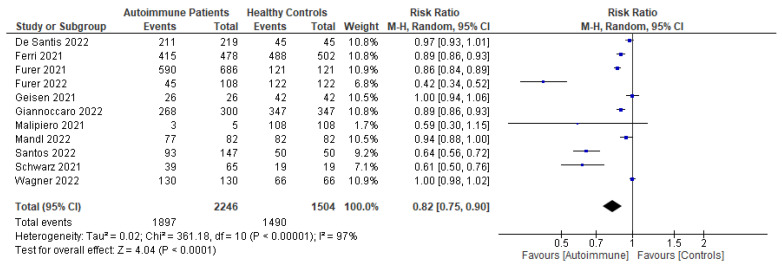
IgG seroconversion after mRNA vaccination. Blue squares represent effect sizes for a single study, and black rhombus represent pooled results for all studies [8,35,36,43,50,52,54,61,62,65,101].

As shown in Figure 6, 11 studies reported IgG seroconversion after mRNA vaccination compared with healthy controls. IgG Seroconversion after mRNA vaccination was significantly lower among patients with autoimmune disease than healthy controls. Heterogeneity was high (RR = 0.82, 95% CI = 0.75–0.90, I^2^ = 97%). According to the GRADE system, the certainty of the evidence on IgG seroconversion after mRNA vaccination was moderate (Supplementary Materials).

**Figure 7:**
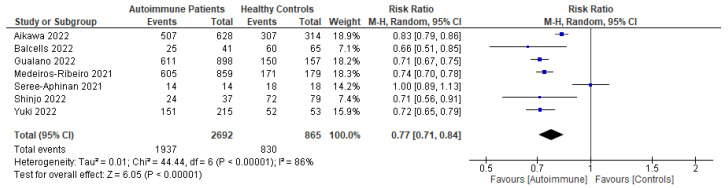
IgG seroconversion after inactivated vaccination. Blue squares represent effect sizes for a single study, and black rhombus represent pooled results for all studies [72,74,75,76,77,80,83].

As shown in Figure 7, seven studies reported IgG seroconversion after inactivated vaccination compared with healthy controls. IgG seroconversion after inactivated vaccination was significantly lower among patients with autoimmune disease than healthy controls. Heterogeneity was considerably high (RR = 0.77, 95% CI = 0.71–0.84, I^2^ = 86%). According to the GRADE system, the certainty of the evidence on IgG seroconversion after mRNA vaccination was moderate (Supplementary Materials, Table S3).

**Figure 8:**
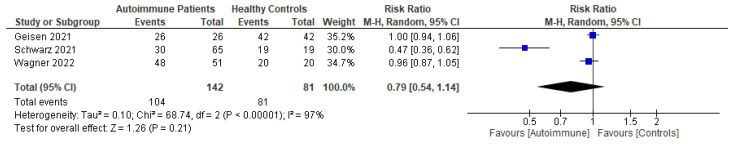
Proportion of neutralizing antibodies positive after mRNA vaccination. Blue squares represent effect sizes for a single study, and black rhombus represent pooled results for all studies [50,54,62].

As shown in Figure 8, three studies reported neutralizing antibodies after mRNA vaccination. Patients with autoimmune disease showed a lower proportion of positive NAb than healthy controls, but the difference was not statistically significant. Heterogeneity was high (RR = 0.79, 95% CI = 0.54–1.14, I^2^ = 97%). According to the GRADE system, the certainty of the evidence on neutralizing antibodies after mRNA vaccination was very low (Supplementary Materials, Table S3).

**Figure 9:**
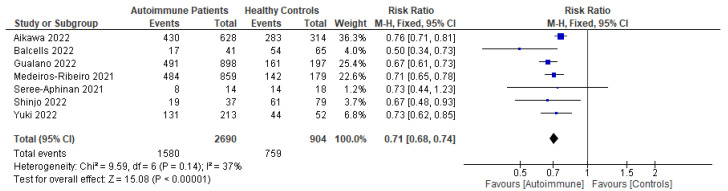
Proportion of neutralizing antibodies positive after inactivated vaccination. Blue squares represent effect sizes for a single study, and black rhombus represent pooled results for all studies [72,74,75,76,77,80,83].

As shown in Figure 9, seven studies reported neutralizing antibodies after inactivated vaccination. Patients with autoimmune disease had a significantly lower proportion of positive NAb than healthy controls. Heterogeneity was considerably low (RR = 0.71, 95% CI = 0.68–0.74, I^2^ = 37%). According to the GRADE system, the certainty of the evidence on neutralizing antibodies after inactivated vaccination was high (Supplementary Materials, Table S3).

**Figure 10:**
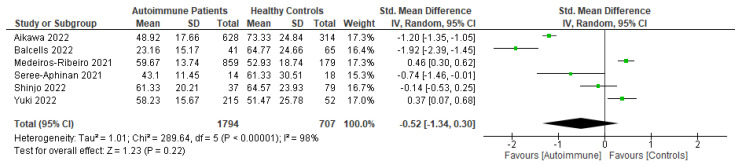
Neutralizing activity after inactivated vaccination. Green squares represent effect sizes for a single study, and black rhombus represent pooled results for all studies [72,75,76,77,80,83].

As shown in Figure 10, six studies reported neutralizing activity after inactivated vaccination. Patients with autoimmune disease showed lower mean neutralizing activity after inactivated vaccination than healthy controls, but the result was not statistically significant. Heterogeneity was high (SMD = −0.52, 95% CI = −1.34–0.30, I^2^ = 98%). According to the GRADE system, the certainty of the evidence on neutralizing antibodies after the first dose of vaccine was very low (Supplementary Materials, Table S3).

#### 3.4.3. Safety

Four studies were eligible for pooling of vaccine-associated adverse events, including local and systemic adverse events. All included studies were on inactivated COVID-19 vaccines [72,75,76,77]. Four studies included in this meta-analysis involved patients with various autoimmune diseases: SLE, systemic autoimmune myopathies, and other autoimmune diseases. Patients involved in these four studies received various immunosuppressive treatments: steroids, methotrexate, hydroxychloroquine, mycophenolate mofetil, azathioprine, biologic agents, and others.

**Figure 11:**
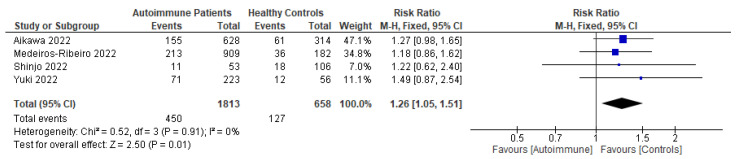
Local adverse events after receiving a first dose of COVID-19 inactivated vaccine. Blue squares represent effect sizes for a single study, and black rhombus represent pooled results for all studies [72,75,76,77].

We observed that the combined risk ratio for local adverse events after a first dose of COVID-19 inactivated vaccine was 1.26 (95% CI: 1.05–1.51; I^2^ = 0%) (Figure 11). Patients with autoimmune diseases had a statistically significant (*p* = 0.01) risk of local adverse events after receiving a first dose of COVID-19 inactivated vaccine in comparison with healthy controls. According to the GRADE system, the certainty of the evidence on local adverse events after first dose COVID-19 inactivated vaccine was high (Supplementary Materials, Table S3).

**Figure 12:**
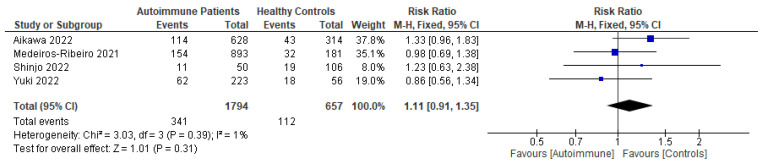
Local adverse events after receiving a second dose of COVID-19 inactivated vaccine. Blue squares represent effect sizes for a single study, and black rhombus represent pooled results for all studies [72,75,76,77].

We observed that the combined risk ratio for local adverse events after a second dose COVID-19 inactivated vaccine was 1.11 (95% CI: 0.91–1.35; I^2^ = 1%) (Figure 12). Patients with autoimmune diseases had a higher risk of local adverse events than healthy controls after receiving a second dose of COVID-19 inactivated vaccine, but the difference was not statistically significant (*p* = 0.31). According to the GRADE system, the certainty of the evidence for local adverse events after a second dose of COVID-19 inactivated vaccine was high (Supplementary Materials, Table S3).

**Figure 13:**
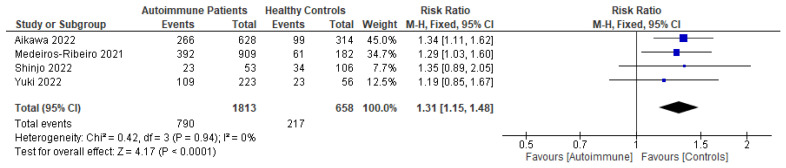
Systemic adverse events after receiving a first dose of COVID-19 inactivated vaccine. Blue squares represent effect sizes for a single study, and black rhombus represent pooled results for all studies [72,75,76,77].

We observed that the combined risk ratio for systemic adverse events after a first dose of COVID-19 inactivated vaccine was 1.31 (95% CI: 1.15–1.48; I^2^ = 0%). Patients with autoimmune diseases had a statistically significant (*p* < 0.0001) risk of systemic adverse events after receiving a first dose of COVID-19 inactivated vaccine in comparison with healthy controls (Figure 13). According to the GRADE system, the certainty of the evidence on systemic adverse events after a first dose of COVID-19 inactivated vaccine was high (Supplementary Materials, Table S3).

**Figure 14:**
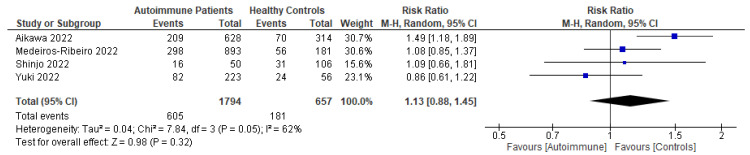
Systemic adverse events after receiving a second dose of COVID-19 inactivated vaccine. Blue squares represent effect sizes for a single study, and black rhombus represent pooled results for all studies [72,75,76,77].

The combined risk ratio for systemic adverse events after a second dose of COVID-19 inactivated vaccine was 1.13 (Figure 14), but no statistically significant difference was observed (95% CI: 0.88–1.45; I^2^ = 62%). According to the GRADE system, the certainty of the evidence on local adverse events was moderate (Supplementary Materials, Table S3).

### 3.5. Publication Bias

We used a funnel plot to assess publication bias for a meta-analysis involving more than 10 studies: IgG seroconversion after mRNA vaccination (Supplementary Materials, Figure S2). The funnel plot was asymmetrical, which could indicate that there was publication bias.

## 4. Discussion

There are some issues regarding COVID-19 vaccination in autoimmune patients, such as how autoimmune medications might affect the efficacy and immunogenicity of the vaccines and possible adverse reactions following COVID-19 vaccination. Therefore, the efficacy, immunogenicity, and safety of COVID-19 vaccines in autoimmune patients were the primary outcomes in this systematic review and meta-analysis.

Only a few studies were identified that addressed all three outcomes. In the meta-analysis, we compared efficacy, immunogenicity, and safety between patients with autoimmune diseases and healthy controls. Because of the heterogeneity of the studies, we only had non-randomized studies that could be used for this purpose. We also could not conduct a meta-analysis on booster (third-dose) COVID-19 vaccination due to limited studies sharing similar outcomes and interventions. 

Regarding the efficacy of COVID-19 vaccination, our meta-analysis showed that the risk of breakthrough COVID-19 infection significantly increased in patients with autoimmune diseases compared with healthy controls after receipt of an inactivated virus vaccine. On the other hand, a meta-analysis with studies using mRNA or adenovirus vectors did not show significant differences in breakthrough infections among patients with autoimmune disease compared with healthy controls. Breakthrough COVID-19 infection can be related to viral profile, host factors (comorbidities, immunosuppressive drugs), and vaccine platform or dose. The mRNA vaccine platform shows stronger neutralizing antibody and T cell responses compared with other vaccine platforms [104].

Ahmed et al. reported that only small numbers of breakthrough infections occurred in patients with autoimmune diseases after they received either an inactivated or adenovirus vector vaccine [103]. Furer et al. observed no symptomatic COVID-19 infections in patients with autoimmune diseases, and only one subject in the healthy control group was diagnosed with a breakthrough COVID-19 infection after a second dose of mRNA vaccine during the study follow-up [35]. Moreover, Stalman et al. reported breakthrough COVID-19 infections after mRNA or adenovirus vector vaccine in both autoimmune patients and healthy controls, with no differences in the trends in the incidence rates [97]. Kim et al. also reported breakthrough infections after booster vaccination with an mRNA vaccine in subjects given an mRNA or adenovirus vector vaccine as their primary COVID-19 vaccination, but the result was not significantly different between patients with autoimmune disease and healthy controls (healthcare workers) [95]. 

Studies included in a meta-analysis of breakthrough infections after mRNA or adenovirus vector vaccination involved patients with various diagnoses and treatments for autoimmune diseases. Patel et al. and Paik et al. explained that increased breakthrough infections were associated with the use of multiple immunomodulatory therapies, such as methotrexate, mycophenolate mofetil, anti-CD20, and TNF inhibitors [105,106]. A study by Bieber et al. also showed higher doses of steroids and higher proportions of patients given TNF alpha inhibitors, rituximab, and calcineurin inhibitors among cases of breakthrough COVID-19 infection [33]. 

Regarding the immunogenicity of the vaccine, our meta-analyses showed that patients with autoimmune diseases had reduced total antibody (TAb) titers, IgG seroconversion, and neutralizing antibodies after COVID-19 inactivated vaccination compared with healthy controls. Patients with autoimmune diseases also showed reduced TAb titers and IgG seroconversion after COVID-19 mRNA vaccination compared with healthy controls. A study by Kim et al. on mRNA vaccine boosters showed that limited neutralization of the Omicron variant in the sera of patients with autoimmune disease could contribute to a shorter median time between third-dose vaccination and the time of breakthrough infection compared with a control group [95].

Patients with autoimmune diseases showed noticeably different humoral responses following vaccination, which may be attributed to the use of B-cell-depleting agents, antimetabolites, glucocorticoids, other immunosuppressive drugs, and waning immunity [106]. This was proven by Ferri et al. in a study that showed an increased prevalence of non-responders to vaccines in patients with systemic autoimmune disease treated with glucocorticoids, mycophenolate mofetil, and rituximab [8]. So et al. found that impaired humoral response in SLE patients significantly correlated with the use of mycophenolate and the type of vaccine, especially inactivated virus vaccines in comparison with mRNA vaccines [107]. Paik et al. reported that B-cell-depleting agents, antimetabolites, glucocorticoids, and combination immunosuppressive therapy achieved significantly lower seroconversion, while immunomodulators, such as hydroxychloroquine and intravenous globulin, did not reduce antibody titers [106]. However, patients treated with hydroxychloroquine, combined with other therapies such as methotrexate and/or sulfasalazine, still had significantly lower anti-SARS-CoV spike IgG antibody titers than those who did not receive such a combination [96].

In terms of the safety of vaccination, the overall estimate from the meta-analysis showed a significantly higher risk for patients with autoimmune disease experiencing local and systemic adverse events after a first dose of COVID-19 inactivated vaccine in comparison with healthy controls; however, no statistically significant difference after a second dose of vaccine was observed. Higher frequencies of adverse events were reported among seropositive patients than in seronegative patients and healthy controls [72]. No moderate or severe adverse events related to the vaccine were reported [72,75,76,77]. Vaccine-related adverse events after the inactivated COVID-19 vaccine, especially systemic symptoms, were fewer than those reported with the mRNA vaccine [75]. 

In our systematic review, flare (worsening of autoimmune disease activity) was observed in more than 10% of patients with SLE after primary mRNA vaccination [70], and in patients with hematologic autoimmune diseases including immune-mediated thrombotic thrombocytopenic purpura and immune thrombocytopenia after a booster mRNA vaccination [32,42]. Meanwhile, this occurred in less than 5% of patients with multiple sclerosis [29] and with systemic autoimmune diseases including cryoglobulinemic vasculitis, rheumatoid arthritis, systemic lupus erythematosus, and systemic sclerosis after primary mRNA vaccination [8]. For the other vaccine types, flare was observed in 7% of autoimmune skin disease patients after primary inactivated virus vaccine and in less than 5% of SLE and autoimmune rheumatic disease patients [77,80]. Other adverse events, such as face tingling, herpes reactivation, bleeding, and urinary tract infection, also occurred in a small number of patients, together with severe adverse events such as high blood pressure, immune thrombocytopenic purpura, myocarditis, and death [29,80,101]. However, causal and temporal relationships between vaccine administration and adverse events or worsening disease activity following vaccination were difficult to determine due to limited data and the lack of a specific analysis of the causal relationship.

Based on our qualitative findings, breakthrough infections occurred less frequently in autoimmune patients after a booster dose. Autoimmune patients still had lower humoral and cellular responses even after having a third vaccine dose. Most of the patients were on immunosuppressant therapy, while untreated patients had better humoral and cellular responses. These findings support some previous evidence regarding the effects of booster vaccination. Regardless of the lower antibody titers in autoimmune patients, a potential increase in titer could be achieved after administering a third dose of vaccine, though the titer was still lower compared with a healthy control group. Evidence from a study by Joudeh et al. indicates that a booster vaccine dose is associated with a higher seroconversion rate, particularly in patients with a history of COVID-19 infection [108]. Further evidence comes from Cardelli, et al., who showed that a time-dependent decrease in protective antibody titer was restored after receipt of a booster dose. After a booster dose, five of nine non-responders developed adequate anti-RBD and neutralizing antibody titers. Three of them reduced their dose of or discontinued mycophenolate mofetil or azathioprine therapy before booster administration [109]. In addition, in terms of efficacy and safety, Dreyer at al. found no relapse activity or breakthrough infections after the third dose of vaccine [34].

This study has several limitations. First, the number of studies used to combine the efficacy, immunogenicity, and safety findings was relatively small. Second, considering that only one RCT was available comparing patients with autoimmune diseases and healthy controls after a primary dose of COVID-19 vaccine, we only included non-randomized studies. Third, since we only included a small number of studies in our meta-analysis, we might have significant publication bias. However, we also included pre-printed studies in our systematic review to reduce the possibility of this bias. Fourth, the variety of autoimmune diagnoses and immunosuppressive treatments could have an impact on the outcome of COVID-19 vaccination. This could affect our meta-analysis, and we could not address this by subgroup analysis due to the limited studies available. 

## 5. Conclusions

In conclusion, from this meta-analysis, we found that patients with autoimmune diseases showed significantly more breakthrough COVID-19 infections and lower total antibody (TAb) titers, IgG seroconversion, and neutralizing antibodies after inactivated COVID-19 vaccination compared with healthy controls. They also had more local and systemic adverse events after a first dose of inactivated vaccination compared with healthy controls, but this result was not seen after a second dose. Patients with autoimmune diseases also showed significantly lower TAb titers and IgG seroconversion after COVID-19 mRNA vaccination compared with healthy controls.

A second dose of vaccine was, however, found to be important, since it is associated with improved antibody titers and seroconversion. It is important to consult a healthcare provider before taking a vaccine, since immunosuppressants might affect the immunogenicity of vaccines. Additionally, the administration of third doses of COVID-19 vaccines should be considered due to improved seroprotection in these patients.

## Data Availability

The datasets generated and analyzed during the current study are available from the corresponding author upon reasonable request.

## Supplementary Materials

The following supporting information can be downloaded at: https://www.mdpi.com/article/10.3390/vaccines11091456/s1, Table S1: Search strategy; Table S2: Summarize of research articles; Table S3: GRADE asessement; Figure S1: (A): Quality assessment of RCT studies, (B): Quality assessment of non-RCT studies, (C): Quality assessment of case-series studies, (D): Quality assessment of cross-sectional studies; Figure S2: Funnel plot of IgG seroconversion after mRNA vaccination.
