# Supplementary material for: Efficacy, Immunogenicity, and Safety of COVID-19 Vaccines in Patients with Autoimmune Diseases: A Systematic Review and Meta-Analysis
Source: Vaccines (Basel). 2023 Sep 4;11(9):1456. doi: 10.3390/vaccines11091456 (PMC10535431; doi:10.3390/vaccines11091456)
Supplement: Supplementary file 1 [file vaccines-11-01456-s001.zip › vaccines-2533498-supplementary.pdf]

Supplementary Materials

Table S1. Search strategy

No	E-Lib	Re-Search strategies
1	Embase<br>(197 results)	autoimmune AND ('vaccine'/exp OR vaccine OR 'vaccination'/exp OR vaccination OR 'immunization'/exp OR immunization) AND ('covid 19'/exp OR 'covid 19') AND ('randomized controlled trial' OR 'non randomized controlled trial' OR cohort OR retrospective OR observational) AND [article]/lim
2	Pubmed<br>(100 results)	autoimmune[Title/Abstract] AND (vaccine[Title/Abstract] OR vaccination[Title/Abstract] OR immunization[Title/Abstract] OR vaccine[MeSH Terms] OR vaccination[MeSH Terms] OR immunization[MeSH Terms]) AND ("covid 19"[Title/Abstract] OR "covid 19"[MeSH Terms]) AND ("randomized controlled trial"[Title/Abstract] OR "non randomized controlled trial"[Title/Abstract] OR cohort[Title/Abstract] OR retrospective[Title/Abstract] OR observational[Title/Abstract])
3	Ebsco<br>(66 results)	AB autoimmune AND AB (vaccine or vaccines or vaccination or immunization or immunizations) AND AB covid-19 AND AB (randomized controlled trial OR non randomized controlled trial OR cohort OR retrospective OR observational) NOT AB review
4	MedRxiv<br>(282 results)	PI + randomized controlled trial **(26 results)**<br>"autoimmune AND (vaccine OR vaccination OR immunization) AND "covid-19" AND "randomized controlled trial" NOT review"<br><br>PI + non randomized controlled trial **(66 results)**<br>"autoimmune AND (vaccine OR vaccination OR immunization) AND "covid-19" AND "non randomized controlled trial" NOT review"<br><br>PI + cohort **(52 results)**<br>"autoimmune AND (vaccine OR vaccination OR immunization) AND "covid-19" AND "cohort" NOT review"

		PI + retrospective **(72 results)**<br>"autoimmune AND (vaccine OR vaccination OR immunization) AND "covid-19" AND "retrospective" NOT review"<br><br>PI + observational **(66 results)**<br>"autoimmune AND (vaccine OR vaccination OR immunization) AND "covid-19" AND "observational" NOT review"
5	BioRxiv<br>(286 results)	PI + randomized controlled trial **(36 results)**<br>"autoimmune AND (vaccine OR vaccination OR immunization) AND "covid-19" AND "randomized controlled trial" NOT review"<br><br>PI + non randomized controlled trial **(101 results)**<br>"autoimmune AND (vaccine OR vaccination OR immunization) AND "covid-19" AND "non randomized controlled trial" NOT review"<br><br>PI + cohort **(44 results)**<br>"autoimmune AND (vaccine OR vaccination OR immunization) AND "covid-19" AND "cohort" NOT review"<br><br>PI + retrospective **(9 results)**<br>"autoimmune AND (vaccine OR vaccination OR immunization) AND "covid-19" AND "retrospective" NOT review"<br><br>PI + observational **(96 results)**<br>"autoimmune AND (vaccine OR vaccination OR immunization) AND "covid-19" AND "observational" NOT review"
6	Proquest<br>(23 results)	ab(autoimmune) AND ab(vaccin[*5] OR immunization) AND ab("covid 19") AND ab("randomized controlled trial" OR "non randomized controlled trial" OR cohort OR retrospective OR observational)

7	SSRN<br>(1 result)	autoimmune covid 19 vaccination efficacy immunogenicity safety
8	EuroPMC<br>(29 results)	(autoimmune AND "covid 19" AND (vaccination OR vaccination OR immunization) AND efficacy AND immunogenicity AND (safety OR "adverse event" OR "adverse effect")) AND ((SRC:PPR AND HAS_PUBLISHED_VERSION:Y))
9	Cochrane<br>(70 results)	Search Name:<br>Date Run:<br>19/09/2022 21:48:17<br>Comment:<br>IDSearchHits<br>#1 (autoimmune):ti,ab,kw (Word variations have been searched)<br>5850<br>#2 MeSH descriptor: [Autoimmune Diseases] explode all trees<br>20627<br>#3 #1 OR #2<br>25138<br>#4 (COVID-19):ti,ab,kw (Word variations have been searched)<br>11869<br>#5 MeSH descriptor: [COVID-19] explode all trees<br>2207<br>#6 #4 OR #5<br>11869

		#7 (vaccine):ti,ab,kw (Word variations have been searched)<br><br>28836<br>#8 (vaccination):ti,ab,kw (Word variations have been searched)<br><br>28836<br>#9 (immunization):ti,ab,kw (Word variations have been searched)<br><br>47856<br>#10 #7 OR #8 OR #9<br><br>62267<br>#11 MeSH descriptor: [Vaccines] explode all trees<br><br>14120<br>#12 #10 OR #11<br><br>62278<br>#13 #3 AND #6 AND #12<br><br>70

Table S2. Summarize of research articles

No	Authors	Study Characteristics				Participant Characteristics			Vaccine Information	Outcome			
		Country	Research Population *(Autoimmune)*	Study Design; Sample Size (Total Number of Randomized or Total Population at Start of Study)	Inclusion	Immunosuppressant or Immunodulatory Therapy	Age; sex	Comorbidities	COVID-19 Vaccine Platform; administration	Efficacy	Immunogenicity		Safety
											Parameter	Outcome	
mRNA vaccine (n=37)													
1	Achiron, et al (2021)	Israel, single-center	MS (555)	Observational study; 555 patients	MS patients treated in Sheba MS Center who agreed to receive COVID-19 vaccine (30 µg of BNT162b2 vaccine)	β-interferons, GA, teriflunomide, DMF, natalizumab, fingolimod, OCR, alemtuzumab, cladribine, RTX, IVIG, no immunosuppressants	≥18 years; male and female	No data	Pfizer/BioNTech (BNT162b2); primary	Breakthrough COVID-19 infections occurred in 3 of 555 patients after the first dose of vaccine administration and no infections were reported after the second vaccine dose.	N/A	N/A	There were 8 of 555 and 5 of 435 patients who developed an autoimmune relapse after the first and second vaccination, respectively. Local symptoms occurred in 89 of 555 and 62 of 435 patients after the first and second vaccination, respectively. Systemic symptoms occurred in 100 of 555 and 193 of 435 patients after the first and second vaccination, respectively. Face tingling also occured in 3 of 555 and 5 of 435 patients after the first and second dose, respectively. Otherwise, one of 435 patients who had a second dose of vaccine died but considered by the authors not related to vaccination.
2	Achiron, et al (2022)	Israel, single center	MS (172)	Observational cohort study; 172 patients	MS patients that performed pre-vaccination lymphocyte count and received the full COVID-19 vaccination.	Cladribine, OCR, fingolimod	Median age in the treatment groups, respectively: 43.1, 44.9, 53.2, 50.5 years. Median age in the health subjects: 54.3 years; male and female	No data	Pfizer/BioNTech (BNT162b2); primary	N/A	IgG titer, seroconversion, lymphocyte count	1) All MS patients under cladribine treatment developed a high level of antibodies post-COVID-19 vaccination (p=0.99). Meanwhile, MS patients under fingolimod and OCR treatment failed to develop a post-vaccination humoral response (p<0.0001).<br><br>2) Cladribine have 900 (630-1305) lymphocyte, Fingolimod 555 (402-722), OCR 1940 (1395-2370), Fingolimoid treated MS: 10/26 (38.5%) had a lymphocyte count below 500 cells/mm3, and an additional 13/26 (50%) had lymphocyte count between 500 and 1000 cells/mm3. Only one patient treated with Fingolimod (3.8%) showed a humoral response, OCR-treated MS: 42/44 (95.5%) patients had lymphocyte coutns above 1000 cells/mm3	N/A
3	Achiron, et al (2021)	Israel, single-center	MS (20)	RCT; 20 patients	Relapsing–remitting MS according to McDonald criteria, age ≥18 years, under treatment with fingolimod for at least 12 months, received full 2- dose Pfizer BNT162b2 COVID-19 vaccination, failed to develop COVID-19 IgG antibody response, signed written informed consent.	Fingolimod	≥18 years; male and female	No data	Pfizer/BioNTech (BNT162b2); booster	N/A	IgG titer, seroconversion, lymphocyte count	1) A higher rate of patients in the fingolimod-discontinuation group [n=8/10] compared to fngolimod-continuation group [n=2/10] developed positive SARS-COV-2 IgG. Median IgG titer 1 month following the third dose was 202.3 BAU/ml vs. 26.4 BAU/ml, respectively, p=0.022.<br><br>2) CD19 B cells in fingolimod-discontinuation was 202 cells/mm3 (normal range 50–300 cells/mm3) and finglimod-continuation were much lower, 4 and 10 cells/mm3.	N/A

												3) CD4 T cells were 155 cells/mm3 (normal range 436–1394 cells/mm3) and CD8 T cells were 715 cells/mm3 (normal range 166–883 cells/mm3) in the patient who stopped fngolimod treatment following the third vaccine dose, while both CD4 and CD8 were decreased in the two patients who continued fngolimod treatment (CD4 T cells were 4 and 19 cells/mm3 and CD8 were 35 and 104 cells/mm3.	
4	Aharoni, et al (2022)	Israel, single-center	iTTP (93)	Retrospective cohort; 93 patients	ITP patients above the age of 18 years who received first, second or third (booster) doses of Pfizer-BioNTech vaccine after a prior diagnosis of ITP.	Corticosteroids (prednisone, DEX), IVIG, RTX	≥18 years; male and female	Ischemic heart disease, COPD, asthma, malignancy, SLE	Pfizer/BioNTech (BNT162b2); primary and booster	No primarily vaccinated patient was hospitalized.	N/A	N/A	There were 10 of 93 and 8 of 69 patients who had an autoimmune relapse after primary and booster vaccination, respectively.
5	Ammitzbøll, et al (2021)	Denmark, single center	SLE (61), RA (73)	Prospective cohort; 134 patients	Patient with SLE: fulfillment of the American College of Rheumatology (ACR) 1982 revised classification criteria for SLE; patient with RA were fulfillment of either the 1987 ACR or the 2010 ACR/EULAR Classification Criteria and treatment with either a biological or small molecule DMARD. All patients resided in the same geographical region of Denmark (Central and North Region).	MTX, salazopyrin, HCQ, prednisone, leflunomide, AZA, MMF, TNFi, RTX, IL-6 inhibitor, abatacept, BEL, JAKi	Median age --> SLE: 60.2 years, RA: 70.3 years; male and female	Hypertension	Pfizer/BioNTech (BNT162b2); primary	N/A	IgG titer, seroconversion	Fewer patients with RA (67%) than those with SLE (89%) had measurable antibodies against SARS-CoV-2 after the second dose of vaccine. However, the difference observed with diagnosis was nonsignificant when adjusting for treatment with RTX (p=0.28). RTX were significantly associated with no antibody vaccine response after adjusting for diagnosis and HCQ	N/A
6	Bieber, et al (2022)	Israel, multi-center	Total (127928); RA (31573), SLE (2808), SpA (31262), scleroderma (4143), myositis (1880), systemic vasculitis (AAV, Polyarteritis Nodosa, Giant cell arteritis, Behcet's syndrome) (29267), other (SJ, ankylosing spondilitis, PsA, familial mediteranian fever, APS) (26985)	Retrospective cohort; 127,928 patients	CHS (Clalit Health Services) members diagnosed with ARD older than 18 years	prednisone, MTX, csDMARDs, colchicine, AZA, MMF, TNFi, IL-6 inhibitor, IL-17 inhibitor, IL-12/23 inhibitor, abatacept, RTX, JAKi, calcineurin inhibitor	>18 years; male and female	Heart failure, myocardial infarction, hypertension, diabetes, obesity, COPD, asthma, other lung disease, CVA/TIA, malignancy, chronic kidney disease,	Pfizer/BioNTech (BNT162b2); primary and booster	There were 3,498 and 770 of 31,407 and 6,971 patients who had breakthrough COVID-19 infections after the primary and booster dose of the vaccine, respectively. Of those who had been infected, 138 and 45 patients who had primary and booster vaccination were hospitalized, respectively. Thirty-two patients who had primary dose died.	N/A	N/A	N/A
7	Costa, et al (2022)	Italy, single-center	Autoimmune (60)	Cohort; 60 patients	All University Hospital Città della Salute e della Scienza di Torino (CSS) workers who were vaccinated in January-February 2021.	N/A	No data; male and female	No data	Pfizer/BioNTech (BNT162b2); primary	N/A	T-cell response	1) The self-reporting of an auto-immune disease was consistently associated with a lower T-cell response for all the Ag, with significant results for Ag1 and Ag3 positivity.	N/A
8	De Santis, et al (2022)	Italy, single-center	Total (287), RA (72), SpA (125), SS (13), SLE (12), dermato/polimiositosis (3), AIH (31), PBC (13), other rheumatic disease (22)	Observational prospective cohort; 287 patients and 67 controls	Patients with rheumatic and chronic inflammatory diseases who agreed to participate and provided written consent and received two doses of vaccine 4 weeks apart (17-19 April and 19-22 May 2021)	MMF, glucocorticoids, MTX, AZA, TNFi (adalimumab, etanercept, golimumab, certolizumab, infliximab), IL-17 inhibitor, IL-6 inhibitor, JAKi, other therapies, no immunosuppressants	Median age --> patients: 55 years; controls: 48 years; male and female	Hypertension, diabetes, cardiovascular disease, ex- COVID (history of COVID- 19 and/or positive serology at baseline)	Moderna (mRNA- 1273); primary	There were 2 of 287 patients and 1 of 67 controls who had breakthrough COVID-19 infections after the first of primary vaccine dose. There were no severe cases reported.	IgG titer, seroconversion, neutralization antibodies, T-cell response	1) At T2, anti-SARS-CoV-2 titers were significantly lower compared to the controls only in the patients taking mycophenolate ($p < 0.0001$); moreover, the patients on mycophenolate had significantly lower anti-SARS-CoV-2 titers than the patients treated with any other immunosuppressants<br><br>2) Anti-SARS-CoV-2 antibodies were positive in 96% of the patients and 100% of the controls after receiving the second vaccine dose. Eight	There were 152 of 219 patients and 35 of 45 controls who had systemic symptoms after primary vaccination. Mild and moderate adverse events occurred in 105 and 46 of 219 patients, respectively. Mild and severe adverse events also occurred in 26 and 9 of 45 controls, respectively. Severe adverse events occurred in 1 patient with high blood pressure and metrorrhagia after the second dose of vaccine.

												patients did not achieve seroconversion even after receiving the second dose and were all on mycophenolate (p<0.0001). Moreover, they were all COVID-naïve patients affected by 68% of systemic sclerosis patients (p<0.0001).<br><br>3) Statistical analysis was not performed considering the small sample size; nonetheless, the neutralizing activity seemed similar in the ex-COVID patients compared to controls, while it was reduced in the COVID-naïve patients<br><br>4) Cytotoxic T cell response did not seem different in the eight COVID-naïve patients treated with different immunosuppressants (ongoing at time T2 of blood sample collection: two treated with MMF at 3 g/day, three with baricitinib at 4 mg/day, and three with adalimumab at 40 mg/2 weeks) compared to the four COVID-naïve controls	
9	Deepak, et al (2021)	USA, multi-center	IBD (42), RA (38), SpA (20), SLE (15), CTD (4), SJ (8), vasculitis (5), autoinflammatory syndrome (2), MS (9) NMOD (1), IgG4-Related Disease (2), APS (1)	Longitudinal observational cohort study; 133 patients and 53 immunocompetent controls	CID patients: Able to understand and give informed consent, capable of attending all mandatory study visits according to the study schedule, males or females between over age 18, or the chronic inflammatory disease (CID) cohort, have health care provider-documented CID. Immunocompetent controls: must in good health as determined by medical history and physical exam and not be on any immunomodulatory nor immunosuppressive medications.	Prednisone, MTX, HCQ, MMF, AZA, leflunomide, sulfasalazine, tofacitinib, upadacitinib, TNFi, aCD20-BCD, BEL, vedolizumab, IL-12/23 inhibitor, IL-23 inhibitor, abatacept, tocilizumab, canakinumab, fingolimod, ibrutinib, NSAIDs, no immunosuppresant	≥18 years; male and female	Excluded	Pfizer/BioNTech (BNT162b2) or moderna (mRNA-1273); primary	N/A	IgG titer, seroconversion, neutralization antibodies	1) CID participants on B cell depleting therapies, prednisone, JAK inhibitor, and antimetabolites all had statistically significant reductions in antibody titers when compared to immunocompetent participants.<br><br>2) although CID participants averaged a 3-fold reduction in antibody titers compared to immunocompetent controls. This diminished antibody response was associated with a similar 2.7-fold decrease in neutralization of viral infection.	N/A
10	Dinoto, et al (2022)	Italy, multi-center	Inflammatory and autoimmune CNS patients (61); limbic encephalitis (31), autoimmune encephalitis (15), cerebellar ataxia (5), SPS (4), morvan/isaacs syndrome (3), autoimmune epilepsy (3)	Retrospective cohort; 66 patients	Patients with serum and/or CSF positivity for specific neuronal autoantibodies, a compatible neurological syndrome, available follow-up ≥6 weeks after vaccination with any of the approved SARS-CoV-2 vaccines	Oral steroids, intravenous immunoglobulins, AZA, MMF, RTX, tocilizumab, RTX+oral steroids, AZA+oral steroids	17-85 years; male and female	No data	Pfizer/BioNTech (BNT162b2) or moderna (mRNA- 1273); primary and booster	N/A	N/A	N/A	There were 5 of 66 patients who had an autoimmune relapse after vaccination. Local symptoms occurred in 16 of 66 and 9 of 58 patients after the first and second dose of vaccine, respectively. Moreover, systemic symptoms occurred in 8 of 66 and 17 of 58 patients after the first and second dose, respectively. Herpes reactivation occured in 1 patient after the first vaccine dose.
11	Dreyer-Alster, et al (2022)	Israel, single-center	MS (211)	Observational prospective study; 211 patients	MS patients who previously received two intramuscular injections.	no immunosuppressants, β-interferons, GA, teriflunomide, DMF, natalizumab, fingolimod, OCR, alemtuzumab, cladribine, RTX, IVIG	≥18 years; male and female	No data	Pfizer/BioNTech (BNT162b2); booster	There was 1 patient who had been infected by COVID-19 after the third dose or booster vaccine.	IgG titer	There was no difference in IgG levels between untreated patients and patients treated with vaccination-safe DMTs (p = 0.56).	There were 7 of 211 patients who had an autoimmune relapse after the booster vaccination. Forty-six of 211 patients had local symptoms and 69 of 211 patients had systemic symptoms.
12	Fabris, et al (2022)	Italy, single-center	AID (28)	Cohort; 28 patients	Those who had received the first dose of vaccine within 5 months from the last RTX infusion, only SLE patients in remission or with low disease activity according to the Lupus Low Disease Activity State (LLDAS), without the	BEL, RTX, prednisone, HCQ, MTX, MMF, AZA, calcineurin inhibitors	Mean age --> patients: 61 years (rituximab), 47 years (belimumab), control: 46 years; male and female	No data	Pfizer/BioNTech (BNT162b2) or moderna (mRNA-1273); primary	Symptomatic COVID-19 infection in 2 patients (mean follow-up period: 8.8 ± 0.7 months from the second dose vaccine).	IgG titer, neutralization antibodies, T-cell response	1) The median antibody titer in the RTX or BEL patients was significantly lower than that in the controls (39.6 AU/ml vs. 1133 AU/ml, p = 0.002).	N/A

					need for any changes in the drug schedule due to the vaccination program. The mean follow-up period was 8.8 ± 0.7 months from the second dose of the vaccine. During the follow-up period, two patients enrolled in this study developed symptomatic COVID-19 infection.							2) no significant difference was observed in the ability of sera samples from both groups to neutralize either the WT pseudovirus (IC50 = 862.4 [272.5–4872] for controls and 1771 [136.0–4039] for patients) or the UK pseudovirus [IC50 347 [278–22,905] for controls and 395.7 [13.11–1563] for patients]<br><br>3) Only one (9%) of RTX patients developed anti-RBD antibodies, with significantly lower titer (p<0.0001). The majority of BEL patients (94.1%) developed anti-RBD antibodies even though their average titer was significantly lower than that of the controls (p=0.002).<br><br>4) The result of IGRA was positive in 8 of the 11 (72.7%) RTX patients and 16 of the 17 (94.1%) BEL patients<br><br>5) IFN release in both the RTX and BEL patients was comparable to that in the control participants.<br><br>6) median IL-2 level (T-cell response) of the patients was similar to that of the controls (230.28 [52.7–459.0] pg/ml vs. 245.2 [94.9–538.0] pg/ml]	
13	Ferri, et al (2021)	Italy, multi-center	ASD (478)	Prospective cohort; 478 patients and 502 controls	Patients with rheumatoid factor (RF) and/or anti- citrullinated protein antibodies (ACPA) positive rheumatoid arthritis (RA), systemic lupus erythematosus (SLE), systemic sclerosis (SSc), cryoglobulinemic vasculitis (CV), and a miscellanea of other systemic vasculitis	MTX, sulfasalazine, HCQ, leflunomide, MMF, AZA, TNFi, abatacept, RTX, IL-6 inhibitor, JAKi, BEL	Mean age: 59 years; male and female	Skin ulcer, renal involvement, pulmonary arterial hypertension, gastrointestinal involvement, interstitial lung disease	Pfizer/BioNTech (BNT162b2) or moderna (mRNA-1273); primary	N/A	IgG titer, seroconversion	1) Significantly lower IgG levels in ASD series compared to controls [286 (53–1203) vs 825 (451–1542) BAU/mL, p < 0.0001], as well as between single ASD subgroups and controls.<br><br>2) Higher percentage of non-responders to vaccine was recorded in ASD patients compared to controls [13.2% (63/478), vs 2.8% (14/502); p < 0.0001].<br><br>3) Increased prevalence of non-response to vaccine was also observed in different ASD subgroups, in patients with ASD-related ILD (p = 0.009), and in those treated with glucocorticoids (p = 0.002), MMF (p < 0.0001), or RTX (p < 0.0001).	Ten of 478 patients developed an autoimmune relapse after primary vaccination. Local symptoms occurred in 182 of 478 patients, meanwhile 108 systemic symptoms cases were reported among 478 patients.
14	Furer, et al (2021)	Israel, multi-center	AIIRD (686); RA (236), PsA (165), AS (68), SLE (101), IIM (19), LVV (21), AAV (26), other vasculitis (23)	Prospective cohort; 686 patients and 121 controls	Adult patients (aged ≥18 years), rheumatoid arthritis (RA)/ACR/European League Against Rheumatism (EULAR) 2010 classification criteria; psoriatic arthritis (PsA)/Classification Criteria for PsA; axial spondyloarthritis (axSpA)/Assessment of SpondyloArthritis International Society classification criteria; systemic lupus	Glucocorticoid, MTX, TNFi, IL-6 inhibitor, aCD20-BCD, abatacept, JAKi, IL-17 inhibitor, MMF	≥18 years; male and female	Diabetes mellitus and ischaemic heart disease	Pfizer/BioNTech (BNT162b2) or moderna (mRNA-1273); primary	No COVID-19 symptomatic disease after the first vaccine dose, whereas one subject in the control group was diagnosed with mild COVID-19 after the second vaccine dose.	IgG titer, seroconversion	1) Following vaccination, the seropositivity rate and S1/S2 IgG levels were significantly lower among patients with AIRD versus controls (86% (n=590) vs 100%, p<0.0001 and 132.9±91.7 vs 218.6±82.06 BAU/mL, p<0.0001, respectively).	No significant difference in mild adverse events between patients with AIIRD and controls. No serious or major adverse events in the control group. After second vaccine dose, two patients with AIIRD died. In patients with RA, PsA, axSpA and SLE, the postvaccination

					erythematosus (SLE)/1997 ACR10 or 2012 Systemic Lupus Erythematosus International Collaborating Clinics criteria; systemic vasculitis: large vessel vasculitis (LVV), antineutrophil cytoplasmic antibody-associated vasculitis (AAV), including granulomatosis with polyangiitis (GPA), microscopic polyangiitis and eosinophilic GPA/Chapel Hill Consensus Conference definitions; central nervous system (CNS) vasculitis, including primary CNS vasculitis, neuro-Behcet and Susac syndrome; and idiopathic inflammatory myositis (IIM) /EULAR/ACR classification criteria.							2) RTX was the main cause of a seronegative response (39% seropositivity)<br><br>3) aCD20-BCD significantly impaired vaccine's immunogenicity, with the lowest seropositivity rate of 39%.	indices of disease activity remained stable.
15	Furer, et al (2022)	Israel, multi-center	AIIRD (86); RA (49), AAV (23), IIM 18, SLE (11), vasculitides (6)	Prospective cohort; 108 patients and 122 immunocompetent controls	Patients: Adult (≥18 years of age) AIIRD patients who fulfilled the ACR/EULAR criteria for RA, Systemic Lupus International Collaborating Clinics classification criteria for systemic lupus erythematosus (SLE), Chapel Hill classification criteria for systemic vasculitis (AAV, giant cell arteritis, other systemic vasculitides), and the EULAR/ACR classification criteria for idiopathic inflammatory myopathy (IIM). Control: immunocompetent individuals	csDMARDs, MTX, prednisone, leflunomide, MMF, IVIG	≥18 years; male and female	History of lymphoma	BNT162b2 (Pfizer- BioNTech); primary	N/A	IgG titer	1) The BNT162b2 vaccine-induced positive immunogenic response rate and serum S1/S2 IgG titers were significantly lower in the AIRD-RTX group compared to controls ($p < 0.0001$).<br><br>2) The lowest S1/S2 IgG titer (BAU, mean ± SD) was detected in the AAV and IIM patients (36.25 ± 73 and 25.19 ± 45.07, respectively), followed by patients with other non-AAV systemic vasculitides (48.8 ± 74.29), whereas the highest titers were detected in patients with SLE and RA (99.84 ± 110.55 and 55.19 ± 81.55, respectively)<br><br>3) The rate of seropositive and seronegative vaccine response was similarly distributed across all rheumatic diseases, except for the SLE patients who had a high prevalence of a seropositive immunogenic response (81.82% [n = 9] vs. 18.18% [n = 2], p = 0.007, respectively)	Local symptoms (64 and 59 cases of 106 patients) after the first and second dose of vaccine; systemic symptoms (23 and 73 cases of 106 patients) after the first and second dose of vaccine, respectively. Other adverse events such as palpitations, pruritus, weakness, throat pain, night sweats, feeling heavy, vaginal pain, pericarditis, ear pain, sweating, dizziness, and nausea (10 and 12 cases of 106 patients each after first and second dose of vaccine). RA disease activity after vaccination: worsened (26.5–32.6%), stable (52.9–60%), or improved (12.5–20.6%). SLE disease activity remained stable.
16	Furer, et al (2021)	Israel, multi-center	AIIRD (419)	Observational - case series; 6	Patients with AIIRD, including rheumatoid arthritis (RA), spondyloarthropathies, connective tissue diseases (CTD), vasculitis and myositis)	HCQ, tofacitinib, upadacitinib, prednisone, MMF, RTX, prednisone, tocilizumab	36-61 years; female	No data	BNT162b2 (Pfizer- BioNTech); primary	N/A	N/A	N/A	Local and systemic symptoms occurred in 5 and 3 of 6 patients after vaccination, respectively. No flare of rheumatic disease was reported during that period.
17	Geisen, et al (2021)	Germany, single-center	CID (26); PsA (2), RA (6), MCTD (1), SpA (3), sarcoidosis (1), Giant cell vasculitis (1), Psoriasis (4), Chrons disease (2), SLE (2), Myositis (1), MS/Chrons disease (1)	Cohort; 26 patients and 42 healthy controls	No data	Golimumab, certolizumab, etanercept, infliximab, tocilizumab, ixekizumab, vedolizumab, adalimumab, secukinumab, ustekinumab, BEL, leflunomide, HCQ, sulfasalazine, AZA, prednisolone	24-89 years; male and female	No data	Pfizer/BioNTech (BNT162b2) or moderna (mRNA-1273); primary	N/A	IgG titer, IgA titer, neutralising antibody	1) Healthy control group showed a mean anti-SARSCoV-2-IgG titre of 2685 BAU/mL (±1102, 793–3840), patients with CID exhibited significantly lower levels of specific immunoglobulins against the SARS-CoV-2 spike protein (mean 2053 BAU/mL±1218, 98.2–3840) 7days after the secondary immunisation (p=0.037)<br><br>2) Patients with CID had lower specific IgA levels compared with healthy controls (mean	Local, systemic, and other adverse events (23, 43, and 9 cases in 26 patients) after the second dose of vaccination. Mild systemic side effects were more common in CID patients to healthy controls (fatigue 53.8% vs 43.2%, myalgia 42.3% vs 31.6%, respectively). No inflammatory arthritis flares (delta DAS28 >0.6).

												24.52±30.48U/mL vs 47.65±45.12U/mL; p=0.0035).<br><br>3) Patients with CID also had lower levels of NAb, with a mean inhibitory activity level of 96.04% detected in healthy controls (±1.551, 91–97), whereas patients presented with a mean inhibitory level of 87.42% (±17.94, 37–97; p=0.0442)	
18	Geisen, et al (2022)	Germany, single-center	CID (10)	Cohort; 10 patients and 24 healthy individuals	Healthcare workers and other risk groups who received their first SARS-CoV-2 vaccination in January 2021 followed by a second vaccination 5 or 3 weeks later.	TNFi, oDMRADs	Mean age: 43 years; male and female	No data	Pfizer/BioNTech (BNT162b2) or moderna (mRNA- 1273); primary and booster	N/A	IgG titer, IgG avidity, neutralizing antibodies, plasma cells count	1) Six months after the second vaccination, anti-SARS-CoV-2 IgG levels, IgG avidity and anti-pre-VOC NAb titres were significantly reduced in anti-TNF-α recipients compared to controls (healthy individuals: avidity: p ≤ 0.0001; NAb: p = 0.0347; oDMARDs: avidity: p = 0.0012; NAb: p = 0.0293).<br><br>2) Under TNFi therapy, anti-S-IgG levels after the second immunization were significantly lower than in patients receiving other DMARDs or in healthy controls. Anti-BA NAb were detectable in all subjects except patients taking a TNFi after the third vaccination.<br><br>3) The number of plasma cells was increased in TNFi patients (Healthy individuals: p = 0.0344; oDMARDs: p = 0.0254), while the absolute number of SARS-CoV-2-specific plasma cells 7 days after 2nd vaccination were comparable	N/A
19	Giannoccaro, et al (2022)	Germany, single-center	Autoimmune neurological conditions (300) MS, MG, chronic inflammatory neuropathy, autoimmune encephalitis and other antibody-mediated CNS disorders, i.e.NMOSD, SPS	Longitudinal observational study; 300 patients and 347 healthcare-workers	Age ≥18 years, ascertained ANC, ability to sign the consent form	no immunosuppressants, corticosteroids, IVIG, PLEX, AZA, aCD20-BCD, OCR, RTX, DMF, cladribine, natalizumab, fingolimod, GA, β-interferons, teriflunomide	≥18 years; male and female	Diabetes type 2, hypertension, dislipidemia, other autoimmune disorders	Pfizer/BioNTech (BNT162b2) or moderna (mRNA-1273); primary	No severe case was reported.	IgG titer, seroconversion	1) Anti-S(RBD) specific IgG were detected in 89.9% patients and in all controls (p < 0.0001), with no difference in the antibody median levels between the two groups.<br><br>2) Significantly lower antibody levels were found in patients undergoing aCD20-BCD mAb (p<0.0001), fingolimod (p < 0.0001), AZA (p = 0.011) and steroids (p=0.035) compared to patients without immunotherapy.	Local symptoms after first and second dose (212 cases each), systemic symptoms after first and second dose (125 and 197 cases, respectively). Other adverse events such as nausea, dysgeusia, paresthesia, sleepiness after first and second dose (19 cases each, respectively). Predicting factors of moderate-severe side effects: male sex and older age (lower risk of local and systemic reactions); mRNA vaccines (increase risk local and systemic reactions). There was no difference in the incidence of relapse before and after vaccinations.
20	Giuffrida, et al (2022)	Italy, single-center	iTTP (32)	Prospective cohort; 32 patient	Adult patients had a prior diagnosis of iTTP.	Corticosteroids, PLEX, caplacizumab	Median age: 47 years; male and female	No data	Pfizer/BioNTech (BNT162b2) or moderna (mRNA- 1273); primary and booster	N/A	N/A	N/A	Five of 32 patients developed an autoimmune relapse after receiving the third dose of vaccination. Characteristics found in relapse cases were: positive hemolysis markers, anemia, schistocytes, thrombocytopenia, unmeasurable ADAMTS13 activity (<10%), and increased anti-ADAMTS13 (>12-15 U/mL).

21	Hills, et al (2022)	New Zealand, single-center	Autoimmune (15)	Observational study; 15	Aged 18 years were eligible if they were presenting for their first COVID-19 vaccination, if they planned to attend the same vaccination centre for their second dose, and if they were able to provide written informed consent.	N/A	8-86 years; male and female	Hypertension	BNT162b2 (Pfizer-BioNTech); primary	N/A	IgG titer (titer >32.5 BAU/mL = positive), seroconversion	Six of eight autoimmune patients were seroconverted after receiving second dose of vaccine.	N/A
22	Kornek, et al (2022)	Austria, single center	Neuroimmunologic disorders (82); MS (64), NMOSD (7), myasthenic syndrome (7), autoimmune encephalitis (2), chronic inflammatory demyelinating polyneuropathy (2)	Prospective cohort; 82 patients and 82 age-and sex-matched healthy controls	Adult patients (≥18 years of age), with a confirmed diagnosis of an immune-mediated disease of the central nervous system, the peripheral nervous system, or the neuromuscular junction who were going to have a SARS-CoV-2 mRNA vaccination (Pfizer/BioNTech or Moderna)	RTX, OCR, AZA, tocilizumab, prednisone, IVIG, eculizumab, MMF, aCD20-BCD	≥18 years; male and female	No data	Pfizer/BioNTech (BNT162b2) or moderna (mRNA-1273); primary	N/A	Seroconversion, IgG titer, T cell response	1) SARS-CoV-2-specific antibodies were found less frequently in patients (70% [57/82]) compared with controls (82/82 [100%], p < 0.001).<br><br>2) Median antibody levels in patients (26.5 BAU/ml [IQR = 1,026]) were lower compared with healthy controls (1,711 BAU/ml [IQR = 1665], p < 0.001)<br><br>3) T cell responses were induced in 32 of 38 (84%) patients and 16 of 16 (100%) healthy controls with lower levels in patients (median SFCs/106 PBMC 183) compared with controls (median SFCs/106 PBMC 340)<br><br>4) T-cell response against the Wuhan strain and the Delta variant was more pronounced in frequency (p < 0.05) and magnitude (p < 0.01) in B-cell depleted compared to nondepleted patients	There were 1 of 79 and 2 of 78 patients who developed autoimmune relapse after the first and second dose of vaccine, respectively. There were 60 of 79 and 64 of 78 patients who had local symptoms after the first and second dose of vaccine, respectively. There were 73 cases in 79 patients and 136 cases in 78 patients who had systemic symptoms after the first and second dose of vaccine, respectively. Transient worsening of pre-existing neurologic symptoms occurred in 6 of 79 and 8 of 78 patients after the first and second dose of vaccine, respectively.
23	Lustig, et al (2021)	Israel, single center	Autoimmune (160)	Prospective cohort; 126 patients	Health-care workers at the centre who had a negative anti-SARS-CoV-2 IgG assay before receiving the first dose of the vaccine, and at least one serological test after the first dose of the vaccine.	N/A	≥18 years; male and female	No data	Pfizer/BioNTech (BNT162b2); primary	N/A	IgG titer, IgA titer, neutralising antibody	1) Total positive IgG were assessed in 7% autoimmune patients at week 3 and no difference was observed, as well as total neutralization and total IgA that had no difference compared to non-autoimmune participants.	N/A
24	Malipiero, et al (2021)	Italy, single-center	Autoimmune (5); RA (4), pemphigo (1)	Prospective cohort; 108 healthcare workers and 5 patients	Healthcare workers: n/a; Elderly patients: >75 years, not affected by immune-mediated systemic diseases and were not on long-term immunosuppressive drugs. ; Autoimmune patients: on disease-modifying antirheumatic drugs (methotrexate or mycophenolate) plus rituximab, 2 patients conducted SARS-CoV-2 serological assay, performed before enrollment, showed absent serological responses to the vaccine.	MTX, MMF, RTX	No data; male and female	No data	Pfizer/BioNTech (BNT162b2); primary	N/A	IgG titer, seroconversion, B cells count, T cell response	1) After the second dose of vaccine, the mean values of anti-RBD antibodies and INFγ were 123.33 U/mL (range 27.55–464) and 1513 mIU/mL (range 145–2500) in HCWs and 210.7 U/mL (range 3–500) and 1167 mIU/mL (range 83–2500) in elderly people<br>2) In autoimmune patients, IgG Anti-RBD levels and seroconversion was undetectable in 2 out of 5 patients.<br><br>3) CD19+B cell count was below the normal range but still detectable (<36 cells/uL in all patients, N:200-400)<br><br>4) In these two seronegative autoimmune patients, the peripheral CD19+B cells were virtually absent (normal range, 200–400 cell/μL), while their INFγ levels were>2500 mIU/mL	N/A

25	Mandl, et al (2022)	Austria, single center	SARD (80); SLE (33), SS (13), dermatomyositosis/polymyositis (4), MCTD (2), SJ (6), UCTD (3), AAV (3), Behcet's disease (1), LVV (3), polymyalgia rheumatica (10), Adult-onset Still's disease (1), sarcoidosis (1)	Prospective cohort; 82 patients and 82 age-matched and gender-matched healthy control	SARD patients: patients with SARD (antineutrophil cytoplasmic antibody-associated vasculitis, dermatomyositis/polymyositis, mixed connective tissue disease/undifferentiated connective tissue disease, polymyalgia rheumatica/large-vessel vasculitis, primary Sjögren's syndrome, systemic lupus erythematosus (SLE), systemic sclerosis); Healthy control: Individuals without known inflammatory rheumatic disease and no immunomodulatory therapy served as healthy controls (HC).	no immunosuppressants, MTX, MMF, HCQ, AZA, BEL, tocilizumab, tacrolimus, olumiant	Mean age: 52 years; male and female	hypertension, diabetes mellitus, hyperlipidemia, thyroid disorders and chronic obstructive pulmonary disease	Pfizer/BioNTech (BNT162b2); primary	N/A	Seroconversion, IgG titer	1) Patients with SARD, independent of treatment regimen, had significantly lower seroconversion (77% for no DMARD therapy, 56% for monotherapy and 57% for combination therapy, all p<0.0001) after the first dose as compared with HC<br><br>2) Patients with SARD on monotherapy or combination therapy also having a significantly lower seroconversion as compared with those receiving no DMARD therapy (56% vs 77%, p=0.01;57% vs 77%, p=0.01) as compared with those receiving no DMARD therapy (56% vs 77%, p=0.01;57% vs 77%, p=0.01)<br><br>3) After second dose, seroconversion was significantly lower for patients on combination DMARD therapy compared with all other groups (81% compared with 95% for monotherapy, p=0.01; 100% for both no DMARD therapy and HC, both p<0.0001).<br><br>4) IgG level of SARS-CoV-2 was significantly lower in the patient group compared with the HC group after both the first vaccination (median 5.40 (IQR 0–66.7) BAU/mL vs 33.5 (IQR 13.2–189) BAU/mL, p<0.0001) and the second vaccination (median 726.5 (IQR 211–2500) BAU/mL vs 1673 (IQR 915.5–2500) BAU/mL, p=0.003)	N/A
26	Mena-Vázquez, et al (2022)	Spain, multi-center	ILD-SAD (176); RA (105), SS (49), IIM (22)	A cross-sectional multicenter cohort study; 176 patients	Age ≥18 years, RA classified according to the criteria of the 2010 American College of Rheumatology (ACR)/European League Against Rheumatism (EULAR), SSc classified according to the criteria of ACR/EULAR 2013, or dermato- myositis and poly-myositis (IM) classified according to the criteria of Bohan and Peter, as applicable.	MTX, leflunomide, sulfasalazine, HCQ, TNFi, tocilizumab, abatacept, RTX, MMF, AZA, CYP, glucocorticoid	≥18 years; male and female	Arterial hypertension, diabetes mellitus, obesity	Pfizer/BioNTech (BNT162b2) or moderna (mRNA-1273); primary	There were 18 COVID-19 cases in 163 fully vaccinated patients: 4 cases after vaccination and 14 cases before fully vaccinated. Of the 13 patients who were not vaccinated, four had severe COVID-19 (three died).	N/A	N/A	No autoimmune relapse was reported. Twenty-four of 163 patients had systemic symtoms after the primary vaccination.
27	Meyer-Arndt, et al (2022)	Germany, single-center	MS (126)	Prospective cohort; 126 patients	MS diagnosis according to the McDonald criteria of 2017; stable disease for at least 3 months (no acute relapse therapy, no clinical progression or new symptoms suggestive of relapse, no disease activity on brain MRI); continuous immunomodulatory treatment or no treatment for at least 3 months; immunomodulatory monotherapy (if treated); no medical contraindications against SARS-CoV-2 vaccination.	no immunosuppressants, aCD20-BCD, fingolimod, β-interferons, DMF, GA, teriflunomide, natalizumab	21-68 years; male and female	No data	BNT162b2 (BioNTech/Pfizer) or mRNA-1273 (Moderna); primary and booster	N/A	IgG titer, seroconversion, neutralizing antibody, B cells count, T cells response	1) Compared with untreated patients with MS, patients with MS receiving aCD20-BCD or fingolimod showed significantly lower or no anti-S1 IgG levels post vaccination<br><br>2) In patients receiving DMTs, except for aCD20-BCD and fingolimod, seroconversion was not significantly different to untreated patients with conversion rates ranging from 67% to 100% after the primary vaccination, depending on the treatment group, and a 100% seroconversion rate after the	N/A

												secondary vaccination.<br><br>3) Untreated MS showed highest neutralising capacity with mean ID titre of 382 at 3 months post vaccination. IFNβ, DMF, GA, TFN, and NTZ did not affect neutralizing activities, while aCD20-BCD and fingolimod lowered neutralizing titer by 35 and 48 fold compared with untreated patients with MS<br><br>4) only small number of patients have RBD-(aCD20-BCD 7%, fingolimod 50%) and S2-specific (aCD20-BCD 14%, fingolimod 50%) B cells after the secondary vaccination, while other treatment groups have similar B cells compared with untreated MS patient<br><br>5) All treatment group (except fingolimod) have increased SI and SII reactive CD4 T cells after primary vaccination, while fingolimod treated have reduced or no SI and SII reactive CD4 T cells	
28	Mitsunaga, et al (2021)	Japan, single-center	Autoimmune (7)	Prospective study; 9 patients	All of Minamata Hospital's staff who received the BNT162b2 vaccine as a part of a routine program	Immunosuppresive drugs in general (not elaborated)	Autoimmune group: 22- 47 years; male and female	COPD, cancer	Pfizer/BioNTech (BNT162b2); primary	N/A	IgG titer	1) IgG anti-SARS-CoV-2 in the prior COVID-19 infection group were higher than control in prevaccination, first antibody (after first vaccination), and second antibody tests (after second vaccination) (p<0.001).<br><br>2) Antibody titer in prior COVID-19 infection group in first antibody test is higher than control group in second antibody test (p<0.001)	There were 6 of 9 and 6 of 8 patients after first and second dose of vaccine who had local symptoms, respectively. There were 9 of 9 and 7 of 8 patients after first systemic symptoms, respectively.
29	Moyon, et al (2022)	France, single center	SLE (136)	Prospective cohort; 126 patients	18 years or older, with a diagnosis of SLE according to the revised American College of Rheumatology (ACR) classification criteria	HCQ, prednisone, MTX, MMF, AZA, BEL	≥18 years; male and female	No data	Pfizer/BioNTech (BNT162b2); primary	N/A	IgG titer, neutralising antibodies, T cell response	1) MMF and MTX uses were associated with lower anti-spike antibody production (β = −78; 95%CI −133 to −22; p=0.007 and β=−122; 95%CI −184 to −61; p<0.001, respectively)<br><br>2) HCQ, steroid, BEL did not impact IgG production<br><br>3) MMF and MTX significantly dropped neutralising activity compared to other treatments (inhibitory dilution 50) (ID50) D614G median(min–max); 111.2 (30–18910) in MMF treated patients vs 90.4 (30–5527) in MTX-treated patients and 684.6 (30–12061) in other patients; p<0.05)<br><br>4) T cell responses were detected in 57% (17/30) of patients who had neutralising antibody titres, but only detected in 10% (1/10) of patients who had non-	Three of 126 patients developed autoimmune relapse after the second dose of vaccine. Local symptoms occurred in 90 cases and 18 cases in 126 patients after the first and second dose of vaccine, respectively. Systemic symptoms occurred in 171 cases and 101 cases in 126 patients after the first and second dose of vaccine, respectively.

												neutralising antibody titres (p<0.05)	
30	Quintanilla-Bordás, et al (2022)	Spain, multi-center	MS (4)	Case series; 4 patients	All patients presented symptoms suggestive of demyelination starting within 60-21 days before the first mRNA vaccine dose, patients received vaccination either before seeking medical attention or while being studied for their symptoms on an outpatient basis.	MP, RTX, CYP, fingolimod, PLEX	16-41 years; male and female	No data	Pfizer/BioNTech (BNT162b2) or moderna (mRNA-1273); primary	N/A	N/A	N/A	All patients developed bilateral ophthalmoplegia, facial palsy, dysarthria, tetraparesis, pyramidalism, global hypoestesia, limb dysmetria, and severe gait ataxia, somnolence, dysarthria, dysphagia, hiccups, severe nausea, and increased right- sided weakness after vaccination.
31	Sakano, et al (2022)	USA, single-center	Autoimmune (3); ILD and AS (1), restrictive lung disease (1), pauci-immune glomerulonephritis (1)	Case series; 3 patients	Patient in their 60s who received rituximab and subsequently developed severe COVID-19 pneumonia after completing vaccination.	prednison, RTX, MMF, nintedanib	63 - 68 years; male and female	No data	Pfizer/BioNTech (BNT162b2) or moderna (mRNA-1273); primary	Three patients had COVID-19 infection after vaccine administration. All of the patients had been hospitalized. One of 3 patients died.	B cells count	Undetectable CD19+/CD20+ after hospitalization for COVID-19	N/A
32	Schwarz, et al (2022)	Germany, single-center	MS (222); relapsing remitting MS (177), primary progressive MS (45)	Prospective observational; 68 patients and 19 healthy controls	Age ≥17 years, a diagnosis of relapsing-remitting MS (RRMS) or primary progressive MS (PPMS) according to the McDonald 2017 criteria, and treatment with at least one intravenous infusion of anti-CD20 therapy (ocrelizumab or rituximab) as part of routine clinical care during the study period	aCD20-BCD	≥17 years; male and female	No data	Pfizer/BioNTech (BNT162b2); primary	N/A	IgG titer, seroconversion, neutralizing antibody, IgG avidity, T cell response	1) Only 26 out of 51 aCD20-BCD pwMS (50.9%, 95% CI: 37.7–64.3) were reactive for anti-SARS-CoV-2-S1 IgG antibodies compared to 13 out of 14 pwMS before aCD20-BCD therapy (92.9%, 95% CI: 68.5–97.6, p = 0.005), and 19 out of 19 HE (100%, 95% CI: 83.2–100, p<0.0001)<br><br>2) IgG levels were lower in aCD20-BCD pwMS (median (IQR) optical density (OD) ratio 1.2 (0.1–5.1) compared to pwMS before aCD20-BCD therapy (9.0 (6.8–9.9) p<0.0001) and to HE (8.8 (8.0–9.4) p<0.0001)<br><br>3) neutralizing capacity of SARS-CoV-2 antibodies was lower in aCD20-BCD-treated pwMS (median (IQR) PRNT50 Titer: 40 (0–80)) than in pwMS before aCD20-BCD therapy (640 (80–640) p = 0.006) and in HE (640 (320–640) p < 0.0001)<br><br>4) Only 4 out of 26 (15.4%, 95% CI: 6.2–33.5) aCD20-BCD -treated pwMS had high anti-SARS-CoV-2-S1 IgG avidity indices compared to 10 out of 13 (76.9%, 95% CI: 49.7–91.8, p = 0.0003) pwMS before aCD20-BCD therapy, and 17 out of 19 hospital employee (85.5%, 95% CI: 68.6–98.1, p<0.0001)<br><br>5) SARS-CoV-2 spike-specific T cell responses were detectable in all aCD20-BCD-treated pwMS (26/26, 100%, 95% CI: 87.1–100), similar to pwMS before aCD20-BCD therapy (7/7, 100%, 95% CI: 64.6–100, p=1), and HE (19/19, 100%,	N/A

												95% CI:<br>83.2–100, p=1)<br><br>6) levels of IFN-γ released by SARS-CoV-2 spike specific T cells were similar between the three groups	
33	Sieiro Santos, et al (2022)	Spain, single-center	IMRD (147); RA (55), SJ (24), SLE (44), SS (24)<br><br>100 patients from cohort 1, (IMRD immunosuppresive + (42 RA, 32 SLE, 12 SJ and 14 SS)<br>47 patients from cohort 2, IMRD immunosuppresive - (13 RA, 12 SJ, 10 SS and 12 SLE)	Longitudinal observational study; 147 patients and 50 healthy controls	IMRD population >18 years from CAULE's rheumatology outpatient clinic	HCQ, CQ, TNFi, IL-6 inhibitor, no immunosuppressants, BEL, MTX, AZA, RTX, MMF	>18 years; male and female	No data	Pfizer/BioNTech (BNT162b2) or moderna (mRNA-1273); primary	N/A	Seroconversion, IgG titer, T cell response	1) All healthy controls (50/50, 100%) demonstrated seroconversion, hereas patients with IMRD ISP− and patients with IMRD ISP+ achieved seroconversion rates of 80% (p=0.03) and 55% (p=0.02), respectively<br><br>2) Median IgG titres for healthy controls were 526.3—IQR 2078, 458.6—IQR 2960 (p=0.18) for cohort 2, and 254—IQR 280 for cohort 1, which averaged an almost twofold reduction in antibody titres in patiets with IMRD ISP+ compared with patients with IMRD ISP− (p=0.0072)<br>3) Complete functional Th1 response consisting of IL-2 and IFN-γ production (a surrogate of Th1 response) was present in all 50 healthy controls (100%), in 35 patients with IMRD ISP− (75%) (p=0.002) and in 52 patients with IMRD ISP+ (52%) (cohort 1 vs cohort 2, p=0.01).<br><br>4) Functional T CD8 responses consisting of S-induced Granzyme A/B detection in supernatants were observed in 46/50 healthy controls (92%), whereas 77% and 53% from IMRD ISP− and IMRD ISP+ showed positive responses (p=0.04 and p=0.01)	N/A
34	Wagner, et al (2022)	Austria, single center	IBD (130)	Prospective cohort; 130 patients and 66 controls	Patients without prior COVID-19 vaccination suffering from solid tumors (SOT), multiple myeloma (MM), inflammatory bowel disease (IBD) as well as healthy individuals (controls)	Steroids, aCD20-BCD, anti-CD38, CYP, JAKi, TNFi, infliximab, adalimumab	19 - 77 years; male and female	No data	Pfizer/BioNTech (BNT162b2) or moderna (mRNA-1273); primary and booster	N/A	IgG titer, neutralizing antibodies, T cell response, leukocyte count, T cells count	1) Lowest GMC titer were in MM patients (GMC=552.5 BAU/ml), compared to IBD (GMC=2275.3 BAU/ml) and controls (GMC=3205.5 BAU/ml; p<0.000), patients with SOT have lower GMC titer (GMC=1529 BAU/ml) compared to controls (GMC=3205.5 BAU/ml; p<0.0001)<br><br>2) After vaccination, GMT were significantly lower in SOT (n=8; GMT=99.9; p=0.0044) and MM patients (n=9; GMT=71.8; p=0.0073) compared to controls (n=25; GMT=453.3)<br><br>3) After the second dose, IBD patients and controls, mounted a clear T cell response upon stimulation with the peptide pool of the S1 subunit. T cells of SOT patients secreted T cell growth factor IL-2, pro-	N/A

												inflammatory cytokines IFN-g, IL-17a and GM-CSF and the regulatory cytokine IL-10, whereas only IFN-g and concomitant IL-17a and IL-10 were induced in MM patients<br><br>4) Patients with MM displayed lower total absolute cell numbers of leukocytes, total lymphocytes, CD3+ T cells and CD3+ CD4+ Thelper cells compared to controls (p=0.0398, p=0.0601, p=0.0342 and p=0.0017, respectively). In regard to CD19+ B cells, patients with SOT (p=0.0381) and MM (p=0.0073) had lower levels than controls<br><br>5) No significant differences were detected for monocytes, granulocytes, CD8+ T cells and NK cells, except for lower granulocyte counts in the MM patients compared to controls (p=0.0109)	
35	Zavala-Flores, et al (2022)	Peru, single-center	SLE (100)	Descriptive observational study; 100 patients	SLE (according to SLICC 2012/CCR/EULAR 2019), older than 18 years, received at least one dose of BNT162b2 vaccine	no immunosuppressants, prednisone, HCQ, AZA, MMF, MTX, leflunomide	>18 years; male and female	Hypothyroidism, chronic kidney disease, APS, arterial, hypertension, rheumatoid, arthritis, sjögren's syndrome	Pfizer/BioNTech (BNT162b2); primary	N/A	N/A	N/A	An autoimmune relapse occurred in 9 cases and 18 cases in 100 patients after first and second dose of vaccine, respectively.
36	Zecca, et al (2022)	Italy, single-center	AIH (22), SS (7), vasculitis (5), RA (32), SpA (29), SLE (11)	Prospective Cohort; 131 patients and 52 healthy subjects	Age > 18 years old; signed informed consent; diagnosis of spondyloarthritis, autoimmune, hepatitis, rheumatoid arthritis, connective tissue disease, vasculitis, liver transplantation and chronic immunosuppressive therapy for patients; absence of a diagnosis of autoimmune disease or liver transplantation and not receiving immunosuppressive therapy for healthy subjects; planned mRNA-based, anti-SARS-CoV-2 primary vaccination cycle (2 doses of BNT162b2 or mRNA- 1273 vaccines administered in clinical practice according to local healthcare policy)	MMF, prednisone, MTX, AZA, HCQ, sulfasalazine, TNFi, IL-6 inhibitor, IL-17 inhibitor, BEL	>18 years; male and female	Coronary artery disease, Hypertension, Neoplasia, Chronic obstructive pulmonary disease, Pulmonary arterial hypertension, Thyroiditis, Diabetes mellitus type II	Pfizer/BioNTec h (BNT162b2) or moderna (mRNA- 1273); primary	N/A	Seroconversion	1) All healthy individuals developed anti-SARS-CoV-2 IgG antibodies; by contrast, only 84.0% of patients showed a detectable antibody response to vaccination at 90 days (p = 0.0010)<br><br>2) In multivariate with only immunosuppressive drug, treatment with MMF were the only independent predictors of vaccination failure (p = 0.0003 and p = 0.0045).<br><br>3) In multivariate with comorbidities and clinical conditions, SLE were independent predictors of vaccination failure (p=0.0032)	N/A
37	Dayam, et al (2022)	Canada, multi-center	IMID (69); IBD (18), psoriasis (22), PsA (12), AS (4)	Observational cohort study; 124 patients and 26 healthy controls	Adult patients with IMID treated with anti-TNF therapies, MTX or AZA monotherapy, combination therapy of MTX/AZA plus anti-TNF therapies, anti–IL-12/23 therapy, anti–IL-23 therapy, or no immunosuppressants.	no immunosuppressants, TNFi, MTX, AZA, IL-23 inhibitor, IL-12/23 inhibitor, IL-17 inhibitor	>18 years; male and female	No data	Pfizer/BioNTec h (BNT162b2) or moderna (mRNA- 1273); primary	N/A	Seroconversion, T cell response	1) SC for RBD and spike IgG after second dose of vaccine in patient group was 99.2% and 100%, respecitively.<br><br>2) Participants on TNFi and TNFi+MTX/AZA showed significantly lower neutralization response to the WT and all variants, as compared with controls or untreated IMID groups<br><br>3) The cytokines IFN-γ, IL-2, IL-17A, and IL-4 were increased over baseline (T1) after 1 or 2 doses of mRNA vaccine in all patient groups (T2 and T3), with the response	N/A

												predominantly of the Th1 phenotype as characterized by high levels of IFN-γ and IL-2	
Inactivated virus vaccine (n=10)													
1	Aikawa, et al (2022)	Brazil, single-center	Patients (942); SARS-CoV-2 seropositive patients with AIRD (157), SARS-CoV-2 seronegative patients with AIRD (471), SARS-CoV-2 seropositive controls (157), SARS-CoV-2 seronegative controls (157)<br><br>RA (164), AxSpA (112), PsA (72), SLE (152), systemic vasculitis (42), SAMS (26), SS (20), SJ (22), APS (17)	Subgroup analysis of phase 4 study; 942 patients	Aged 18 years or older and if they fulfilled the classification criteria for one of the following autoimmune rheumatic diseases: rheumatoid arthritis, systemic lupus erythematosus, spondyloarthritis, vasculitis, primary Sjogren's syndrome, systemic sclerosis, systemic autoimmune myopathies, and primary antiphospholipid syndrome.	HCQ, sulfasalazine, prednisone, MTX, leflunomide, MMF, AZA, CYP, CYC, tacrolimus, tofacitinib, TNFi, abatacept, secukinumab, tocilizumab, RTX, BEL, ustekinumab	≥18 years; male and female	No data	CoronaVac; primary	There were 42 of 628 patients who had COVID-19 breakthrough infections.	Seroconversion, neutralizing antibodies	1) Seropositive patients and controls had similar IgG titres at day 0 (p>0.999) and day 69 (p=0.41) but titres were higher in seropositive controls at day 28 (p=0.0080). For neutralising antibody activity, the values were similar at day 0 (p>0.999), day 28 (p=0.119), and day 69 (p=0.300)<br><br>2) Seropositive patients had significantly higher values than seronegative patients at all timepoints for IgG GMTs and neutralising antibody activity<br><br>3) Seropositive patients also had significantly higher IgG GMTs and neutralising antibody activity than did seronegative controls at all timepoints	Local symptoms occurred in 155 and 114 of 628 patients after the first and second dose of vaccine, respectively. Systemic symptoms occurred in 266 and 206 of 628 patients after first and second dose of vaccine, respectively.
2	Araujo, et al (2022)	Brazil, single-center	RA (92) with low disease activity/remission CDAI <= 10	RCT; 92 patients (37 patients who withdrew MTX after both vaccine dose and 55 patients who maintained MTX)	Adult (≥18 years old) patients with RA diagnosis with low disease activity or remission (CDAI ≤10) at first vaccination day and with stable methotrexate dose for at least 4 weeks, both in monotherapy or in association with synthetic or biologic disease-modifying antirheumatic drugs (DMARD)	prednisone, MTX, leflunomide, abatacept	≥18 years; male and female	No data	CoronaVac; primary	N/A	Seroconversion, neutralizing antibodies	1) At D69, the MTX-hold group (n=37) had a higher rate of seroconversion than the MTX-maintain group (n=55) (29 (78.4%) vs 30 (54.5%), p=0.019), with parallel augmentation in GMT (34.2 (25.2–46.4) vs 16.8 (11.9–23.6), p=0.006).<br><br>2) No differences were observed for NAb positivity (23 (62.2%) vs 27 (49.1%), p=0.217) and NAb activity (p=0.335)	Local symptoms occurred in 13 and 7 of 60 patients after the first and second dose of vaccine, respectively. Systemic symptoms occurred in 27 and 19 of 60 patients after the first and second dose of vaccine, respectively. Similar rate of flares between MTX-hold and MTX-maintain group (DAS28-CRP criteria). More flares in MTX-hold group compared to MTX-maintain group at day 69 (CDAI>10).
3	Chen, et al (2022)	China, multi-center	AIIRD (2921)<br><br>SLE (1116), RA (560), SJ (496)	Observational survey; 183 vaccinated patients	AIIRD patients who had been diagnosed using the latest classification of disease, Chinese citizens, aged >=18 years, able to read and understand Chinese.	N/A	33 - 53 years; male and female	Hypertension, chronic pulmonary disease, diabetes mellitus, coronary heart disease, cerebrovascular disease, cancer	CoronaVac; primary	N/A	N/A	N/A	The main side effects were injection reaction (18.5%), fatigue (15.3%), myalgia (13.1%), arthralgia or arthritis (5.4%), rash (3.8%), and headache (3.8%).
4	Gualano, et al (2022)	Brazil, single-center	AIRD (898) and non-AIRD (197)<br><br>CIA (483) (RA, AxSpA, PsA), other AIRD (415) (SLE, primary vasculitis, SS, SJ, IIM, APS)	Prospective cohort; 898 ARD patients and 197 non-ARD patients	ARD patients aged ≥ 18 years and diagnosed with rheumatoid arthritis, systemic lupus erythematosus, axial spondyloarthritis, psoriatic arthritis, primary vasculitis, primary Sjogren's syndrome, systemic sclerosis, systemic autoimmune myopathies and primary anti- phospholipid syndrome.	prednisone, TNFi, abatacept, tocilizumab, BEL, secukinumab, RTX, ustekinumab, MTX, leflunomide, MMF, AZA, tofacitinib, CYP, tacrolimus, CYC	≥18 years; male and female	Systemic arterial hypertension, diabetes mellitus, dyslipidemia, cardiomyopathy, chronic renal disease, chronic obstructive pulmonary disease, asthma, interstitial lung disease, pulmonary hypertension, hematologic disease, hepatic disease, cancer, stroke, tuberculosis	CoronaVac; primary	N/A	Seroconversion, neutralizing antibodies	1) After vaccination, frequency of seroconversion (P < .001), GMT (P < .001), FI-GMT (P < .001), frequency of NAb (P = .022) and its neutralizing activity (P < .001) were greater in active vs. inactive ARD patients.<br><br>2) Active non-AIRD individuals exhibited greater seroconversion than inactive ones (P = .038).	N/A
5	Huang, et al (2022)	China, single-center	412 pre-vaccination grave disease<br>231 pos- vaccination grave disease	Retrospective and prospective cohort; 643 patients	All grave disease patients in the Endocrinology Department of the Second Affiliated Hospital of Fujian Medical University from January to August 2021. The patients included conformed to the diagnostic and treatment criteria of GD of the	Thiamazole, letrox	Mean age: 39.17 years; male and female	No data	CoronaVac; primary	N/A	N/A	N/A	The changes in serum thyrotropin receptor antibody levels were significantly higher in post- vaccination patients than pre-vaccination patients (p < 0.000).

					European Thyroid Association (ETA) (2018).								
6	Medeiros-Ribeiro, et al (2021)	Brazil, single-center	AIRD (910); CIA (451), RA (256), AxSpA (106), PsA (89), other SARD (459), SLE (232), primary vasculitis (66), SJ (42), SS (41), IIM (41), APS (37)	Prospective controlled clinical trial; 910 patients and 182 controls (safety analysis), 859 patients and 179 controls (immunogenicity analysis)	Patients with ARD and ≥18 years of age from the Outpatient Rheumatology Clinics with the following diagnoses: RA, SLE, axSpA, PsA, primary vasculitis, pSSj, SSc, IIM, and PAPS, none of these were previously vaccinated in the hospital's regular campaign.	prednisone, HCQ, sulfasalazine, MTX, leflunomide, MMF, AZA, tofacitinib, CYP, tacrolimus, CYC, abatecept, tocilizumab, belimumab, secukinumab, RTX, ustekinumab	≥18 years; male and female	Systemic arterial hypertension, diabetes mellitus, dyslipidemia, obesity, chronic cardiomyopathy, chronic renal disease, chronic obstructive pulmonary disease, asthma, interstitial lung disease, pulmonary hypertension, hematologic disease, hepatic cancer, stroke, tuberculosis, chronic inflammatory arthritis, SLE, primary vasculitis, systemic sclerosis, primary sjogren syndrme, idiopathic inflammatory myositis, pulmonary alveolar proteinosis	CoronaVac; primary	There were 35 of 1,193 patients and 3 controls who had been infected by COVID-19 after the vaccine administration.	Seroconversion, IgG titer, neutralizing antibodies	1) Analysis of the SARS-CoV-2 S1/S2 IgG response at D69 revealed a lower seroconversion rate in patients with AIRD (70.4 versus 95.5%, P<0.001).<br><br>2) Mean IgG titers were similar at D0 in both groups (P>0.999) and increased at each time point for ARD and CG (P<0.001). At the D28 and D69 evaluations, patients with ARD presented lower mean titers than CG (P<0.001)<br><br>3) At D28, patients with ARD had lower frequencies (177 (20.6%) versus 65 (36.3%), P<0.001), but with similar median (IQR) NAb activity (42.6% (35.8–60.4) versus 45% (34.5–71.1), P=0.490) compared with CG. At D69, lower median (IQR) neutralization activity (58.7% (43.1–77.2) in ARD, versus 64.5% (48.4–81.4), P=0.013), was observed<br><br>4) prednisone (OR=0.40; 95%CI 0.28–0.56, P<0.001), MTX (OR=0.42; 95%CI 0.29–0.61, P<0.001), MMF (OR=0.15; 95%CI 0.09–0.24, P<0.001), TNFi (OR=0.41; 95%CI 0.26–0.64, P<0.001), abatacept (OR=0.24; 95%CI 0.13–0.46, P<0.001) and RTX (OR=0.34; 95%CI 0.13–0.93, P=0.036) were associated with the absence of seroconversion in patients with ARD<br><br>5) prednisone (OR=0.48; 95%CI 0.35–0.65, P<0.001), MTX (OR=0.67, 95%CI 0.47–0.95, P=0.024), MMF (OR=0.33; 95%CI 0.21–0.53, P<0.001) and RTX (OR=0.28; 95%CI 0.09–0.87, P=0.028) is associated with the absence of neutralizing activity in patients with ARD	There were 213 of 909 and 154 of 893 patients and 36 of 182 and 32 of 181 controls who had local symptoms after first and second dose, respectively. Systemic symptoms occurred in 392 of 909 and 298 of 893 of patients and 61 of 182 and 56 of 181 controls after first and second dose of vaccine, respectively.
7	Seree-aphinan, et al (2022)	Thailand, single-center	Pemphigus (7), psoriasis (6), chronic spontaneous urticaria (1)	Prospective observational case-control; 14 patients with immunosuppressive therapies and 18 controls	Case: Patients with immune-mediated dermatological conditions who had been treated with systemic immunosuppressive agents from 1 month before to 1 month after vaccination were recruited, those whose B cells were depleted (CD19+ lymphocyte < 5%), those whose B cells were incompletely depleted or repopulated after rituximab therapy (CD19+ lymphocyte ≥ 5%) were also defined as cases when an additional immunosuppressant is needed for	AZA, CYC, MMF, prednisolone, MTX, secukinumab, ixekizumab, omalizumab	Mean age --> Case: 43.9 years, control: 44.6 years; male and female	Acne, melasma, androgenetic alopecia, seborrheic keratosis	CoronaVac; primary	No participants developed symptomatic COVID-19 infection (minimum 3-months follow-up period after vaccination).	IgG titer, seroconversion, neutralizing antibodies	1) Anti-SARS-CoV-2 IgG in patient group (666.2 AU/mL) was significantly lower than in the control group (1,208.0) (p = 0.028).<br><br>2) Seroconversion was similar in patients (56.3%) and control group was 77.8% (p=0.180)<br><br>3) Neutralizing activity of sNAb was similar in patients (43.1	One of 14 patients developed an autoimmune relapse. The most common side effects were low-grade fever, myalgia, mild tenderness at the injection site, and somnolence.

					disease control. Controls: Individuals who did not receive systemic immunosuppressive agents were recruited.							%inhibition) and control group (52.9 %inhibition) (p=0.252)<br><br>4) participants using AZA, CYC, MMF, or prednisolone ≥ 10 mg/day had a lower level of serum anti-SARS-CoV-2 IgG antibody and sNAb than those received MTX ≤ 10 mg/week, prednisolone < 10 mg/day, or biologics (i.e., secukinumab, ixekizumab, omalizumab).<br><br>5) Patients using AZA, CYC, MMF, or moderate to-high-dose corticosteroids did not developed seroconversion after vaccination<br><br>6) Patients who received MTX ≤ 10 mg/week, prednisolone <10 mg/day or the biologics had a similar immunogenicity profile to those without immunosuppressive therapies	
8	Shinjo, et al (2022)	Brazil, single-center	SAMs (53)	Prospective controlled study; 53 SAMs patients and 106 subjects in control group (no autoimmune rheumatic disease or other immunosuppres-sive condition and without immunosuppressive therapy)	Patients with SAMs who are 18 years or older, and fulfilled the EULAR/ACR2017 classification criteria for the inflammatory myopathies, and patients with antisynthethase syndrome (ASSD)	prednisone, MMF, MTX, AZA, leflunomide, CYC, CYP	≥18 years; male and female	Systemic arterial hypertension, diabetes mellitus, dyslipidaemia, obesity, myocardial infarction, interstitial lung disease, stroke	CoronaVac; primary	Three symptomatic COVID-19 cases among systemic autoimmune myopathies and 3 cases in control group after vaccination. All participants had mild symptoms and none required hospitalization.	Seroconversion, IgG titer, neutralizing antibodies= 0.808). SC in the patient group was 64.9%, whereas in the control group was 91.1%. Patients with NAb positivity used less often immunosuppressive drugs than those without	1) at D69, a moderate but significantly lower SC (64.9% vs 91.1%, P<0.001), GMT [7.9 (95%CI 4.7–13.2) vs 24.7 (95%CI 30.0–30.5) UA/ml, P<0.001] and frequency of NAb (51.4% vs 77.2%, P<0.001) in SAMs compared with CG.<br><br>2) Median neutralizing activity was comparable in both groups after the first [39.2 (38.4–52.5) vs 46.6 (36.9–73.3), P = 0.573] and second dose [57.2 (43.4–83.4) vs 63.0 (40.3–80.7), P = 0.808]<br><br>3) Patients with NAb positivity used less often immunosuppressive drugs than those without NAb (73.7% vs 100%, P = 0.046).	There were 11 of 53 and 11 of 50 patients and 18 and 19 of 50 controls who had local symptoms after the first and second dose, respectively. Systemic symptoms occurred in 23 of 53 and 16 of 50 of patients and 34 and 31 of 106 controls after first and second dose of vaccine.
9	Yuki, et al (2022)	Brazil, single-center	SLE (232)	Prospective controlled trial; 232 patients and 58 controls	Case: consecutive SLE patients who fulfilled SLE Collaborating Clinic (SLICC) classification criteria (22), ≥18 years and followed up at the outpatient SLE clinics. Control: haven't immunized in the hospital's COVID-19 campaign	HCQ, prednisone, MMF, AZA, MTX, calcineurin inhibitor, CYP, leflunomide, BEL	Median age: 26 years; male and female	Pulmonary arterial hypertension, DM, dyslipidemia, obesity, chronic cardiomyopathy, chronic renal disease, smoking, chronic obstructive pulmonary disease, asthma, interstitial lung disease, pulmonary hypertension, hematologic disease, hepatic disease, current cancer, stroke, TBC, HIV	CoronaVac; primary	Evaluation period for incident cases was 80 days since D0. Eleven symptomatic cases of COVID-19: 9 SLE patients and 2 controls (all occurred before two weeks of completed vaccination). One SLE patient required hospitalization but did not require mechanical ventilation or ICU admission and no patient died.	Seroconversion, neutralizing antibodies	1) At D69, SLE patients showed a moderate seroconversion (70.2% versus 98.1%; P < 0.001) and moderate frequency of NAb positivity (61.5% versus 84.6%; P = 0.002), although both frequencies were lower than in controls<br><br>2) In multivariate analysis, prednisone and MMF use were independently associated with lower seroconversion (P < 0.001) and NAb positivity (P < 0.001)	There were 71 and 62 of 223 patients and 12 and 18 of 56 controls who had local symptoms after the first and second dose, respectively. Systemic symptoms occurred in 109 and 82 of 223 patients and 23 and 24 of 56 controls after the first and second dose of vaccine, respectively. After fully vaccination, 4.7% of the SLE patients reported disease exacerbation. No worsening of SLEDAI-2K score up to 3 months after full vaccination in 118 SLE patients.
10	Balcells, et al (2022)	Chile, multi-center	RA (31), PsA (9), JIA (1)	Prospective cohort study; 41 patients and 65 healthy controls	Adult patients with pre-defined acquired immunosuppressive; having received two doses of CoronaVac vaccine separated by 4 weeks with the second dose administered 8-12 weeks before enrollment. Specific inclusion criteria for each cohort: 1. Cancer: diagnosis of solid tumor (excludes leukemias, lymphomas, or multiple myelomas) and currently receiving	prednisone, HCQ, sulfasalazine, leflunomide, MTX, MMF, tacrolimus, CYC, TNFi (infliximab, golimumab, adalimumab, etanercept, certolizumab), IL-6 inhibitor (tocilizumab), IL-17 inhibitor (secukinumab)	Mean age --> Control: 44.3 years, rheumatic: 51.7 years; male and female	Hypertension, diabetes, asthma	CoronaVac; primary	Four cases (1.5%) of non-severe breakthrough infections but no information whether from autoimmune group or other immunocompromised patients.	IgG titer, neutralizing antibodies, T cell response	1) NAb positivity and median neutralizing activity were 83.1% and 51.2% for the control group versus 20.6% (p<0.0001) and 5.7% (p<0.0001) in the SOT) group, 41.5% (p<0.0001) and 19.2% (p<0.0001) in the AIRD group, 43.3% (p=0.0002) and 21.4% (p=0.0013) in the cancer	N/A

					chemotherapy. 2. HSCT: allogeneic with active immunosuppressive treatment or autologous transplantation, in the last 5 years. 3. SOT: liver, kidney or heart transplant in the last 5 years, and active immunosuppressive treatment. 4. HIV: HIV infection under antiretroviral therapy with CD4+ cell count ≤ 500 cells/mm3 and HIV viral load <200 copies/ml. 5. Autoimmune rheumatic diseases: rheumatoid arthritis, psoriatic arthritis, ankylosing spondylitis, relapsing polychondritis, Behcet's disease or juvenile idiopathic arthritis, receiving chronic immunomodulatory treatment with anti-TNF, anti-IL6 or anti-IL17 agents.							patients with SOT group, 45.5% (p<0.0001) and 28.7% (p=0.0006) in the HIV infected group, 64.3% (p=n.s.) and 56.6% (p=n.s.) in the HSCT group, respectively.<br><br>2) TAb seropositivity was also lower for the SOT (20.6%, p<0.0001), rheumatic diseases (61%, p=0.0001) and HIV groups (70.9%, p=0.0032), compared to control group (92.3%)<br><br>3) IFN-γ Spot Forming T Cells specific for SARS-CoV-2 tended to be lower but did not differ significantly between groups.	
Adenovirus vector vaccine (n=2)													
1	Shenoy, et al (2021)	India, single-center	AIRD (120)<br><br>single dose vaccine n=30<br>COVID infection only n=30<br>double dose vaccine n=30<br>infection + single dose vaccine n =30<br><br>RA (81)<br>SpA (10)<br>SLE (10)<br>Others (19)	Cohort; 120 patients	Cases: patients who had SARS-CoV-2 infection in past 12 months and had received one dose of ChAdOx1 vaccine (infection plus vaccine).	MTX, HCQ, sulfasalazine, leflunomide, MMF, AZA, corticosteroid	≥18 years; male and female	No data	Vaxzevria (ChAdOx1); primary	N/A	IgG titer, seroconversion, neutralizing antibodies	1) The infection plus vaccine group had the highest antibody titre compared with all other groups (p<0.0001)<br><br>2) There was 100% seroconversion in the infection plus vaccine group, compared with 90% in the double dose vaccine group (p<0.0001)<br><br>3) The infection plus vaccine group also had higher neutralisation capacity (87% of individuals with at least 30% neutralisation) compared with the double dose vaccine group (60%;p=0.039).	N/A
2	Mehta, et al (2022)	India, single-center	AIRD (495), RA (332), SpA (86), SLE (39), vasculitis (17), CTD (10), SS (11)	Cohort; 495 patients	Vaccinated patients with AIRDs are being followed at Centre for Arthritis and Rheumatism (CARE), patients who were administered AZD1222	MTX, HCQ, sulfasalazine, leflunomide, tofacitinib, MMF, tacrolimus, AZA, RTX, TNFi, glucocorticoid	Mean age: 56.5 years; male and female	Diabetes mellitus, hypertension	astrazeneca (AZD1222); primary	Breakthrough COVID-19 infections were reported in survival analysis, but no clear explanation about the infections number. The infections were higher in patients received the second dose of vaccine at 4 weeks than patients received the second dose of vaccine after 10-14 weeks, although the difference was not significance (p=0.25).	IgG titer	1) Group 2 (vaccine interval 10-14 weeks) had higher anti-RBD antibody titre [1310.6 (±977.8)] IU/ml as compared to Group 1 (Vaccine interval 4-6 weeks) [736 (±864.7), p= 0.0001].<br><br>2) An adequate response was seen in a greater proportion of Group 2 than of Group 1<br><br>3) RA and steroid use as predictors of anti-RBD antibody titres	N/A
mRNA vaccine and adenovirus vector vaccine (n=20)													
1	Arnold, et al (2022)	Germany, single-center	Renal immune disease (34); AAV (22), FSGS (1), MGN (3), MCD (3), Goodpasture Disease (4), Thrombotic Microangiopathy (4)	Retrospective observational study; 34 patients	Age ≥ 18 years, current treatment with the anti- CD20 antibody Rituximab for renal autoimmune disease, and completed base immunization with either mRNA-based (BNT162b2, mRNA-1273), or vector-based (ChAdOx1, Ad26.COV2.S) SARS- CoV-2 vaccines. In vaccination regimens requiring two doses for base immunization, the second dose was given between 2 and 12 weeks after the first vaccination.	aCD20-BCD, corticosteroid, HCQ	≥18 years; male and female	ANCA-associates vasculitis, focal segmental glomerulosclerosis, membranous glomerulopathy, thrombotic microangiopathy	Pfizer/BioNTech (BNT162b2) or moderna (mRNA-1273), or Vaxzevria (ChAdOx1), or Janssen (Ad26.COV2.S); primary	N/A	IgG titer, seroconversion, T cells count, T cell response	1) patients receiving regular aCD20-BCD treatment (every 6 months) had signifcantly anti-SARS-CoV-2-S1 IgG levels<br><br>2) Humoral response was detected in 11 (32.4%) patients<br><br>3) patients receiving regular aCD20-BCD treatment (every 6 months) had signifcantly lower	N/A

												CD19 counts<br><br>4) Cellular response was detected in 23 (92%) patients	
2	Bakasis, et al (2022)	Greece, multi-center	328 SARD; systemic vasculitis or CTD (SLE, APS, SJ, SS, IIM, MCTD, UCTD) (185), inflammatory arthritis (RA, SpA, JIA) (125), other immune-mediated diseases (retroperitoneal fibrosis, Still's disease, relapsing polychondritis, and periodic fever syndromes) (18)	Cohort; 328 patients	Patients aged ≥16 years, vaccinated cases with SAARD infected with SARS-CoV-2	Corticosteroids, colchicine, HCQ, MMF, MTX, AZA, leflunomide, CYC, thalidomide, CYP, TNFi, IL-1 inhibitor, IL-6 inhibitor, IL-17 inhibitor, IL-12/23 inhibitor, BEL, RTX, JAKi	17 - 86 years; male and female	Arterial hypertension, cardiovascular disease, dyslipidemia, diabetes mellitus, chronic lung disease, obesity	Pfizer/BioNTech (BNT162b2) or moderna (mRNA- 1273), or Vaxzevria (ChAdOx1); primary and booster	There were 194 vaccinated patients and 134 unvaccinated patient who had COVID-19 breakthrough infections after vaccination. Seventy-seven and 12 primary vaccinated patients had asymptomatic and severe infections, respectively. One hundred-one and 4 booster vaccinated patients had asymptomatic and severe cases, respectively. One hundred-nine and 25 unvaccinated patients had asymptomatic and severe cases, respectively. Of those who had been infected, 13 of 89, 5 of 105, and 36 of 134 primary, booster, and unvaccinated patients were hospitalized, respectively. Two patients died after received primary vaccination and 3 unvaccinated patients died, both due to COVID-19.	N/A	N/A	There were 319 cases of 89 patients and 314 cases of 105 patients who had systemic symptoms after primary and booster vaccination, respectively.
3	Duengelhoef, et al (2022)	Germany, single-center	AIH (11), PBC (70), PSC (74)	Prospective cohort; 217 patients, and 95 controls	Non-pregnant patients ≥18 years with diagnosed AIH, PSC, or PBC presenting at the YAEL outpatient clinic of the University Medical Center Hamburg-Eppendorf (UKE) for routine visits between July and September 2021	steroids, AZA, MMF	≥18 years; male and female	Cirrhosis, diabetes, arterial hypertension	Pfizer/BioNTech (BNT162b2) or moderna (mRNA-1273), or Vaxzevria (ChAdOx1); primary	N/A	IgG titer, seroconversion,T cell response, B cells count	1) In AIH, almost all patients (91/94%, 97%) achieved seroconversion. Nevertheless, antibody titers were significantly lower in comparison to HC when measured by the Trimer assay (p = 0.001) and tended to be lower when the RBD assay was used (p = 0.08)<br><br>2) Antibody titers in AIH were significantly lower when compared to patients with cholestatic liver diseases<br><br>3) AIH patients without immunosuppression had comparably low antibody levels as those under immunosuppression as well as lower antibody titers than HC (p = 0.050)<br><br>4) Antibody levels of AIH patients were still significantly lower compared to PSC/PBC patients (anti-S Trimer: 669 BAU/ml (IQR 227 vs. 1480) versus 1020 BAU/ml (480–1610), p = 0.027; anti-S RBD: 1142 AU/ml(301–2670) versus 1705 AU/ml (875–2823), p = 0.023)<br><br>5) In the AIM assay of 20 AIH patients, a spike-specific T-cell response was undetectable in 45% despite a positive serology, while 87% (13/15) of the PBC/PSC demonstrated a spike-specific T-cell response	N/A

												6) AIH patients had more immunosuppressive treatment (90% vs. 25%, p < 0.001) along with reduced B-cell counts (54/µL vs. 187/µL, p = 0.001)<br><br>7) Frequencies of spike-specific CD4+ and CD8+ T cells tended to be lower in AIH patients as compared to HC (p = 0.14, and p = 0.05, respectively)	
4	Efe, et al (2022)	Turkey, single-center	AIH (413) with COVID-19	Retrospective cohort; 413 patients	All participants identified patients and collected data from electronic records and patient charts using the same case report form, AIH patients (>16 years old) who were diagnosed with COVID-19 (hospital-based PCR test) between March 11, 2020 and April 01, 2022	AZA, prednisolone, MMF, tacrolimus, MTX	17 - 85 years; male and female	Arterial hypertension, diabetes mellitus, cardiac disease, respiratory disease, kidney sufficinecy, active cancer	Pfizer/BioNTech (BNT162b2) or moderna (mRNA-1273), or Vaxzevria (ChAdOx1); primary	Nineteen and 3 patients had been hospitalized after primary and booster vaccination, respectively. One case died after primary vaccination due to breakthrough infections.	N/A	N/A	N/A
5	Epstein, et al (2022)	USA, multi-center	NID (1164)	Cross-sectional; 1,164 patients with NID and 595 neurologically asymptomatic controls patients	18 years or older with a diagnosis of a neuroinflammatory disease or were part of the neurologically asymptomatic control group.	No immunosuppresants, aCD20-BCD, β-interferons, GA, natalizumab, DMF, S1PRM, teriflunomide, cladribine, alemtuzumab	Mean age--> MS/NID: 52 years, control: 47 years; male and female	Cardiovascular, diabetes, pulmonary, GI/hepatobiliary, renal, malignancy, connective tissue disease	Pfizer/BioNTech (BNT162b2) or moderna (mRNA-1273), or Janssen (Ad26.COV2. S); primary	N/A	N/A	N/A	Systemic symptoms occurred in 256 of 606 and 246 of 367 patients and 141 of 311 and 114 of 176 controls after the first and second dose of vaccine, respectively.
6	Gerosa, et al (2022)	Italy, multi-center	SLE (452)	Retrospective observational; 452 patients	All adult patients (≥18 years of age) referring to participant centres with a previous diagnosis of SLE who had received anti-SARS-CoV-2 vaccines between 29 December 2020 and 31 October 2021 (BNT162b2 mRNA COVID-19 vaccine by Pfizer or mRNA-1273 by Moderna Biotech or ChAdOx1-S by AstraZeneca)	prednisone, HCQ, CYC, RTX, AZA, MMF, BEL, CYP, leflunomide, adalimumab, tocilizumab, tacrolimus	≥18 years; male and female	Musculosceletal involvement, mucocutaneus invlovement, renal involvement, neuropsychiatric involvement, cardiopulmonory involvement, haematological involvement, gastrointestinal involvement, opthalmic involvement, secondary anti-phospholipid syndrome	Pfizer/BioNTech (BNT162b2) or moderna (mRNA-1273), or Vaxzevria (ChAdOx1); primary	There were 77 of 452 patients who had been infected by COVID-19.	N/A	N/A	There was no autoimmune relapse in patients after the first dose of vaccine, yet 19 of 452 patients developed an autoimmune relapse after the second dose of vaccine, respectively. Seventeen of 452 patients had local symptoms after each of the first and second dose of vaccine. Systemic symptoms occurred in 87 cases and 134 cases in 452 patients after the first and second dose of vaccine, respectively.
7	Kennedy, et al (2021)	UK, multi-center	IBD (865)	Prospesctive cohort; 865 infliximab-treated patients and 428 vedolizumab-treated patients	Age 5 years and over, diagnosis of IBD, current treatment with infliximab or vedolizumab for 6 weeks or more with at least one dose of drug received in the past 16 weeks.	Infliximab, vedolizumab	≥5 years; male and female	Heart disease, diabetes, lung disease, kidney disease, cancer	Pfizer/BioNTech (BNT162b2) or Vaxzevria (ChAdOx1); primary	N/A	IgG titer, seroconversion	1) Lowest rates of seroconversion were observed in patients treated with infliximab that received BNT162b2 and ChAdOx1 vaccine were 27.1% and 20.2%, respectively, whereas highest rates of seroconversion were found in patients treated with vedolizumab were 74.7% and 57.3%, respectively.<br><br>2) Anti-SARS-CoV-2 antibody concentrations were lower in infliximab-treated patients compared with vedolizumab-treated patients in participants who received the BNT162b2 (FC 0.29 (95% CI 0.21 to 0.40), p<0.0001) and ChAdOx1 nCoV-19 (FC 0.39 (95% CI 0.30 to 0.51), p<0.0001) vaccines<br><br>3) Immunomodulator use were associated with lower anti-SARS-CoV-2 antibody concentrations, irrespective of vaccine type.	N/A

												4) Following second-vaccine doses, 85% (17/20) infliximab-treated patients and 86% (6/7) vedolizumab-treated patients seroconverted (p=0.68)	
8	Kim, et al (2022)	Korea, single-center	AIRD (149); SLE (43), RA (62), AS (11), Behcet's Disease (10), adult-onset Still's disease (6), others (17)	Cohort; 148 ARD patients and 94 healthcare workers	Aged 18 years or older, had been vaccinated with mRNA (BNT162b2 and mRNA1273) or viral vector (AZD1222 and Ad26.COV2.S) vaccines, patients with ARDs (including systemic lupus erythematosus (SLE), rheumatoid arthritis (RA), ankylosing spondylitis (AS), Behçet's disease (BD), adult-onset Still's disease (AOSD), antineutrophil cytoplasmic antibody-associated vasculitis, systemic sclerosis, IgG4-related disease), had received a second or third dose of COVID-19 vaccine at least 3 weeks prior.	corticosteroid, HCQ, MTX, leflunomide, sulfasalazine, MMF, calcineurin inhibitor, AZA, cyclophosphamide, JAKi, TNFi, tocilizumab, BEL	≥18 years; male and female	Asthma, cancer, cardiovascular disease, diabetes, thyroid disorder	Pfizer/BioNTech (BNT162b2) and moderna (mRNA-1273), or Vaxzevria (ChAdOx1) and Janssen (Ad26.COV2. S); primary and booster	There were 19 of 99 patients and 31 of 94 controls who had been infected by COVID-19. Of those who had been infected, 2 patients were hospitalized.	Neutralizing antibodies, seroconversion, T cell responses	1) Two doses of COVID-19 vaccines induced strong neutralising responses against the wild-type virus in both HCWs and patients with AIRD (72.1% and 76.2%, respectively; p=0.329)<br><br>2) Mean neutralising response against the Omicron variant was 18.1% in HCWs and 11.5% in patients with AIRD (p=0.007).<br><br>3) Seropositivity rate regarding the ancestral anti-spike IgG (≥1.1 AU/ml) was 94.8% and 87.2% after the second dose in HCWs and patients with AIRD, respectively, which increased to 100% and 96.1% after the third dose<br><br>4) Released IFN-γ levels in response to spike-based antigens declined slightly from a median of 324 mIU/mL (IQR 118–555) in HCWs to 203 IU/mL(IQR, 37.5–470) in patients with AIRD, but the difference was not significant (p=0.262)	N/A
9	Krasselt, et al (2022)	Germany, single-center	AIRD (303); RA (127), SpA/PsA (75), CTD (71), SLE (57), AAV (17), LVV (7), Adult-onset still's disease (3), JIA (2)	Cohort; 303 patients	Adult patients with various autoimmune inflammatory rheumatic diseases, adult patients with various autoimmune inflammatory rheumatic diseases	MTX, AZA, MMF, abatacept, BEL, glucocorticoids, IL-17 inhibitor, JAKi, RTX, TNFi, tocilizumab	Mean age: 61.4 years; male and female	No data	Pfizer/BioNTech (BNT162b2), moderna (mRNA-1273), Vaxzevria (ChAdOx1), Janssen (Ad26.COV2. S); primary	N/A	IgG titer, seroconversion, T cell response, B cells count	1) Seroconversion rate was significantly lower in patients under immunosuppressive therapy than in patients without [75.7 vs 93.2%, odds ratio (OR) 0.228, 95% CI 0.068, 0.760, P = 0.009]<br><br>2) Anti-S IgG levels were higher in patients without DMARDs therapy (P = 0.0048)<br><br>3) Sixty-five patients (21.5%) did not mount a humoral response after valid vaccination. T-cell response was assessed in 20 of these patients.<br><br>4) In total, 50% of the patients had a detectable T-cell response towards the SARS-CoV-2 spike protein. The rate of T-cell response was highest in patients under RTX treatment (55.6% vs 33.3% in patients with ABA, MMF or TNFi therapy).<br><br>5) Peripheral B-cell count was higher in patients without T-cell response (20.5 vs 4 cells/cl, P = 0.7863).<br><br>6) Cellular immunity was	N/A

												impaired by the use of glucocorticoids (OR 0.062, 95% CI 0.003, 1.329, P = 0.0379)	
10	Lee, et al (2022)	UK and US, multi-center	iTTP (117)	Retrospective cohort; 77 patients (VAERS data), 117 patients (multicenter cohort), 122 patients (Platelet Disorder Support Association Survey), 311 patients (UK ITP association survey)	Immune thrombocytopenia, thrombocytopenia, decreased platelet count, immunoglobulin (IVIG) therapy, and platelet transfusion, adults with ITP who received a SARS-CoV-2 vaccine between December 2020 and March 2021 and who had a postvaccination platelet count	corticosteroid, IVIG, RTX, vincristine, MMF, ibrutinib	Mean age: 63 and 62.5 years for each cohort; male and female	Any autoimmune disease, hypotiroidism, rheumatologic, dermatologic, gastrointestinal, antiphospolipid syndrome, multiple sclerosis	Pfizer/BioNTech (BNT162b2), moderna (mRNA-1273), Vaxzevria (ChAdOx1), Janssen (Ad26.COV2. S); primary	N/A	N/A	N/A	There were 135 cases of 77 patients that developed thrombocytopenia, presented skin or mucosal bleeding, presented genitourinary bleeding, presented gastrointestinal bleeding, presented central nervous bleeding after vaccination. One death due to central nervous system bleeding was reported.
11	Lin, et al (2022)	Taiwan, multi-center	Total population (399)<br><br>autoimmune diseases as comorbidities (16) (AS, APS, autoimmune thyroiditis, RA, SpA, SJ, SLE)	Randomized controlled trial; 16	Aged 20-65 years, being generally healthy or with stable pre-existing health conditions, having prime vaccinated with either ChAdOx1 or mRNA- 1273, and being scheduled for booster doses of COVID-19 vaccination.	HCQ, sulfasalazine, MTX, steroid, non COX-selective NSAIDs, and COX-selective NSAIDs	20 - 65 years; male and female	No data	Moderna (mRNA- 1273), Vaxzevria (ChAdOx1); primary and booster	N/A	IgG titer	1) Compared with healthy participants, participants with immunocompromising conditions (i.e. those aged >50 years, having comorbidities, or using immunosuppressants and/or immunomodulators) had similar anti-SARS-CoV-2 spike IgG titers before booster vaccination (geometric means, 75.36 vs. 82.87 BAU/mL; P = 0.429)<br><br>2) Lower anti-SARS-CoV-2 spike IgG titers before booster vaccination were found in participants with autoimmune diseases compared to those without (geometric means, 34.76 vs. 84.25 BAU/mL; P = 0.173)<br><br>3) Participants receiving immunosuppressants and/or immunomodulators had significant lower anti-SARS-CoV-2 spike IgG titers before booster vaccination than those without (geometric means, 36.39 vs. 83.84 BAU/mL; P = 0.001)<br><br>4) The participants receiving NSAIDs also had statistically significantly lower anti-SARS-CoV-2 spike IgG titers before booster vaccination compared with those not receiving (geometric means, 39.04 vs. 83.15 BAU/mL; P = 0.007)<br><br>5) Compared with healthy participants, participants with immunocompromising conditions had similar anti-SARS-CoV-2 spike IgG titers 4 weeks after booster vaccination (geometric means, 1769.66 vs. 1946.41 BAU/mL; P = 0.255).<br><br>6) Only participants with autoimmune diseases and receiving HCQ, low-dose steroid, MTX, and/or sulfasalazine had numerically lower anti-SARS-	N/A

												CoV-2 spike IgG titers 4 weeks after booster vaccination compared to those without (geometric means 1474.34 vs. 1923.23 and 1590.61 vs. 1918.38 BAU/mL; both P > 0.05).<br><br>7) Anti-SARS-CoV-2 spike IgG titers 4 weeks after booster vaccination were comparable between participants receiving and not receiving NSAIDs (geometric means, 1894.94 vs. 1903.09 BAU/mL, P = 0.981)	
12	Lonati, et al (2022)	Italy, single-center	aPL positive patients with SAIRD (126); APS (71), aPL-positive asymptomatic carriers (37), APS associated with SAIRD (18)<br><br>aPL negative patients with SLE (50)	Prospective cohort; 126 patients	Case: All samples displayed double or triple positivity for the APS laboratory classification criteria, and medium/high titres of anticardiolipin and anti-beta2 glycoprotein I IgG/IgM. Control: COVID-19 with moderate disease, individuals with non-COVID-19 infections, aPL-negative patients with systemic lupus erythematosus (SLE).	N/A	No data; male and female	No data	Pfizer/BioNTech (BNT162b2), moderna (mRNA-1273), Vaxzevria (ChAdOx1), Sputnik; primary	N/A	N/A	N/A	Local and systemic symptoms occurred in 27 cases among 44 patients and 75 cases among 44 patients, respectively. No other severe adverse event was reported.
13	Pinte, et al (2021)	Romania, multi-center	AID-IMD (623); immune pulmonary involvement (65), AIRD (395), SLE (97), SJ/sicca (78), AS (65), PsA/psoriasis (53), SS/limited scleroderma (31), APS (25), systemic vasculitis (17), other AIRD (15) (dermatomyositis/polymyositis and MCTD), non-AIRD (228), IBD (44), celiac disease (19), PBC (9), AIH (11), MG (28), MS (25), cutaneous disease (vitiligo, cutaneous lupus, cutanous vasculitis, pemphigus) (17), autoimmune thyroid disease (137), other non-AIRD (50) (sarcoidosis, type I DM, hyper IgD syndrome)	Prospective cohort; 623 patients (416 vaccinated and 207 non-vaccinated)	Enrolling patients on the basis of a questionnaire both online and offline.	corticosteroids, HCQ, MTX, sulfasalazine, leflunomide, MMF, AZA	Median age in vaccinated group: 50 years, median age in non-vaccinated group: 48 years; male and female	AIRD, RA, SLE, sjogren syndrome, ankylosing spondylitis, psoriatic arthritis, systemic sclerosis, antiphospholipid syndrome, systemic vasculitis, inflammatory bowel disease, celiac disease, primary billiary cholangitis, autoimmune hepatitis, myasthenia gravis, multiple sclerosis, hematological disease, cutaneous disease, autoimmune thyroid disease	Comirnaty (BioNTech/Pfizer), Vaxzevria (ChAdOx1), Spikevax (Moderna Biotech), and Janssen (Ad26.COV2.S) ; primary	There were 10 of 416 patients who had breakthrough COVID-19 infections.	N/A	N/A	There were 307 of 416 patients who developed an autoimmune relapse after vaccination. Local symptoms occurred in 128 cases in 173 patients and 93 cases in 148 patients after first and second dose of vaccine, respectively. Systemic symptoms occurred in 241 cases of 173 patients and 208 cases of 148 patients after the first and second dose of vaccine, respectively.
14	Prendecki, et al (2021)	UK, single-center	AAV and anti-GBM disease (45), MCD/FSGS (28), MGN (23), SLE (19), Other (C3 glomerulopathy and IgG4-related disease) (4)	Cohort; 140 patients with immune-mediated glomerulo nephritis and vasculitis and 70 healthy volunteers	Patients with immune-mediated glomerulonephritis and vasculitis who received their first-dose of SARS-CoV-2 vaccination (BNT162b2 mRNA or ChAdOx1 nCoV-19) between 17 January 2021 and 9 March 2021.	RTX, tacrolimus, AZA, MMF, MTX, prednisolone, BEL, CYP, no immunosuppressants	Median age on first dose group based on non- seroconversion and seroconversion group: 54.9 years and 49.4 years, median age on second dose group based on non-seroconversion and seroconversion group: 65.1 and 51.9 years; male and female	Diabetes, asthma/COPD, previous malignancy	Pfizer/BioNTech (BNT162b2), Vaxzevria (ChAdOx1); primary	N/A	Seroconversion, IgG titer, T cell response	1) At 18–29 days after second-dose vaccine, the proportion of patients with detectable anti-S IgG increased to 59.4% (54/91). In contrast, all HV individuals had detectable anti-S IgG after second-dose vaccine.<br><br>2) The median anti-S titre after second-dose vaccine was significantly lower in IS patients than in HV, with median 58.7 (IQR 0.8–437.2), median 189.3 (IQR 7.9–1090)<br><br>3) Prior rituximab treatment and current B-cell depletion were associated with a decreased likelihood of	N/A

												seroconversion and median 877 (IQR 575–2203) BAU/mL for IS total cohort, IS matched group, and HV, respectively; p<0.0001).<br><br>4) Comparing the HV and IS group, there was no significant difference in the proportion with T-cell responses to second-dose vaccine (74.4% (32/43) of HV had T-cell responses above threshold) or in the magnitude of response (median 130 and 86 SFU/106 PBMC for IS and HV, respectively; p=not significant (ns))	
15	Shields, et al (2022)	UK, single-center	Rheumatology cohort (36); RA (33), AAV (2), RA/SLE overlap (1)	Cohort; 116 patients	Patients undergoing routine lymphocyte immunophenotyping for the purposes of monitoring B-cell reconstitution following exposure to B-cell-depleting agents (i.e. rituximab or obinutuzumab) to treat underlying haemato-oncological or rheumatological disease.	MTX, sulfasalazine, HCQ, leflunomide, prednisolone	Median age: 69 years; male and female	No data	Pfizer/BioNTech (BNT162b2), Vaxzevria (ChAdOx1); primary	N/A	IgG titer, IgA titer, IgM titer, lymphocyte count, T cells count, B cells count	1) Seropositivity following vaccination in patients were 62.9% and the median magnitude of the antibody response, quantified by the IgGAM ratio in seropositive individuals, was 2.98.<br><br>2) In a cohort of healthy, age-matched controls seropositivity following vaccination was 97.2% with a median IgGAM ratio in seropositive individuals of 4.87.<br><br>3) There were no significant differences in total lymphocytes, CD3+-positive lymphocytes, CD4+-positive lymphocytes, or CD8+-positive lymphocytes between individuals mounting a serological response to vaccine in either patient group.<br><br>4) In both haemato-oncology and rheumatology patients, the size of the CD19+ B-cell population was significantly smaller in patients who had not responded to the vaccine	N/A
16	Stalman, et al (2022)	Netherlands, multi-center	IMID (1593); rheumatological diseases (528) [RA (234), SpA (100), SLE (153), other rheumatological diseases (95) [vasculitis (small-vessel, medium-vessel and large-vessel vasculitis and other forms of vasculitis except giant cell arteritis), other rheumatological (giant-cell arteritis, polymyalgia rheumatica and others)]; neurological diseases (447) [MS, NMOSD,	Prospective cohort; 1,593 patients diseases and 579 controls	Primary immunizations with either two doses of BNT162b2, CX-024414, or ChAdOx1 nCoV-19, or one dose of Ad.26.COV2.S. Patients with SARS- CoV-2 infection after first vaccination who had received only one dose of any of the above.	Other immunosupressants, MTX, TNFi, aCD20-BCD, MMF, S1PRM	Median age --> IMID patients on immunosuppressants: 51 years, controls: 52 years; male and female	Cardiovascular disease, chronic pulmonary disease, diabetes, obesity	Pfizer/BioNTech (BNT162b2), moderna (CX-024414), Vaxzevria (ChAdOx1), Janssen (Ad26.COV2.S); primary	There were 472 of 1,593 patients and 181 of 579 controls who had COVID-19 breakthrough infection. Of them, there were 6 asymptomatic COVID-19 cases among patients compared to 5 of 181 controls. Besides, there were 464 mild symptomatic COVID-19 cases among patients compared to 175 of 181 controls. Two patients were hospitalized.	Seroconversion	1) A total of 1746/1961 (89.0%) of all participants reached seroconversion after primary immunisation<br><br>2) Patients with IMID on strongly antibody-impairing immunosuppressants reached seroconversion in 150/314 (47.8%), while 1100/1143 (96.2%) in patients with IMID on other immunosuppressants and 496/504 (98.4%) in controls reached seroconversion.	N/A

			Inflammatory neuropathies and myopathies (chronic inflammatory demyelinating polyneuropathy, multifocal motor neuropathy and inflammatory myositis), MG)]; gastroenterological diseases (373) [Crohn's disease, ulcerative colitis, AIH, other inflammatory bowel disorders (PBC, PSC)]; dermatological disease (191) [Atopic dermatitis, psoriasis, pemphigus, other dermatological (vitiligo, pemphigus, psoriasis and others)										
17	Stefanski, et al (2022)	Germany, single-center	RA (28), AAV (3)	Prospective Cohort; 19 patients receiving RTX, 12 patients treated with other medicine, and 30 healthy patients	Outpatients with rheumatic disease treated with RTX who received SARS–CoV-2 vaccination according to federal and Berlin state recommendations	MTX, leflunomide, sulfasalazine, AZA, JAKi, TNFi, abatacept, prednisolone	Median age --> RA Control: 68 years, RTX treated patients: 58 years, healthy control: 57 years; male and female	No data	Pfizer/BioNTech (BNT162b2), moderna (CX-024414), Vaxzevria (ChAdOx1); primary	N/A	IgA titer, IgG titer, neutralizing antibodies, B cells count, T cells count	1) IgA and IgG anti-vaccine titers were significantly diminished on day 7 after the second vaccination in the RA control group and RTX group compared to the healthy control group<br><br>2) Anti-S1 IgG antibodies were detected in 8 (66.7%) of 12 patients in the RA group and 8 (42.1%) of 19 patients in the RTX group. Meanwhile, 5 (41.7%) of 12 patients in the RA group and 9 (47.4%) of 19 patients in the RTX group developed anti-S1 IgA antibodies<br><br>3) Virus-neutralizing antibodies were found in only 8 (66.7%) of the 12 RA control patients and 9 (47.4%) of the 19 RTX-treated patients<br><br>4) No significant difference of RBD-specific B cells was seen between healthy controls, RA patients, and RTX-treated patients<br><br>5) RTX-treated patients who did not show seroconversion after the second vaccination (RTX IgG−) had significantly reduced frequencies and absolute numbers of RBD+ specific B cells compared to IgG+ RTX-treated patients<br><br>6) There was no significant difference regarding the frequency, absolute numbers,	N/A

												or memory formation of CD4 T cells and CD8 T cells between healthy controls, RA controls, and RTX-treated patient	
18	Zabalza, et al (2022)	Spain, multi-center	457 total participants; MS (421), NMOSD (14), MOGAD (5), IgG-4-related diseases (3), AAV (3), MGN (2), MCD (2), primary angiitis of the central nervous system (1), SLE vasculitis (1), myelorradiculitis (1), Tolosa-Hunt syndrome (1), GFAP-encephalitis (1), Susac syndrome (1)	Prospective cohort; 457 patients	Patient with MS on any DMT/untreated & patients with other AIDs currently on anti-CD20s, ≥18 years old, unvaccinated & willing to be vaccinated and without previous known SARS- CoV-2 infection.	no immunosuppressants, β-interferons, GA, teriflunomide, DMF, cladribine, alemtuzumab, natalizumab, S1PRM, aCD20-BCD	≥18 years; male and female	Obesity, lung disease, cardiovascular disease, diabetes, hypertension, haematological benign disease, chronic kidney disease, liver disease, HIV or malignancy	Pfizer/BioNTech (BNT162b2), moderna (mRNA-1273), astrazeneca (AZD1222); primary	N/A	Seroconversion, IgG titer, T cell response	1) Humoral responses were detected in patients with all types of DMTs. The seroconversion rate was >92.0%, except for patients on aCD20-BCD and S1PRM (45.6% and 51.4%, respectively).<br><br>2) Patients on aCD20-BCD and S1PRM presented lower IgG titers compared to those untreated or on other DMTs (p < 0.001)<br><br>3) Cellular responses were detected in 84.4% of patients. All DMTs presented a cellular response rate of >75.0%, except for patients on S1PRM (11%).<br><br>4) Patients on S1PRM presented lower IFN-γ levels compared to those on other DMTs or untreated (p < 0.010 for all comparisons)	N/A
19	Boekel, et al (2021)	Netherlands, single- center	Autoimmune (632); RA (260), PsA (68), AS (68), AxSpa or PrSpA (6), JIA (8), SLE (33), vasculitis (11), polymyalgia rheumatica (37), SJ (33), MS (58), other rheumatic diseases 103 (MCTD, sarcoidosis, SS, and myositis)	Prospective cohort; 632 patients and 289 healthy controls	All adult patients (aged ≥18 years) with chronic inflammatory diseases from the Amsterdam Rheumatology and Immunology Center in Amsterdam. All patients were asked (but not obliged) to recruit their own control participant of the same sex, of comparable age (difference of <5 years), and without a chronic inflammatory disease. In the second study (NCT04498286), adult patients (aged ≥18 years) with multiple sclerosis from the MS Center Amsterdam in Amsterdam.	no immunosuppressants, MTX, tocilizumab, natalizumab, secukinumab, ustekinumab, ixekizumab, abatacept, sulfasalazine, HCQ, leflunomide, AZA, CYC, fingolimod, fampridine, TNFi, aCD20-BCD, β-interferons, prednisone	≥18 years; male and female	Chronic pulmonary disease, cardiovascular disease, diabetes, obesity	Pfizer/BioNTech (BNT162b2), moderna (mRNA-1273), Vaxzevria (ChAdOx1), Janssen (Ad26.COV2.S); primary	There were 94 of 632 patients and 39 of 289 controls who had been infected by COVID-19 after vaccination.	Seroconversion, IgG titer	1) The seroprevalence of IgG SARS-CoV-2 antibodies in patients with autoimmune diseases after their first COVID-19 vaccination was 49% (210 of 432) compared with 73% (154 of 210) in healthy controls (adjusted odds ratio 0·33 [95% CI 0·23–0·48]; p<0·0001;<br><br>2) Patients with autoimmune disease had lower median IgG titres than did controls<br><br>3) Patients treated with MTX and aCD20-BCD therapies had significantly lower seroconversion rates than healthy controls.<br><br>4) Patients on TNFi or prednisone monotherapy and patients not on immunosuppressive therapy did not have lower seroconversion rates than healthy controls.<br><br>5) In patients with previous SARS-CoV-2 infection, IgG antibody titres were not significantly different for patients and controls, which was consistent across different treatment groups	N/A

20	Timmermann, et al (2021)	Switzerland, single-center	118 liver transplant recipient; autoimmune (18)	Cohort;18 patients	Patients are regularly treated at outpatient clinic in a lifelong after-care program regarding, e.g., liver transplant function, development of comorbidities and adjustment of the immunosuppressive therapy.	MMF, tacrolimus, everolimus, CYC, AZA	28 - 89 years; male and female	No data	Pfizer/BioNTech (BNT162b2), moderna (mRNA-1273), Janssen (Ad26.COV2.S); primary	N/A	IgG titer, IgA titer	1) All patients currently not treated with any immunosuppressive medication developed anti-spike protein-IgG-antibodies; 73.1% of all seronegative patients were run on a MMF-based regimen, whereas 22.8% of the seropositive patients were treated with MMF. This finding appears to be statistically significant (p < 0.001)<br><br>2) The immunosuppressive regimen significantly influences both anti-spike-protein-IgG (p = 0.000058) and IgA levels (p = 0.016).<br><br>3) The highest levels for both IgG and IgA were reached in the group weaned off any immunosuppression followed by the group receiving tacrolimus monotherapy<br><br>4) The underlying disease significantly influences the level of anti-spike-protein-IgG (p = 0.005)	N/A
Inactivated virus vaccine and adenovirus vector vaccine (n=2)													
1	Ahmed, et al (2022)	India, single-center	AIRD (630); RA (415), SpA (112), SLE (49), vasculitis (30), SS (18), other CTD (6)	Prospective cohort; 630 patients	Patients with AIRD at the Centre for Arthritis and Rheumatism Excellence in Southern India who had completed both the doses of SARS-CoV-2 vaccines.	MTX, sulfasalazine, leflunomide, apremilast, lenalidomide, AZA, MMF, tacrolimus, HCQ, tofacitinib, TNFi, RTX, colchicine, corticosteroid	Mean age: 55.2 years; male and female	Hypertension, diabetes mellitus, dyslipidemia, coronary artery disease, hypothyroidism, cancer, benign prostatic hypertrophy, bronchial asthma	Covaxin (BBV152), astrazeneca (AZD1222); primary	There were 47 of 630 patients who had COVID-19 breakthrough infection.	IgG titer, neutralization antibodies	1) Based on the subtype of AIRD, the majority of patients with RA were good responders or GR (261, 62.9%) as opposed to patients with SS (8, 44.1%) in whom the majority were non-responders. SLE, vasculitis, other CTDs did not show a significant difference in the response rates.<br><br>2) Among the drugs, only MMF was significantly different between the three groups (good responder, inadequate, and non-responder)<br><br>3) The average neutralisation percentage by sera was significantly higher (p<0.01) for those who did not develop infection (42.9, 95% CI 16.8 to 59.6) compared with those who developed the infection (14.8, 95% CI –12.6 to 39.5)	N/A
2	Cherian, et al (2021)	India, single-center	AIRD (513) [RA (225), SLE (52), SpA (68), scleroderma (16), vasculitis (32), myositis (18), other CTD (22) inflammatory polyarthritis (80)	Cross-sectional survey; 513 AIRD patients and 211 non-AIRD patients	RMD patients who had received first dose of either ChAdOx1 nCov-19 vaccine or the BBV152 vaccine up to 10th of May, 2021	corticosteroids, csDMARDs, bDMARDs	Mean age: 59.9 years; male and female	No data	Covaxin (BBV152)or vaxzevria (ChAdOx1); primary	N/A	N/A	N/A	Four of 513 patients developed autoimmune relapse after vaccination. Local and systemic symptoms occurred in 128 cases and 346 cases among 513 patients and 51 cases and 139 cases among 211 controls, respectively.

mRNA vaccine, inactivated virus vaccine, and adenovirus vector vaccine (n=5)													
1	Assawasaksakul, et al (2021)	Thailand, single-center	SLE (8)	Case-series; 8 healthcare workers	Health care workers who had completed initial COVID-19 vaccine	AZA, CYC, prednisolone, HCQ, MMF, tacrolimus	19-50 years; female	No data	Pfizer/BioNTech (BNT162b2), CoronaVac, vaxzevria (ChAdOx1); primary and booster	N/A	IgG titer, neutralizing antibodies, T cell response	1) Prior to the booster dose, all patients had low-positive antispike antibodies with a median level of 83.3 (IQR 31.6–341.6) U/mL, which rose to a median of 19,986 (IQR 15 079–59 735) U/mL at day 14 after the booster vaccination<br><br>2) Before the booster vaccination, all except patient 7 had negative neutralizing activity results (<35% inhibition). After the booster dose, all patients elicited a strong immune response with at least 95% inhibitin<br><br>3) The majority of patients had strong cellular immune responses, except patients one and three who received more intensive immunosuppressive therapy including MMF, AZA, and calcineurin inhibitor	No autoimmune relapse was occurred after vaccination. Injection site pain was commonly reported, followed by fatigue and fever.
2	Assawasaksakul, et al (2022)	Thailand, single-center	SLE (64), RA (30)	Prospective cohort; 94 patients	SLE and RA patients aged 18–65 years who met the SLE or RA classification criteria	MMF, AZA, tacrolimus, CYC, MTX, leflunomide, prednisolone, antimalarial	18 - 65 years; male and female	No data	Pfizer/BioNTech (BNT162b2), CoronaVac, Sinopharm (BBIBP-CorV), vaxzevria (ChAdOx1); primary	There was 1 patient of each first and second vaccination who had been infected by COVID-19.	Seroconversion, IgG titer, T cell response	1) The inactivated vaccine group had the lowest seroconversion rate (52%) compared to AZD1222 (93%) and AZD1222/BNT162b2 (96%), p < 0.0001.<br><br>2) Anti-RBD titers were lowest in the inactivated vaccine group (2.84 AU/mL; 95% CI 0.96–8.44), followed by AZD1222 or adenovirus vectored (233.7 AU/mL;95% CI 99.0–505.5), and AZD1222/BNT162b2 or heterogenous (688.6 AU/mL; 95% CI 271–1745), p < 0.000)<br><br>3) Positive IGRA test was also lowest in the inactivated vaccine group (27%), followed by the adenovirus-vectored vaccine (67%), and the adenovirus-vectored/mRNA vaccine (73%) (p = 0.03).	No autoimmune relapse was occurred after vaccination. Local symptoms (injection site pain) occurred in 36% of patients. Systemic symptoms, including fatigue and fever, were occurred in 21% of patients.
3	Machado, et al (2021)	UK, single-center	RA (1686), AxSpA (573), PsA (505), PrSpA (114), JIA (7), other inflammatory arthritis (70), SLE (367), APS (26), SJ (223), SS (162), IIM (69), MCTD (37), UCTD (43), Ehler-Danlos syndromes (1), vasculitis (593), IgG4-related disease (16), sarcoidosis (56),	Cohort; 4,604 inflammatory RMDs and 517 non-inflammatory RMDs	Have a pre-existing I-RMD or non-inflammatory rheumatic and musculoskeletal disease (NI-RMD) and have received one or more doses of any vaccine against SARS-CoV-2.	antimalarial, leflunomide, MTX, sulfasalazine, abatacept, BEL, RTX, IL-1 inhibitor, IL-6 inhibitor, IL-12/23 inhibitor, IL-23 inhibitor, IL-17 inhibitor, TNFi, apremilast, JAKi, glucocorticoid, AZA, MMF, CYC,CYP, tacrolimus, IVIG, thalidomide/lenalidomide, colchicine, denosumab, mepolizumab, pembrolizumab, vedolizumab, no immunosuppressants	Mean age: 60.5 years; male and female	No data	Pfizer/BioNTech (BNT162b2), moderna (mRNA-1273), CoronaVac, vaxzevria (ChAdOx1), Janssen (Ad26.COV2.S); primary and booster	There were breakthrough infections in 42 of 4,604 among inflammatory RMD patients and 4 of 517 non-inflammatory RMD patients after vaccination. Among inflammatory RMD patients, there 29 patients who had severe cases, while nine of them were hospitalized.	N/A	N/A	There were 204 of 4,604 patients who developed an autoimmune relapse after vaccination. Systemic symptoms occurred in 1,688 of 4,604 patients after vaccination.<br>Adverse events of special interest including cardiovascular, dermatologic, gastrointestinal, hot flush, anxiety, lower body temperature, haematological, immunological, lymphadenopathy, neurological, other possible cardiac symptoms, pain syndromes, tendons and joints, viral

			relapsing polychondritis (7), other immune-mediated inflammatory diseases (monogenic autoinflammatory syndrome, non-monogenic autoinflammatory syndrome, chronic recurrent multifocal osteomyelitis) (27)									infection occured in 112 of 4,604 patients after vaccination.
4	Mohanasundaram, et al (2022)	India, multi-center	AIRD (2092); RA (1480), SLE (238), AxSpa/PrSpa (99), PsA (77), MCTD (45), SJ (32), SS (26), UCTD (26), vasculitis (18), JIA (8), sarcoidosis (7), APS (6)	Cross-sectional; 1,293 vaccinated patients	All out-patients above 18 years of age and with a confirmed diagnosis of AIRD	MTX, corticosteroid, HCQ, leflunomide, MMF, bDMARDs, iguratimod, AZA, JAKi, CYC	≥18 years; male and female	Diabetes mellitus, thyroid disorder, systemic hypertension, coronary artery disease	Pfizer/BioNTech (BNT162b2), Sinopharm (BBIBP-CorV), Sinovac, Vaxzevria/Covishield (ChAdOx1), Covaxin (BBV 152), Sputnik; primary	There were 72 of 1,293 patients who had been infected by COVID-19. Twenty four of them were hospitalized.	N/A	N/A	There were 32 of 1,293 patients who developed autoimmune relapse after vaccination. Local symptoms occurred in 240 of 1,293 patients after vaccination. Among 1,293 vaccinated patients, systemic symptoms were listed as follows: including fever (328), myalgia (328), joint pain (117) headache (3), fatigue (2), vomiting (1), and weight gain (1).
5	Szebeni, et al (2022)	Hungary, single-center	AIIRD (89); RA (41), PsA (7), AxSpA (12), SLE (11), SJ (4), IIM (1), SS (4), LVV (5), AAV (6), other vasculitis (1)	Prospective observational study; 89 patients and 74 healthy controls	In remission or had a low disease activity, were on stable medication for at least the last eight weeks before enrolment.	glucocorticoid, MTX, leflunomide, AZA, CQ, HCQ, TNFi, IL-6 inhibitor, IL-17 inhibitor, BEL, JAKi	Mean age --> All patients: 59 years, controls: 43 years; male and female	No data	Pfizer/BioNTech (BNT162b2), moderna (mRNA-1273), Sinopharm (BBIBP- CorV), sputnik V (Gam-COVID-Vac), Astrazeneca (AZD1222); primary	There was no case of COVID-19 breakthrough infections.	IgG titer, seroconversion, neutralizing antibodies, T cell response	1) The antibody response was compared between RA and spondylarthropathies (AxSpA and PsA) and autoimmune RMD (SS, SLE, SJ, IIM, LVV, AAV, and Behcet's disease) at one- and four-months post vaccination. The antibody level was higher at four months in spondylarthropathies compared to RA and autoimmune RMD ($p = 0.0048$)<br><br>2) The satisfactory response rate for healthy controls versus patients with autoimmune and inflammatory RMDs was 69% vs. 55% for the inactivated viral vaccine (BBIBP-CorV), 97% vs. 53% for the adenovirus vector-based vaccines (Gam-COVID Vac and AZD1222), and 100% vs. 81% for the mRNA vaccines (BNT162b2 and mRNA-1273), respectively<br><br>3) Within the subgroup of patients on bDMARD therapy, those on B-cell inhibitory treatment showed lower antibody levels both at one- and four-months post vaccination compared to those receiving other biologicals ($p = 0.0074$ and $p = 0.0055$, respectively).<br><br>4) No difference was detected in the studied cellular parameters when RA, spondylarthropathies and autoimmune RMD were compared<br><br>5) There was no statistically	No significant autoimmune relapse was reported. Mild or moderate local and systemic reactions in healthy controls and patients was 189 and 87, respectively. No serious adverse events were observed.

												significant difference in the prevalence of reactive TNF-α or IFN-γ producing CD8+ T cells between healthy controls or patients receiving different vaccine types	

Abbreviations. Therapies: aCD20-BCD: anti-CD20-B cell depleting therapy, ARDs: antirheumatic drugs, AZA: azathioprine, bDMARDs: biologic disease modifying antirheumatic drug, BEL: belimumab, COX: cyclooxygenase, CQ: chloroquine, csDMARDs: conventional synthetic disease modifying antirheumatic drug, CYC: cyclosporine, CYP: cyclophosphamide, DEX: dexamethasone, DMARD: disease modifying antirheumatic drug, DMF: dimethyl fumarate, DMTs: disease-modifying therapies, GA: glatiramer acetate, HCQ: hydroxychloroquine, IL: interleukin, ISP: immunosuppressive therapies, IVIg: Intravenous immunoglobulin, MMF: mycophenolate mofetil, MP: methylprednisolone, MTX: methotrexate, NSAIDs: non-steroidal anti-inflammatory drugs, OCR: ocrelizumab, oDMARDs: other disease modifying antirheumatic drug, PLEX: plasmapheresis, RTX: rituximab, S1PRM: sphingosine-1-phosphate receptor modulators, TNFi: tumor necrosis factor α inhibitor, TPO-RA: thrombopoietin receptor agonists. **Type of diagnosis:** AAV: antineutrophil cytoplasmic antibody (ANCA) associated vasculitis, AID: autoimmune inflammatory disease, AID-IMD: autoimmune and immune-mediated diseases, AIH: autoimmune hepatitis, AIIRD: autoimmune inflammatory rheumatic diseases, AIRD: autoimmune rheumatic disease, ANCA: antineutrophil cytoplasmic antibody, APS: antiphospholipid syndrome, AS: ankylosing spondylitis, ASD: Autoimmune systemic diseases, AxSpA: axial spondyloarthritis, CIA: chronic inflammatory arthritis, CID: chronic inflammatory diseases, CNS: central nervous system, COPD: chronic obstructive pulmonary disease, CTD: connective tissue disease, DM: diabetes mellitus, FSGS: Focal Segmental Glomerulosclerosis, GBM: glomerular basement membrane, GR: good responders, HIV: human immunodeficiency virus, HSCT: hematopoietic stem cell transplantation, IBD: inflammatory bowel disease, IIM: Idiopathic inflammmatory myopathy/myositis, ILD: interstitial lung disease, ILD-SAD: interstitial lung disease and systemic autoimmune disease, IMID: immune mediated inflammatory diseases, IMRD: immune-mediated rheumatic diseases, IS: immunosuppressed, iTTP: Immune-mediated thrombotic thrombocytopenic purpura, JIA: juvenile idiopathic arthritis, LVV: large vessel vasculitis, MCD: Minimal Change Disease, MCTD: mixed connective tissue disease, MGN: Membranous Glomerulopathy, MM: multiple myeloma, MOGAD: myelin oligodendrocyte glycoprotein antibody disorders, MS: multiple sclerosis, NID: neuroinflammatory disease, NMOSD: neuromyelitis optica spectrum disorder, NR: non-responders, OA: osteoarthritis, PAPS: primary antiphospholipid syndrome, PBC: primary biliary cholangitis, pmWS: people with multiple sclerosis, PrSpA: peripheral spondyloarthritis, PsA: psoriatic arthritis, PSC: primary sclerosing cholangitis, RMD: rheumatic and musculoskeletal diseases, SAIRD: systemic autoimmune rheumatic disease, SAMS: systemic autoimmune myopathies, SJ: sjogren syndrome, SLE: systemic lupus erythematous, SOT: solid organ tumors, SpA: spondyloarthritis, SPS: stiff-person syndrome, SS: systemic sclerosis, TMA: Trombotic Microangiopathy, TTS: thrombosis with thrombocytopaenia syndrome, UCTD: Undifferentiated connective tissue disease. **Others:** BAU: binding antibody units, Ag: antigen, CDAI: Chron's Disease Activity Index, DAS28-CRP: Disease Activity Score-28 for Rheumatoid Arthritis, NAb: neutralizing antibodies, sNAb: surrogate neutralizing antibody, GMT: geometric mean neutralizing titers, IFN: interferon, IGRA: interferon-γ releasing assay, SC: seroconversion, S1: spike-1, S2: spike-2, IgG: immunoglobulin G, RBD: receptor-binding domain, SFCs: spot forming cells, PBMC: peripheral blood mononuclear cell, ID: inhibitory dilution, fi-GMT: factor-increase in GMT, GMT: geometric mean titer, nCoV-19: novel Coronavirus-19,

SARS-CoV-2: severe acute respiratory syndrome coronavirus 2, Anti-PF4: Antiplatelet factor 4, IgGAM: IgG/A/M, Tfh-like cells: T follicular helper cells, VOC: variant of concern, IQR: interquartile range, CG: control groups, CTRL: control, CDAI: clinical disease activity index, D69: day 69, HV: HC: healthy control, HCWs: health care workers, healthy volunteer, ns: not significant.

Table S3. GRADE assessment

Summary of findings:

Patients with autoimmune diseases compared to healthy controls for COVID-19 vaccination

Outcomes	Anticipated absolute effects* (95% CI)		Relative effect (95% CI)	№ of participants (studies)	Certainty of the evidence (GRADE)	Comments
	**Risk with healthy controls**	**Risk with Patients with autoimmune diseases**				
Breakthrough COVID-19 Infections after Inactivated Vaccination	20 per 1,000	**39 per 1,000** (23 to 67)	**RR 1.93** (1.14 to 3.29)	3174 (4 observational studies)	⊕⊕⊕◯ Moderate[a]	Patients with autoimmune diseases probably increase breakthrough COVID-19 infections after inactivated vaccination slightly.
Breakthrough COVID-19 Infections after mRNA or Adenovirus Vector Vaccination	253 per 1,000	**246 per 1,000** (215 to 281)	**RR 0.97** (0.85 to 1.11)	3093 (2 observational studies)	⊕⊕⊕◯ Moderate[b,c]	Patients with autoimmune diseases may result in little to no difference in breakthrough COVID-19 infections after mRNA or adenovirus vector vaccination.
TAb Titers after mRNA Vaccination	-	SMD **0.11 SD lower** (0.2 lower to 0.02 lower)	-	2626 (7 observational studies)	⊕⊕⊕⊕ High	Patients with autoimmune diseases reduce TAb titers after mRNA vaccination slightly.
TAb Titers after Inactivated Vaccination	-	SMD **0.1 SD lower** (0.19 lower to 0)	-	2234 (5 observational studies)	⊕⊕⊕⊕ High	Patients with autoimmune diseases result in little to no difference in TAb titers after inactivated vaccination.
IgG Seroconversion after mRNA Vaccination	991 per 1,000	**812 per 1,000** (743 to 892)	**RR 0.82** (0.75 to 0.90)	3750 (11 observational studies)	⊕⊕⊕◯ Moderate[d]	Patients with autoimmune diseases probably reduce IgG Seroconversion after mRNA vaccination slightly.
IgG Seroconversion after Inactivated Vaccination	960 per 1,000	**739 per 1,000** (681 to 806)	**RR 0.77** (0.71 to 0.84)	3557 (7 observational studies)	⊕⊕⊕◯ Moderate[e]	Patients with autoimmune diseases reduce IgG Seroconversion after inactivated vaccination slightly.
Neutralizing Antibodies after mRNA Vaccination	1,000 per 1,000	**790 per 1,000** (540 to 1,000)	**RR 0.79** (0.54 to 1.14)	223 (3 observational studies)	⊕◯◯◯ Very low[a,b,d]	Patients with autoimmune diseases may reduce/have little to no effect on neutralizing antibodies after mRNA vaccination but the evidence is very uncertain.

Summary of findings:

Patients with autoimmune diseases compared to healthy controls for COVID-19 vaccination

Outcomes	Anticipated absolute effects* (95% CI)		Relative effect (95% CI)	№ of participants (studies)	Certainty of the evidence (GRADE)	Comments
	**Risk with healthy controls**	**Risk with Patients with autoimmune diseases**				
Neutralizing Antibodies after Inactivated Vaccination	840 per 1,000	**596 per 1,000** (571 to 621)	**RR 0.71** (0.68 to 0.74)	3594 (7 observational studies)	⨁⨁⨁⨁ High	Patients with autoimmune diseases reduce neutralizing Antibodies after Inactivated Vaccination slightly.
Neutralizing Activity after Inactivated Vaccination	-	SMD **0.52 SD lower** (1.34 lower to 0.3 higher)	-	2501 (5 observational studies)	⨁◯◯◯ Very low[a,b,c,f]	Patients with autoimmune diseases may reduce/have little to no effect on neutralizing activity after inactivated vaccination but the evidence is very uncertain.
Local Adverse Events after 1st Dose Inactivated Vaccination	193 per 1,000	**243 per 1,000** (203 to 291)	**RR 1.26** (1.05 to 1.51)	2471 (4 observational studies)	⨁⨁⨁⨁ High	Patients with autoimmune diseases increase local adverse events after 1st dose after inactivated vaccination slightly.
Local Adverse Events after 2nd Dose Inactivated Vaccination	170 per 1,000	**189 per 1,000** (155 to 230)	**RR 1.11** (0.91 to 1.35)	2451 (4 observational studies)	⨁⨁⨁⨁ High	Patients with autoimmune diseases result in little to no difference in local adverse events after 2nd dose inactivated vaccination.
Systemic Adverse Events after 1st Dose Inactivated Vaccination	330 per 1,000	**432 per 1,000** (379 to 488)	**RR 1.31** (1.15 to 1.48)	2471 (4 observational studies)	⨁⨁⨁⨁ High	Patients with autoimmune diseases increase systemic adverse events after 1st dose inactivated vaccination slightly.
Systemic Adverse Events after 2nd Dose Inactivated Vaccination	275 per 1,000	**311 per 1,000** (242 to 399)	**RR 1.13** (0.88 to 1.45)	2451 (4 observational studies)	⨁⨁⨁◯ Moderate[g]	Patients with autoimmune diseases probably result in little to no difference in systemic adverse events after 2nd dose inactivated vaccination.

***The risk in the intervention group** (and its 95% confidence interval) is based on the assumed risk in the comparison group and the **relative effect** of the intervention (and its 95% CI).

CI: confidence interval; **RR:** risk ratio; **SMD:** standardised mean difference

GRADE Working Group grades of evidence
High certainty: we are very confident that the true effect lies close to that of the estimate of the effect.
Moderate certainty: we are moderately confident in the effect estimate: the true effect is likely to be close to the estimate of the effect, but there is a possibility that it is substantially different.
Low certainty: our confidence in the effect estimate is limited: the true effect may be substantially different from the estimate of the effect.
Very low certainty: we have very little confidence in the effect estimate: the true effect is likely to be substantially different from the estimate of effect.

Explanations

a. Wide range of confidence interval

b. Only few studies reported this outcome

c. Subgroup data could not be obtained

d. Higgins I2 showed 97% (considerable heterogeneity)

e. Higgins I2 showed 86% (considerable heterogeneity)

f. Higgins I2 showed 98% (considerable heterogeneity)

g. Higgins I2 showed 62% (considerable heterogeneity)

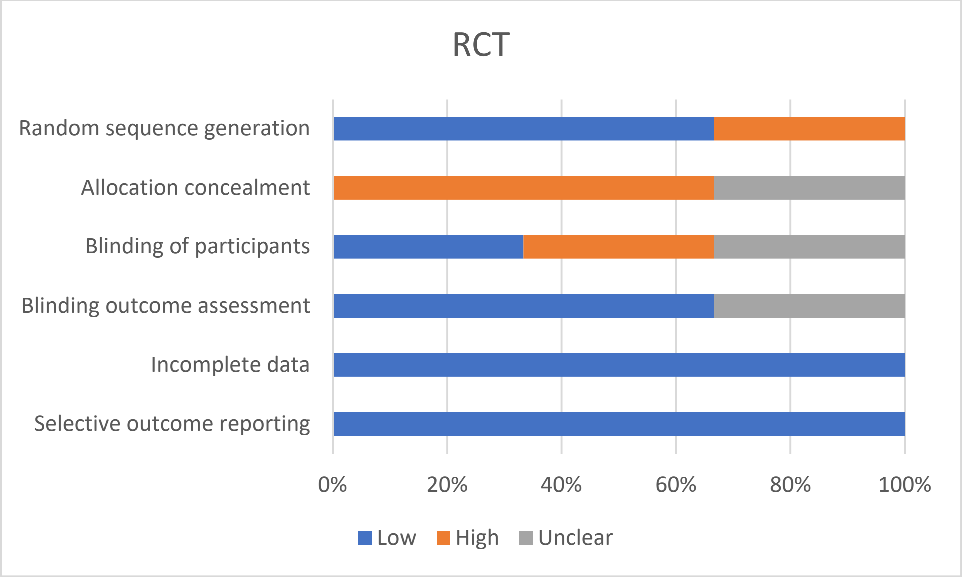

Figure S1A. Quality assessment of RCT studies

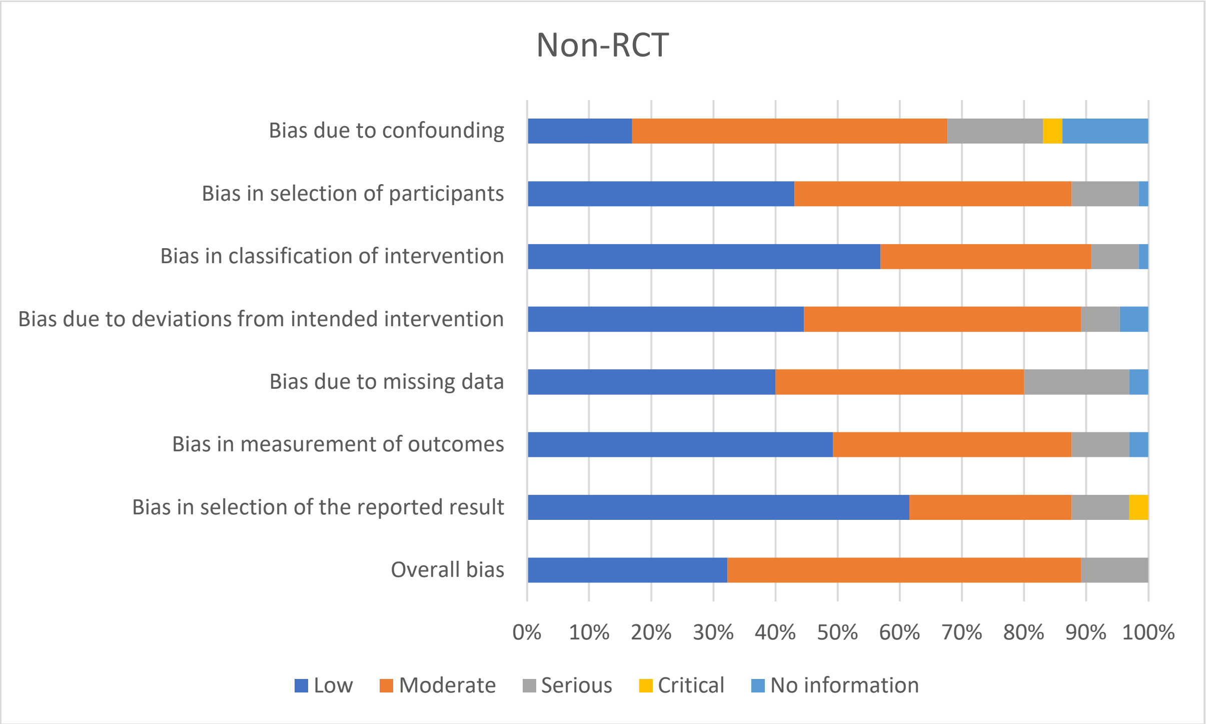

Figure S1B. Quality assessment of non-RCT studies

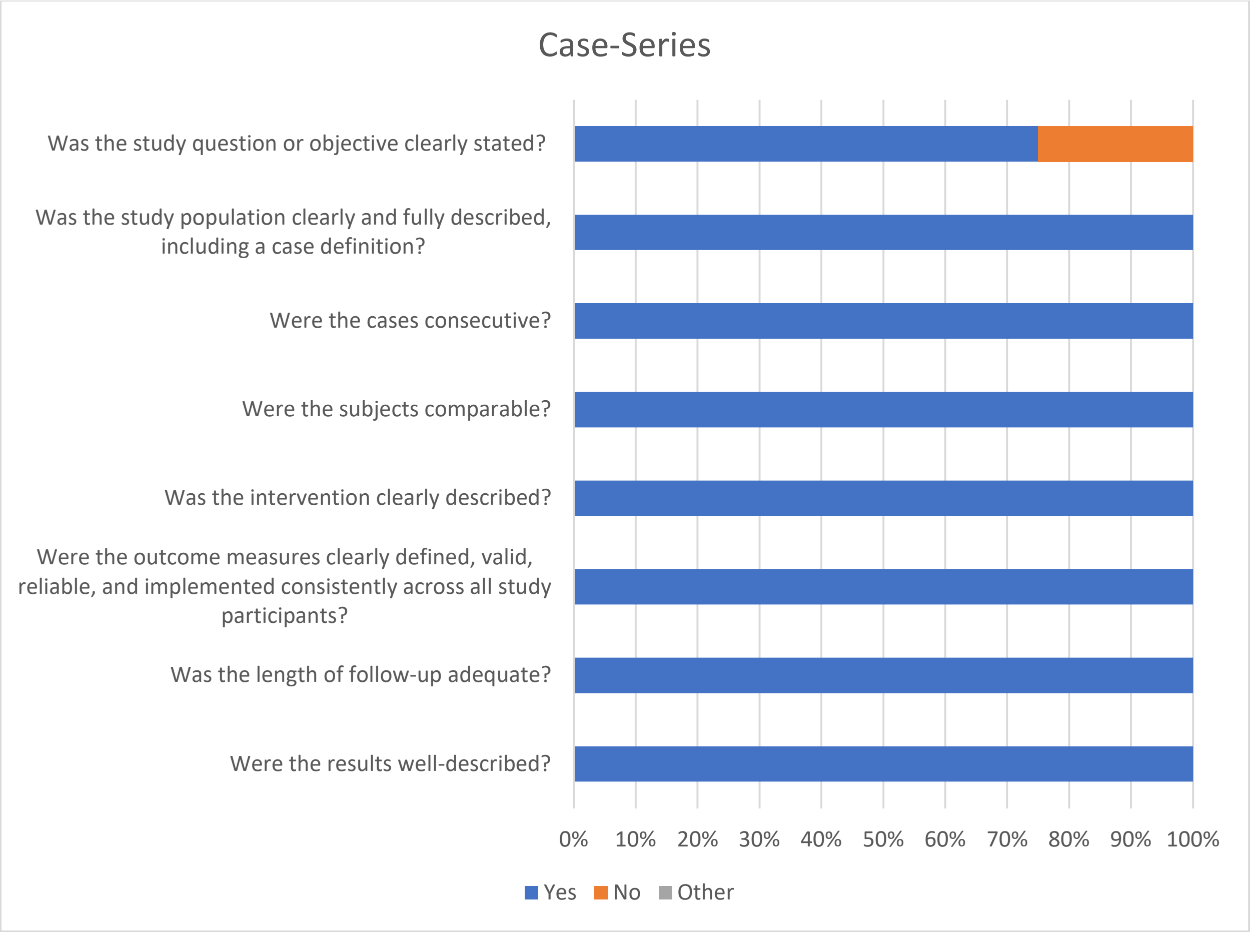

Figure S1C. Quality assessment of case-series studies

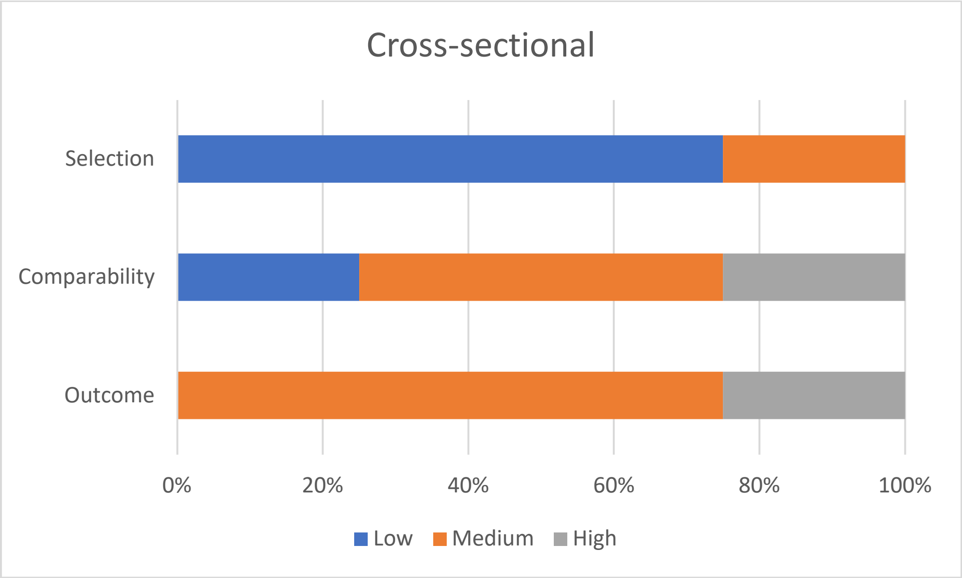

Figure S1D. Quality assessment of cross-sectional studies

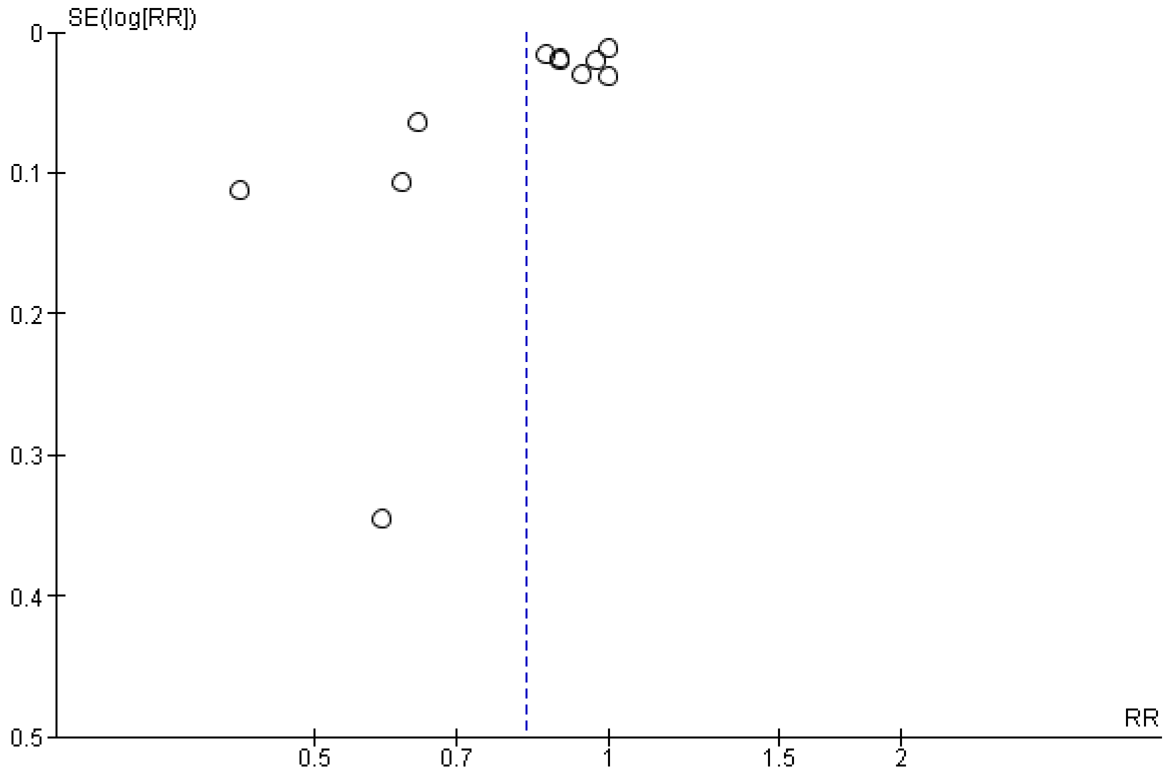

Figure S2. Funnel plot of IgG seroconversion after mRNA vaccination
